# Supplementary material for: Mapping cells through time and space with moscot
Source: Nature. 2025 Jan 22;638(8052):1065–75. doi: 10.1038/s41586-024-08453-2 (PMC11864987; doi:10.1038/s41586-024-08453-2)
Supplement: Supplementary file 1 — Supplementary Figs. 1–43 (displaying additional results) and Supplementary Notes 1–8 (discussing further methods and related work). [file 41586_2024_8453_MOESM1_ESM.pdf]

nature portfolio

https://doi.org/10.1038/s41586-024-08453-2

Supplementary information

Mapping cells through time and space with moscot

In the format provided by the authors and unedited

Supplementary Figures

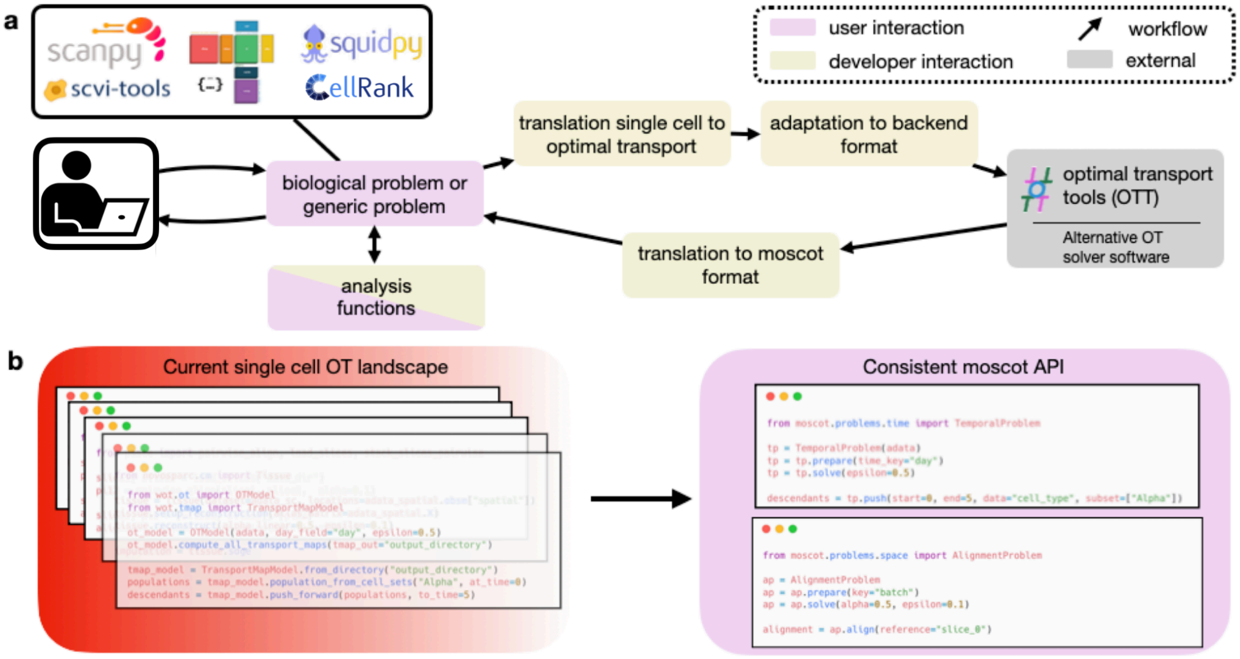

Supplementary Fig. 1 | Moscot provides unified access to various problems in single-cell genomics

a. The moscot workflow. The user interacts with a biological problem, which moscot translates into an OT problem and solves in the backend using Optimal Transport Tools (OTT[1]). Moscot then presents the solution to the user, who can further analyze it using downstream analysis functions. **b.** Comparison of code required to solve different biological problems in the current OT landscape and in moscot. Through its consistent API, moscot is user-friendly and easy to extend.

a

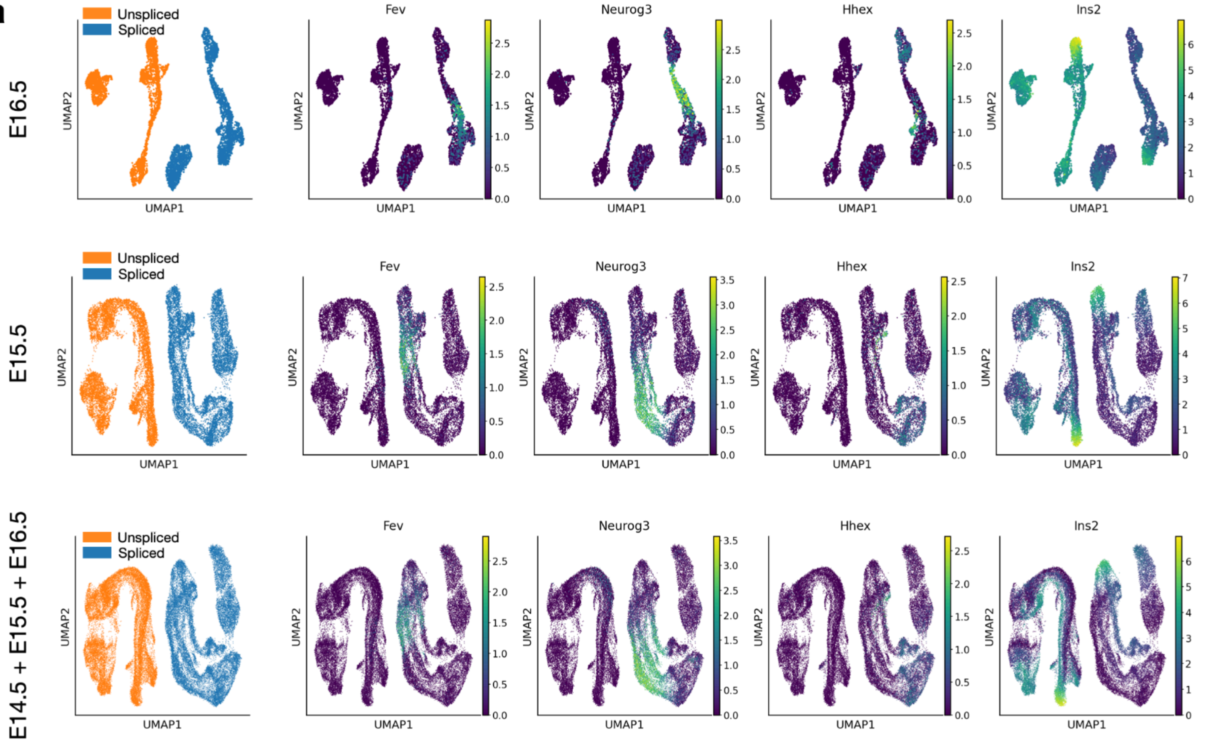
E16.5
E15.5
E14.5 + E15.5 + E16.5
Unspliced
Spliced
Fev
Neurog3
Hhex
Ins2
UMAP1
UMAP2

b

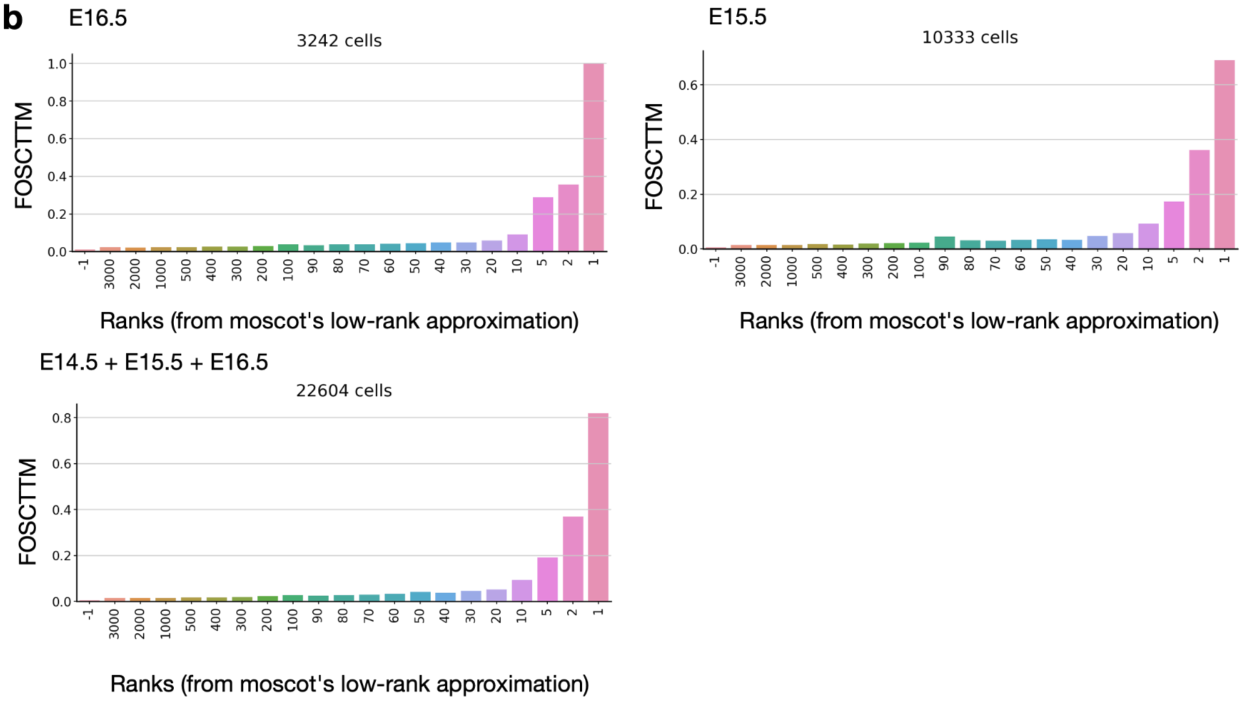
E16.5
3242 cells
E15.5
10333 cells
E14.5 + E15.5 + E16.5
22604 cells
FOSCTTM
Ranks (from moscot's low-rank approximation)

Supplementary Fig. 2 | moscot's performance on aligning cells from spliced/unspliced counts is robust with respect to low-rank approximations

a. UMAP embeddings of different subsets (embryonic day (E)16.5, E15.5, and E14.5+E15.5+E16.5, i.e. full dataset) of the pancreas dataset published with this manuscript. Cells are split into spliced and unspliced counts, resulting in two distinct populations of cells in which a known one-to-one match exists. Normalized gene expression of relevant marker genes in the mouse pancreas is visualized to demonstrate the difference of gene expression of spliced and unspliced RNA counts. **b.** Fraction of cells closer (or equally close) to their true match (FOSCTTM[2]) score, computed by calculating the fraction of cells which are equally or more likely to be assigned to a single cell than their true match based on the entries of the transport matrix. A value of 0 denotes a perfect match, while a value of 1.0 is as good as the outer (independent) coupling.

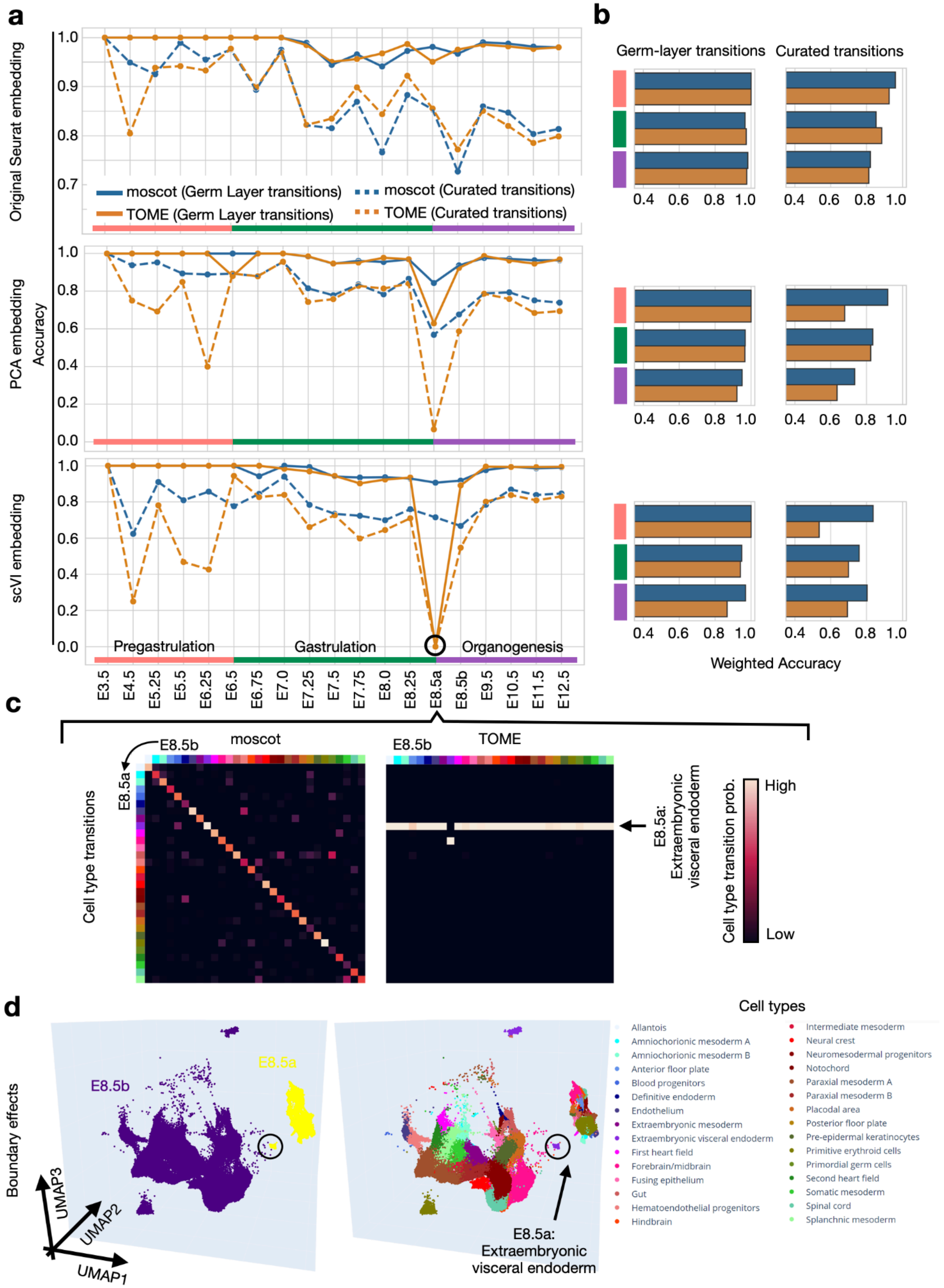
a
b
c
d
Original Seurat embedding
PCA embedding
scVI embedding
Accuracy
moscot (Germ Layer transitions)
moscot (Curated transitions)
TOME (Germ Layer transitions)
TOME (Curated transitions)
Pregastrulation
Gastrulation
Organogenesis
E3.5
E4.5
E5.25
E5.5
E6.25
E6.5
E6.75
E7.0
E7.25
E7.5
E7.75
E8.0
E8.25
E8.5a
E8.5b
E9.5
E10.5
E11.5
E12.5
Germ-layer transitions
Curated transitions
Weighted Accuracy
moscot
TOME
E8.5b
E8.5a
Cell type transitions
E8.5a: Extraembryonic visceral endoderm
Cell type transition prob.
High
Low
Cell types
Boundary effects
UMAP1
UMAP2
UMAP3
Allantois
Amniochorionic mesoderm A
Amniochorionic mesoderm B
Anterior floor plate
Blood progenitors
Definitive endoderm
Endothelium
Extraembryonic mesoderm
Extraembryonic visceral endoderm
First heart field
Forebrain/midbrain
Fusing epithelium
Gut
Hematoendothelial progenitors
Hindbrain
Intermediate mesoderm
Neural crest
Neuromesodermal progenitors
Notochord
Paraxial mesoderm A
Paraxial mesoderm B
Placodal area
Posterior floor plate
Pre-epidermal keratinocytes
Primitive erythroid cells
Primordial germ cells
Second heart field
Somatic mesoderm
Spinal cord
Splanchnic mesoderm

Supplementary Fig. 3 | Moscot is robust to changes in latent cell space representation

a,**b**. Comparing moscot.time and TOME in terms of the germ-layer and curated transition metrics of Figure 2, for individual time points (**a**) and aggregated over time-windows (**b**), for the original Seurat embedding of ref.[3] (top row), a PCA embedding (middle row) and an scVI embedding (bottom row; Methods). **c**. Heatmaps of cell-type backwards transition probabilities for moscot (left) and TOME (right) on the E8.5a/b pair of time points where TOME performs worse than moscot in PCA and scVI representations. The letters a/b correspond to different experimental technologies employed at the same time point as a "bridge" (Methods). Colors correspond to clusters in (**d**). **d**. 3D UMAP embedding, used by the TOME algorithm for k-NN graph construction[3], colored by time points (left) and cell types (right). Circle and arrow highlight a cluster of E8.5a extraembryonic visceral endoderm cells, which TOME falsely connects to almost all E8.5b cells because it is an outlier in the 3D UMAP. Moscot avoids such boundary effects by using higher-dimensional representations and by weakly enforcing probability mass conservation (Methods).

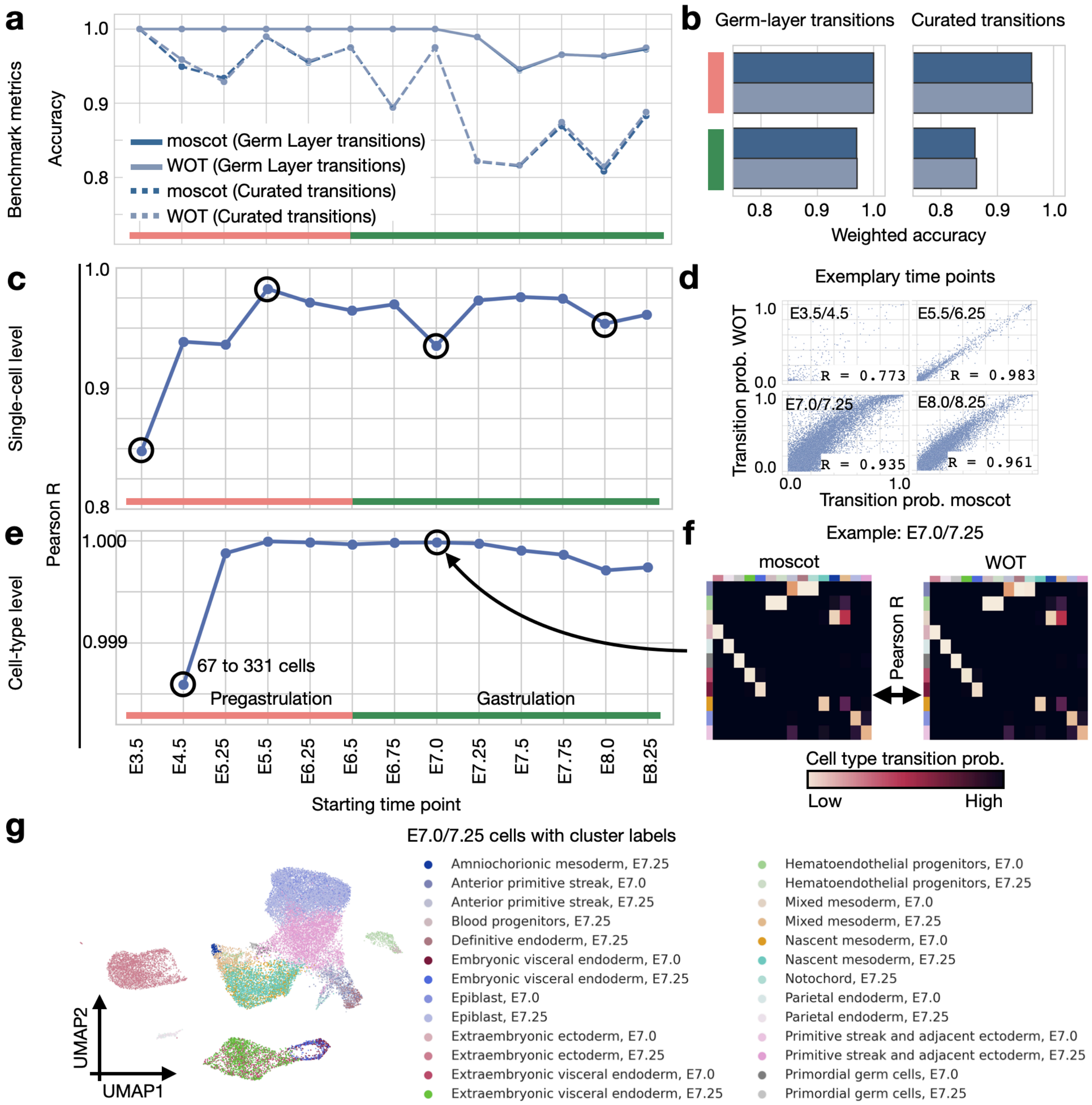

Supplementary Fig. 4 | Moscot reproduces Waddington OT results

Moscot.time versus Waddington OT (WOT) in terms of our benchmark metrics (**a**,**b**), and on the level of single-cell (**c**,**d**) and cell-type (**e**-**g**) transitions. Throughout this figure, we show earlier time points (Pre-gastrulation and Gastrulation) containing few enough cells for WOT to run (Methods). **a**,**b**. Comparing moscot.time and Waddington OT (WOT) in terms of the germ-layer and curated transition metrics of Figure 2, for individual time points (**a**) and aggregated over time-windows (**b**). Accuracy is weighted by the number of cell states per time point (Methods). **c**. Pearson correlation (y-axis) of moscot.time with WOT normalized coupling matrix entries as a function of time (x-axis; Methods). **d**. Scatter plots, illustrating the four exemplary time point pairs indicated in (**c**) with circles. **e.** As in (**c**), but aggregated to the cell-type level. We omit the first pair of time points, as they only contain a single cell state. **f.** Heatmaps of moscot.time (left)

and WOT (right) backwards cell-type transitions for the E7.0/7.25 time point pair, indicated in (**e**) with a circle. Row and column colors correspond to (**g**). **g**. UMAP of the E7.0/7.25 time point, colored by cell types.

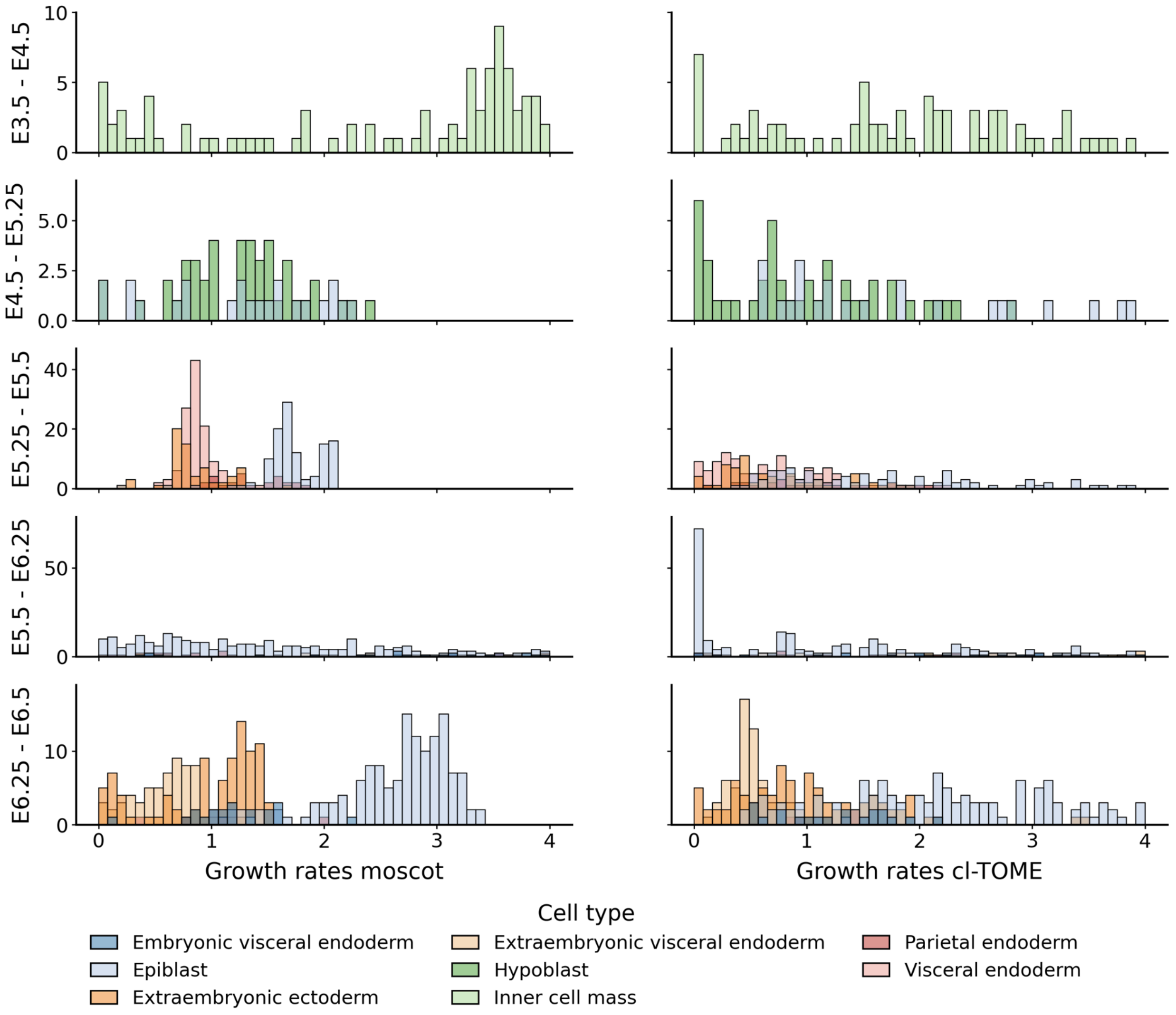

Supplementary Fig. 5 | Comparison of predicted pre-gastrulation growth rates for moscot and cl-TOME

Histograms over moscot and cl-TOME predicted growth rates, grouped by cell type (Methods). Growth rates correspond to the predicted amount of descendants for each cell for the transition from the earlier to the later time point. Growth rates bigger or smaller than one correspond to cell proliferation or growth, respectively.

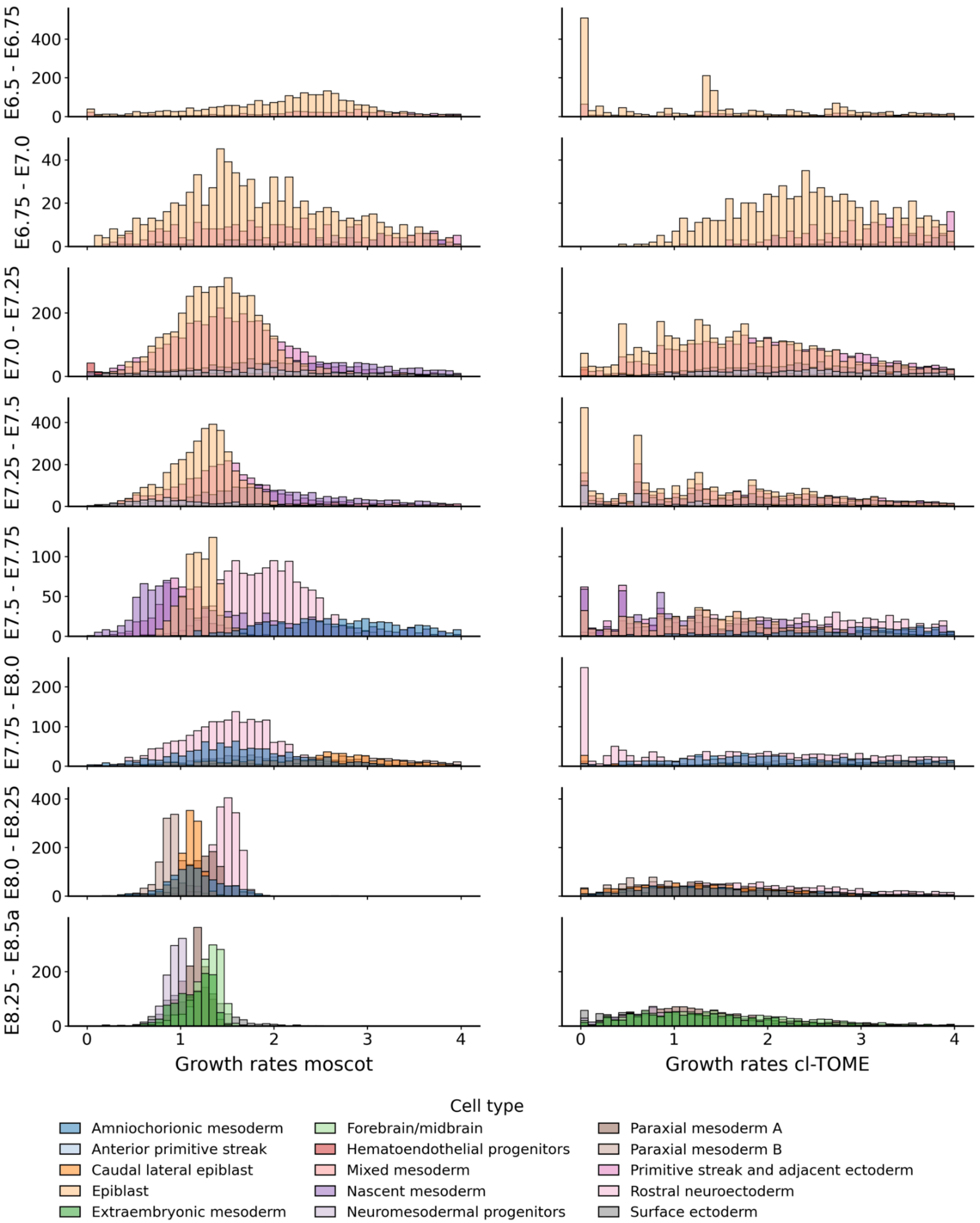

E6.5 - E6.75
E6.75 - E7.0
E7.0 - E7.25
E7.25 - E7.5
E7.5 - E7.75
E7.75 - E8.0
E8.0 - E8.25
E8.25 - E8.5a
Growth rates moscot
Growth rates cl-TOME
Cell type
Amniochorionic mesoderm
Anterior primitive streak
Caudal lateral epiblast
Epiblast
Extraembryonic mesoderm
Forebrain/midbrain
Hematoendothelial progenitors
Mixed mesoderm
Nascent mesoderm
Neuromesodermal progenitors
Paraxial mesoderm A
Paraxial mesoderm B
Primitive streak and adjacent ectoderm
Rostral neuroectoderm
Surface ectoderm

Supplementary Fig. 6 | Comparison of predicted gastrulation growth rates moscot and cl-TOME

See the description of Supplementary Fig. 5 .

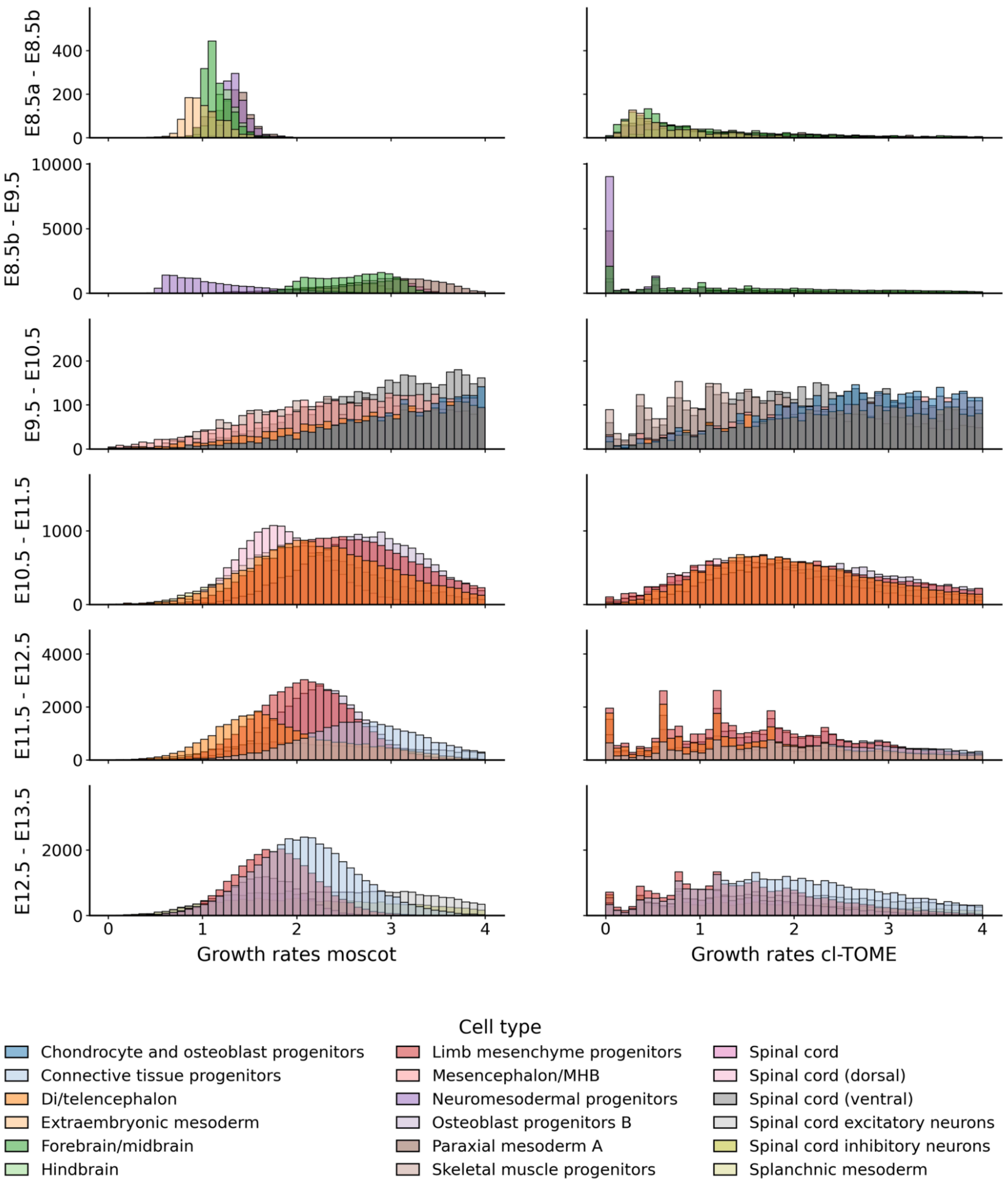

Supplementary Fig. 7 | Comparison of predicted organogenesis growth rates moscot and cl-TOME

See the description of Supplementary Fig. 5 . Additionally, at E8.5a/b, no experimental time passes but the experimental protocol switches from 10x genomics to sci-RNA-seq3[3].

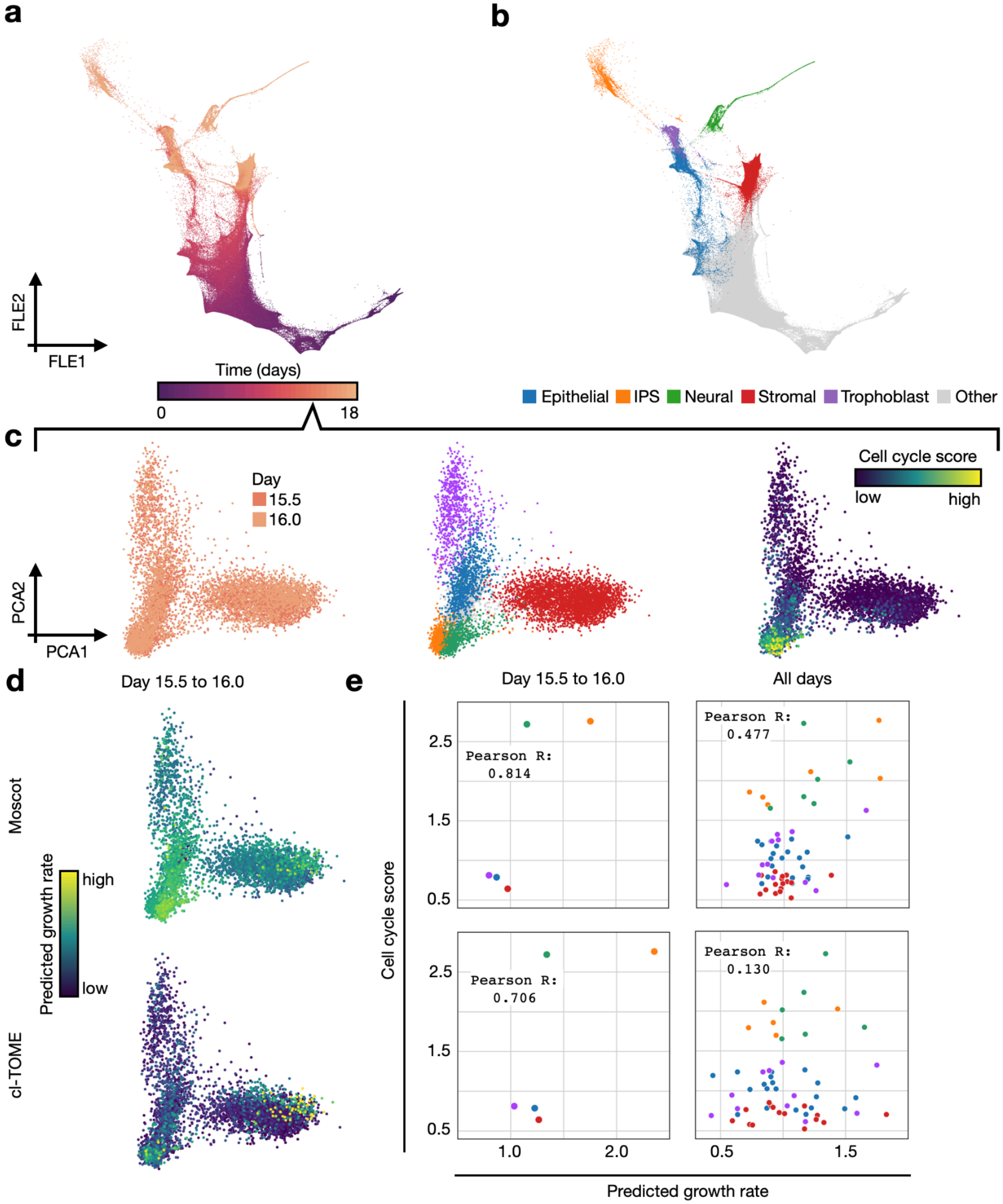

Supplementary Fig. 8 | Predicted growth rates correlate well with cell cycle scores

a,**b**. Force-directed layout (FLE) of 165,892 cells across 39 time points, spanning days 0 to 18 of a reprogramming time course[4], colored by experimental time point (**a**) and major cell sets (**b**). **c**. PCA embeddings of day 15.5/16.0 cells, colored by experimental time point (left), major cell sets as in (**b**) (middle), and scanpy-computed cell cycle scores[5] (right). **d**. PCA embeddings of

the cell subset of (**c**), colored by moscot.time (top) and cl-TOME (bottom) predicted growth rates (Methods). **e**. Scatter plots of predicted (x-axis) versus scanpy-computed (y-axis) growth rates, averaged over the major cell sets of (**b**), for moscot (top) and cl-TOME (bottom), over the 15.5/16.0 time point (left) and all time points (right).

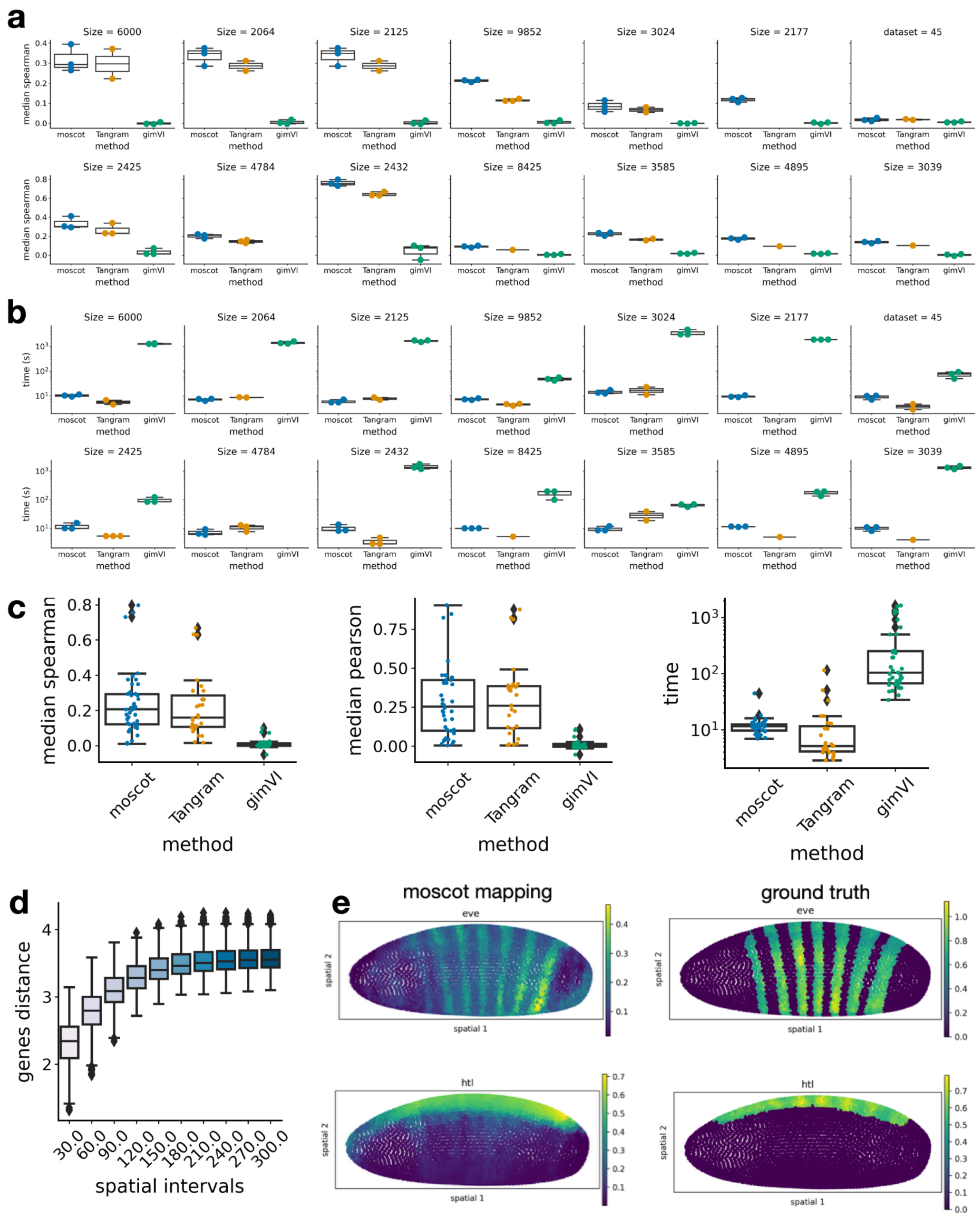

Supplementary Fig. 9 | Multimodal mapping of CITE-seq data to spatial data

a. Spearman correlation coefficient of predicted genes across three seeds for each method and dataset. **b.** Run times across three seeds for each method and dataset. **c.** Left: Spearman

correlation of predicted gene expression aggregated across datasets, summarizing panel (**a**). Center: Pearson correlation of predicted gene expression aggregated across datasets. Right: run time aggregated across dataset, summarizing panel (**b**). **d.** Spatial correspondence: The x-axis represents the spatial (Euclidean) distances and the y-axis represents the gene expression (Euclidean) distances across all genes for all cells within the corresponding spatial distance interval. The Spearman correlation is computed between the gene expression distance and the spatial distance. The spatial correspondence showcased here was computed from the drosophila embryo dataset from Li et al.[6] **e.** Example of moscot.space's mapping for the dataset with the highest correlation values (3rd from top left in (**a**)) with ground truth and predicted expression of two genes: *eve* and *htl*.

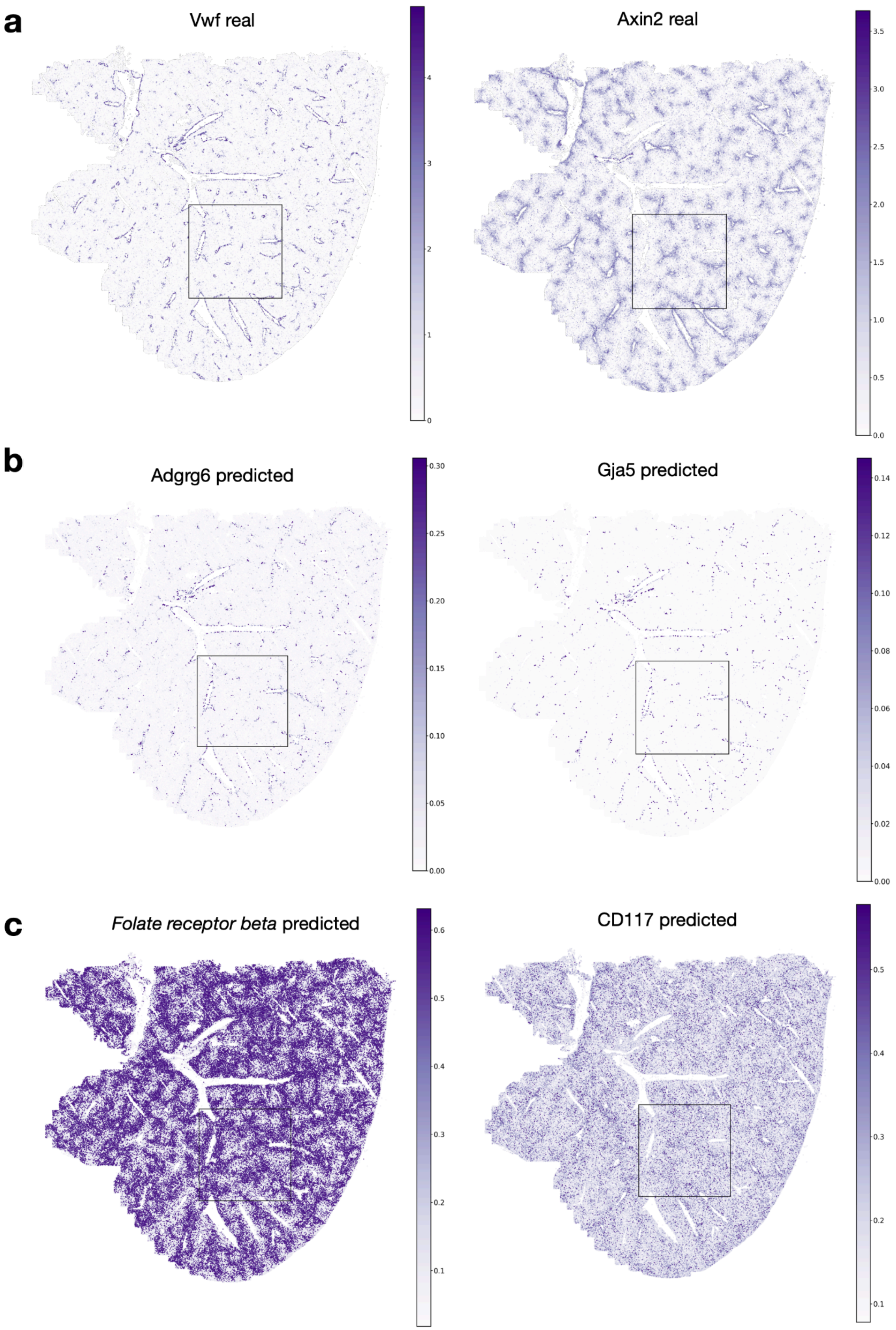
a
Vwf real
Axin2 real
b
Adgrg6 predicted
Gja5 predicted
c
Folate receptor beta predicted
CD117 predicted

Supplementary Fig. 10 | Multimodal mapping of CITE-seq data to spatial data

a. Spatial visualization of ground truth genes used to identify veins with *Vwf* (endothelial cells marker) and central veins with *Axin2* (hepatocytes, endothelial cells marker). **b.** Spatial visualization of predicted expression of genes *Adgrg6* and *Gja5*, markers of endothelial cells associated with portal veins. **c.** Spatial visualization of predicted expression of proteins *Folate receptor beta* (marker of Kupffer cells) and *CD117* (marker of endothelial cells associated with central veins). Boxes in solid lines correspond to insets in Figure 3.

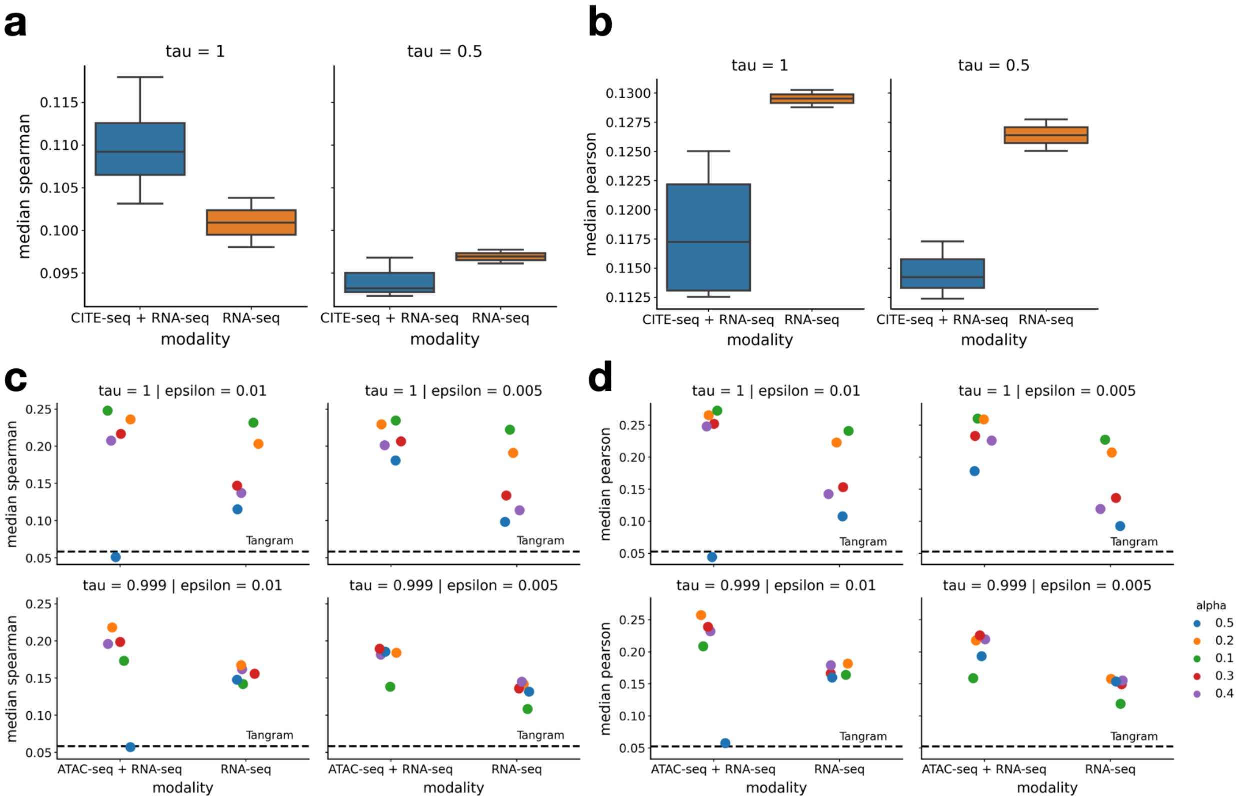

Supplementary Fig. 11 | moscot.space.mapping improves performance when including additional modality

a. Median Spearman correlation of all genes in the mouse liver dataset (Figure 3) using only RNA-seq or CITE-seq + RNA-seq information for the mapping. Box plots represent at least 3 runs with different initializers and ranks. **b.** Same as (**a**), but measured with Pearson correlation. **c.** Median Spearman correlation for the 70 chromatin accessibility peaks (10 for each cluster, 7 clusters) using RNA-seq or RNa-seq + ATAC-seq for the spatial multiomics dataset. Each point is a different value of alpha (weighing factor for the linear and quadratic term). **d.** Same as (**c**), but median Pearson correlations.

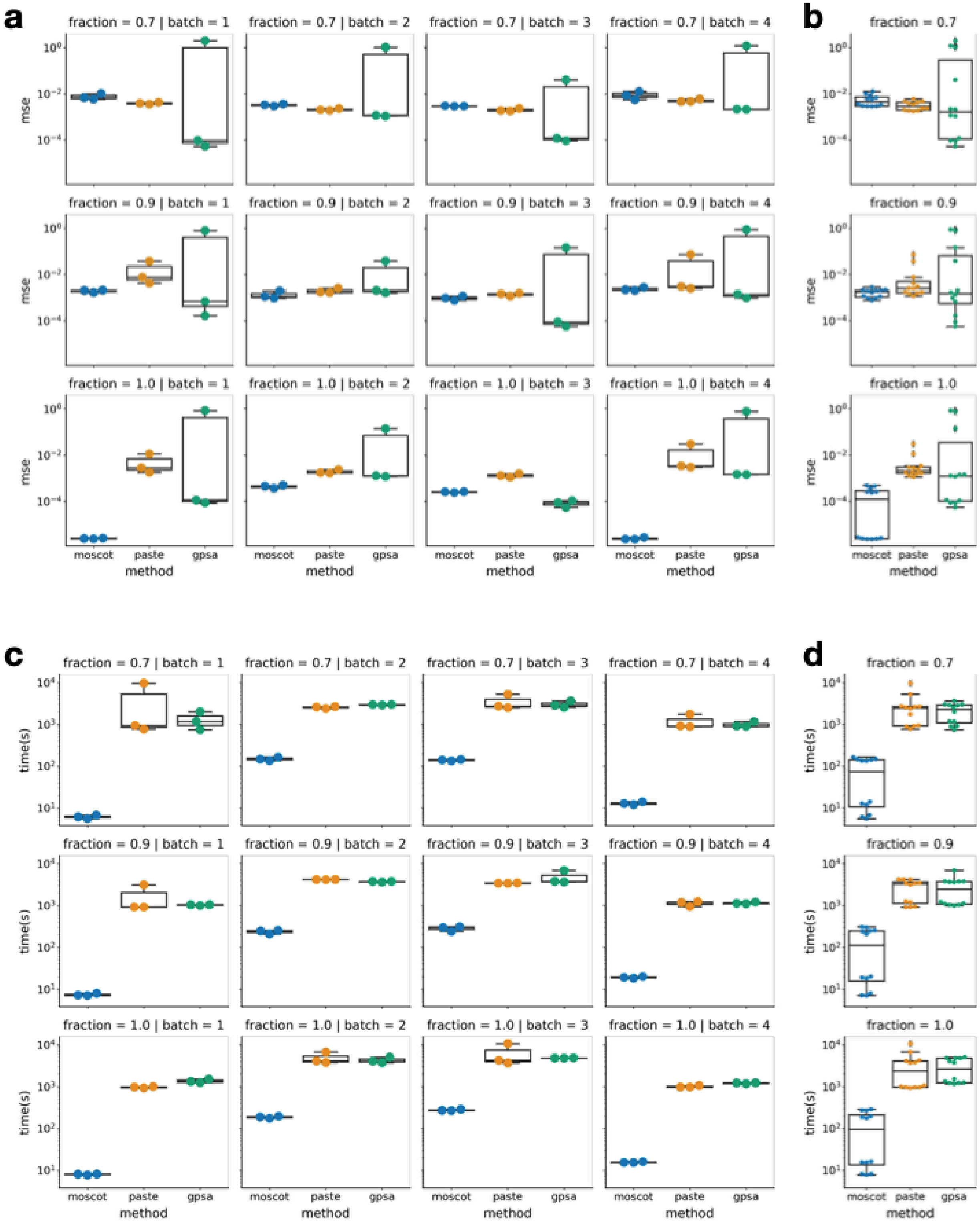

a
fraction = 0.7 | batch = 1
fraction = 0.7 | batch = 2
fraction = 0.7 | batch = 3
fraction = 0.7 | batch = 4
fraction = 0.9 | batch = 1
fraction = 0.9 | batch = 2
fraction = 0.9 | batch = 3
fraction = 0.9 | batch = 4
fraction = 1.0 | batch = 1
fraction = 1.0 | batch = 2
fraction = 1.0 | batch = 3
fraction = 1.0 | batch = 4
mse
moscot
paste
gpsa
method
b
fraction = 0.7
fraction = 0.9
fraction = 1.0
c
time(s)
d

Supplementary Fig. 12 | Benchmark of spatial alignment methods

a. Mean squared error (mse) of aligned spots in spatial coordinates across three random seeds for PASTE[7], GPSA[8] and moscot. The columns of the grid correspond to four different simulated datasets, the rows correspond to different fractions of subsampling (1. means no subsampling and the number of points in source and target is exactly the same, 0.7 means that only 70% of the points in both source and target have been kept for the alignment. Subsampling was done to simulate a more realistic noisy scenario). **b.** Aggregated results across datasets. **c.-d.** Run time (**c**) and aggregated run time (**d**) of different methods for the experiments outlined before (datasets in columns, fraction of subsampling in rows, last column represents summary over rows).

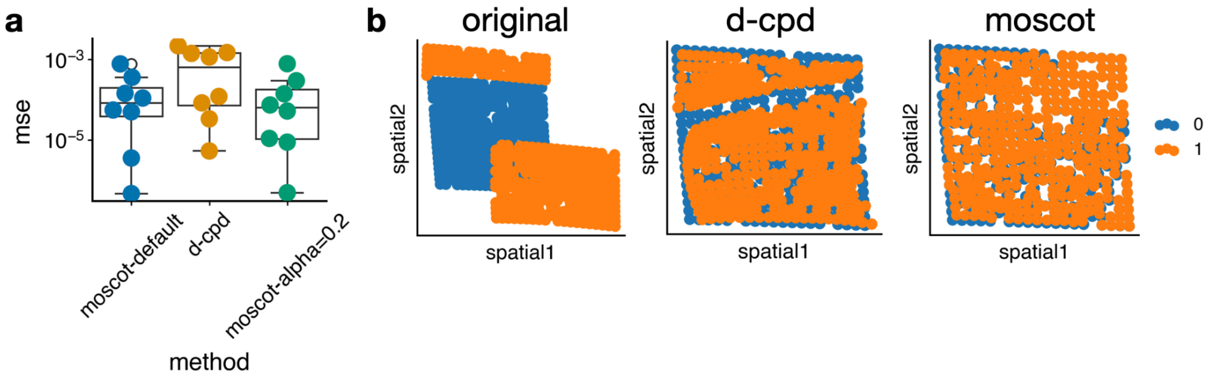

Supplementary Fig. 13 | moscot's spatial alignment method is superior to alignment with deformable-coherent point drift

a. Benchmark across 8 synthetic datasets for moscot-default (default parameters of moscot), d-cpd[9] (default parameters) and moscot-alpha=0.2 (alpha parameter is set to 0.2). **b.** Example of the original alignment problem for the two sets of points clouds (source in orange, target in blue), and the result for d-cpd and the default settings for moscot.space.alignment.

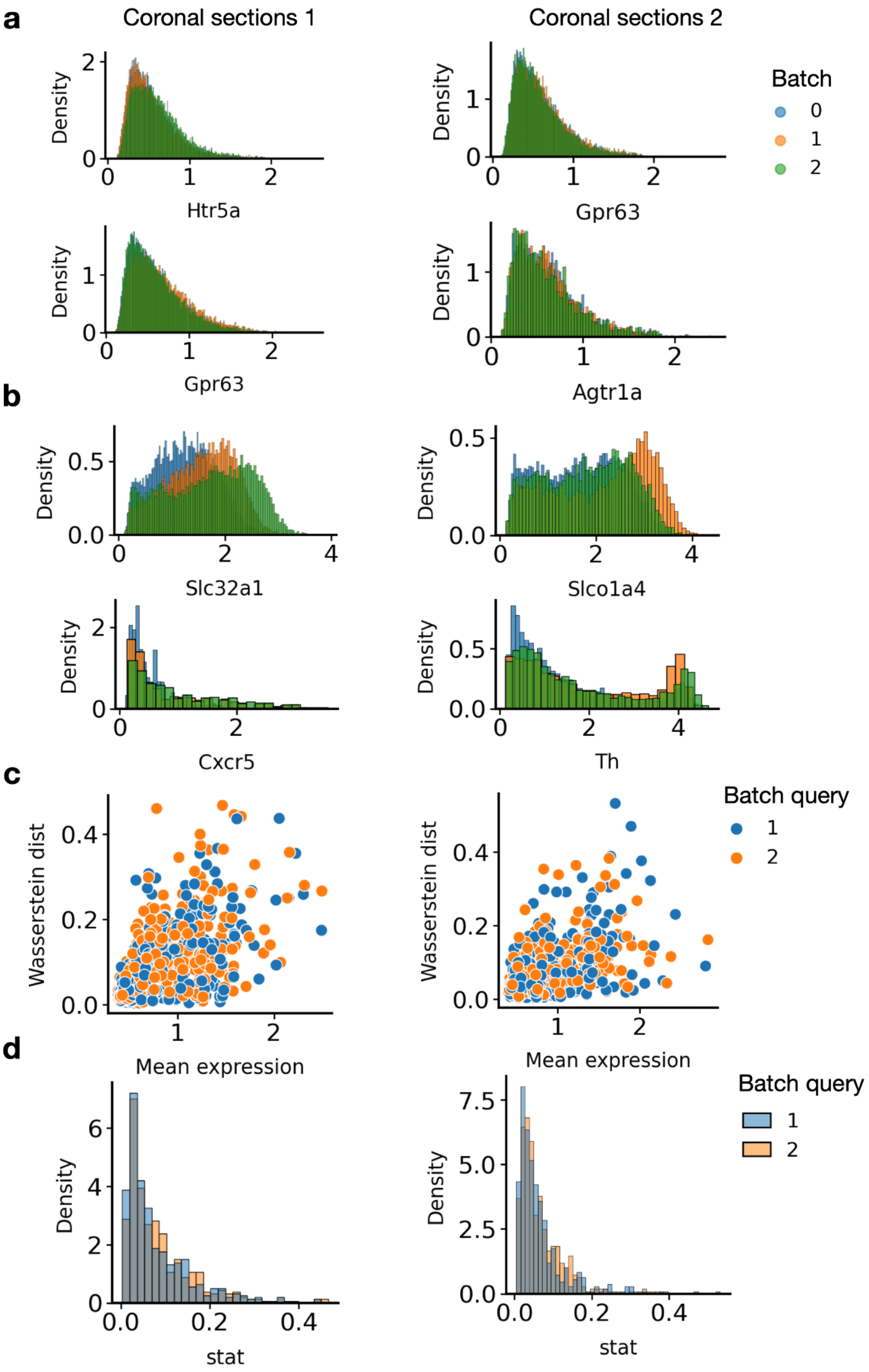
a
Coronal sections 1
Coronal sections 2
Batch
0
1
2
Density
Htr5a
Gpr63
Gpr63
Agtr1a
b
Slc32a1
Slco1a4
Cxcr5
Th
c
Batch query
1
2
Wasserstein dist
Mean expression
d
Batch query
1
2
stat

Supplementary Fig. 14 | Gene expression consistency on cellular neighbors of aligned slices

In every row the left panel refers to the first set of coronal sections and the right panel refers to the second set of coronal sections. **a.** Top two genes with lowest L1 Wasserstein distance between gene histograms of reference batch (0) and query batches (1 and 2) for cellular neighbors of aligned slices. **b.** Bottom two genes with highest L1 Wasserstein distance between reference and query batches. **c.** L1 Wasserstein distance across all genes vs. mean gene expression, for both query batches 1 and 2. Interestingly, there is no strong dependency of mean expression showing that the gene expression similarity between cellular neighborhoods of aligned slices is consistent. **d.** Distribution of L1 Wasserstein distances for all genes in query batches 1 and 2.

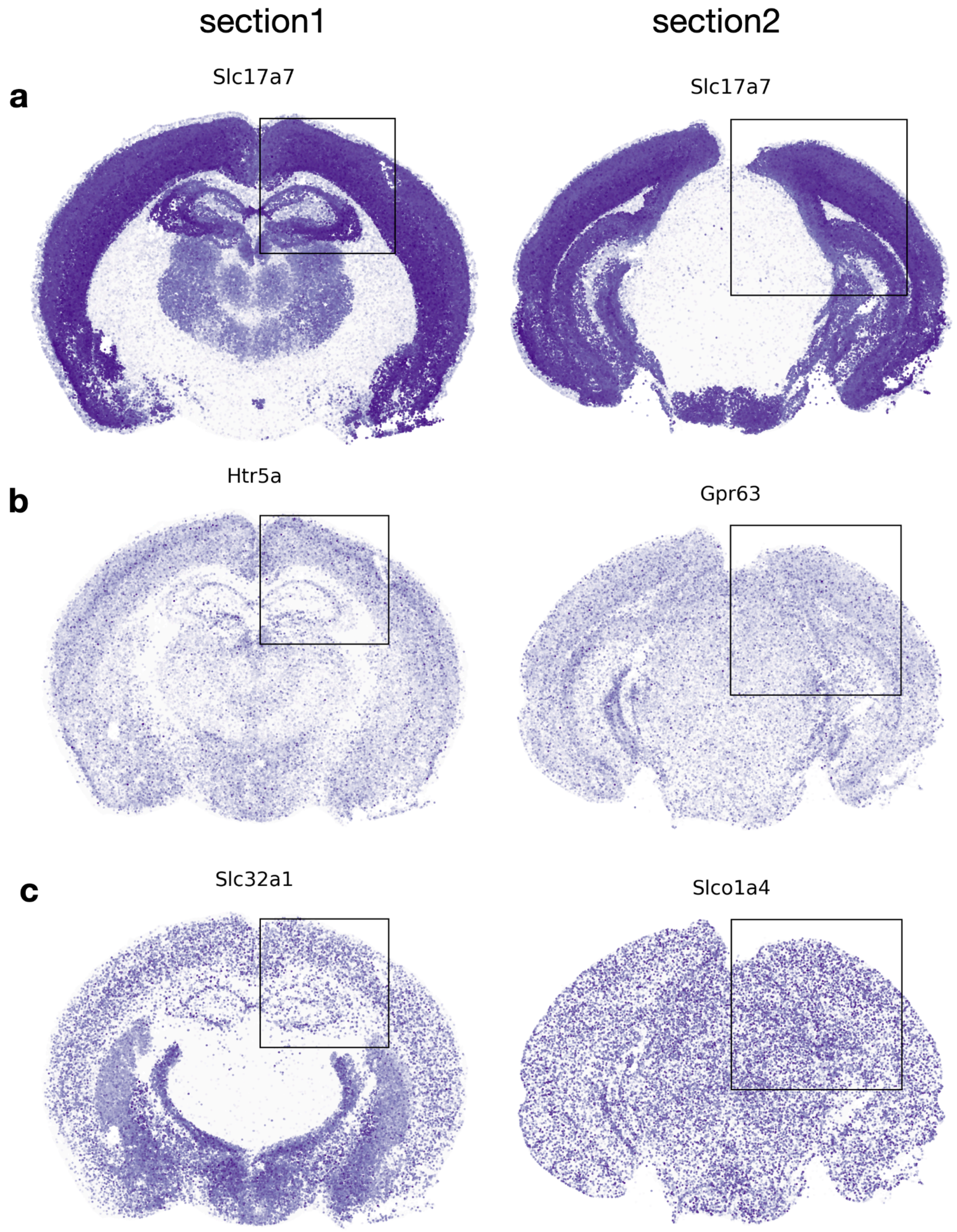

Supplementary Fig. 15 | Gene expression consistency on cellular neighbors of aligned slices

a. Spatial visualization of *Slc17a7.* **b.** Most consistent genes according to consistency analysis (Methods): *Htra5* for brain coronal sections 1 and *Gpr63* for brain coronal sections 2. **c.** Least consistent genes according to consistency analysis: *Slc32a1* for brain coronal sections 1 and *Slco1a4* for brain coronal sections 2*.* Boxes in solid lines correspond to insets in Figure 3.

a

b

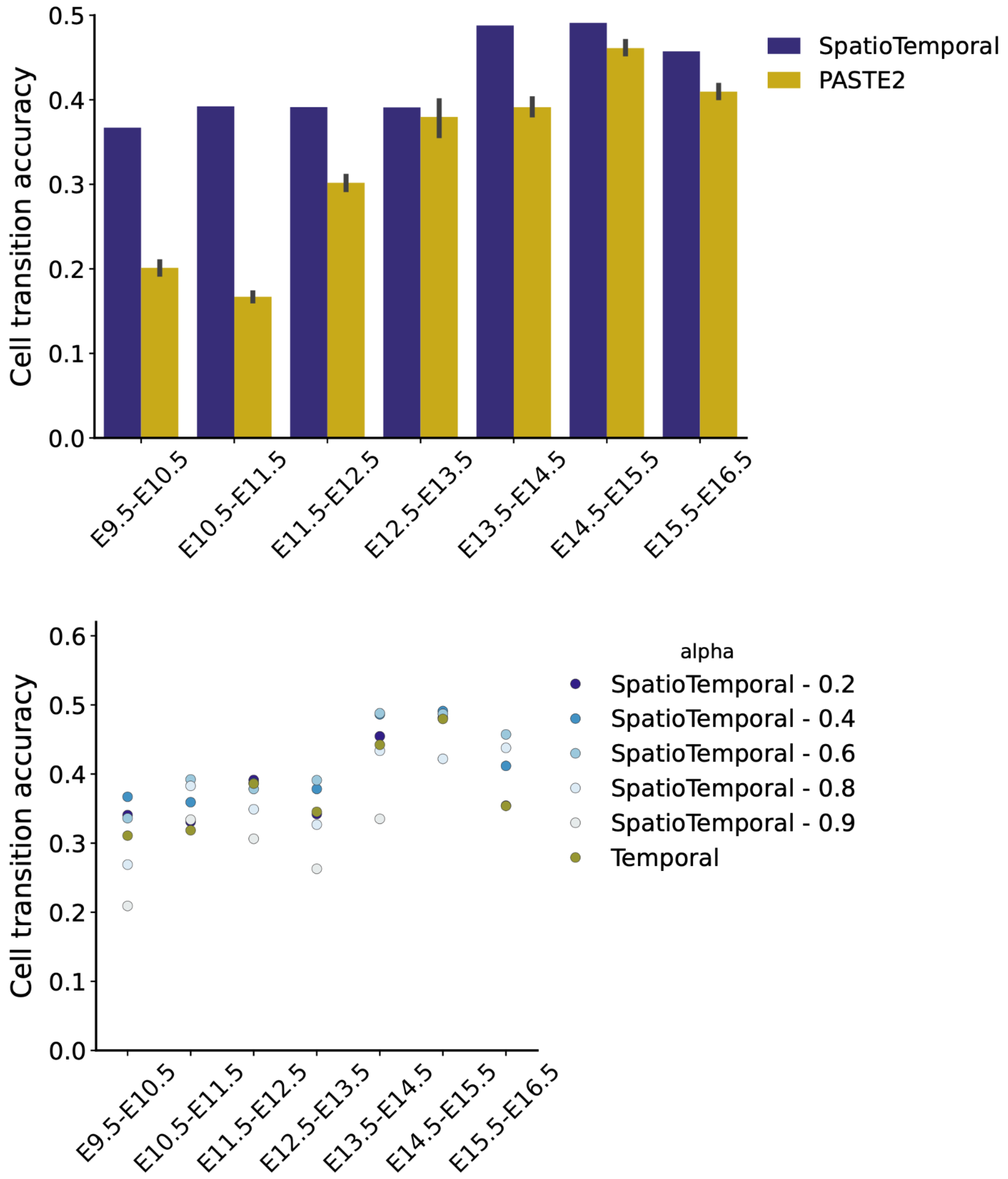

Supplementary Fig. 16 | Accuracy evaluation of moscot.spatiotemporal

Comparison of moscot.spatiotemporal, assessed by the accuracy on curated transitions by developmental stages; **a.** comparison of moscot.spatiotemporal to PASTE2 [10] **(**for PASTE2 we obtain n=10 different subsamples per time point, error bars represent 95% confidence interval) and **b.** the accuracy for different values of the FGW interpolation parameter (Methods and Supplementary Table 5)**.**

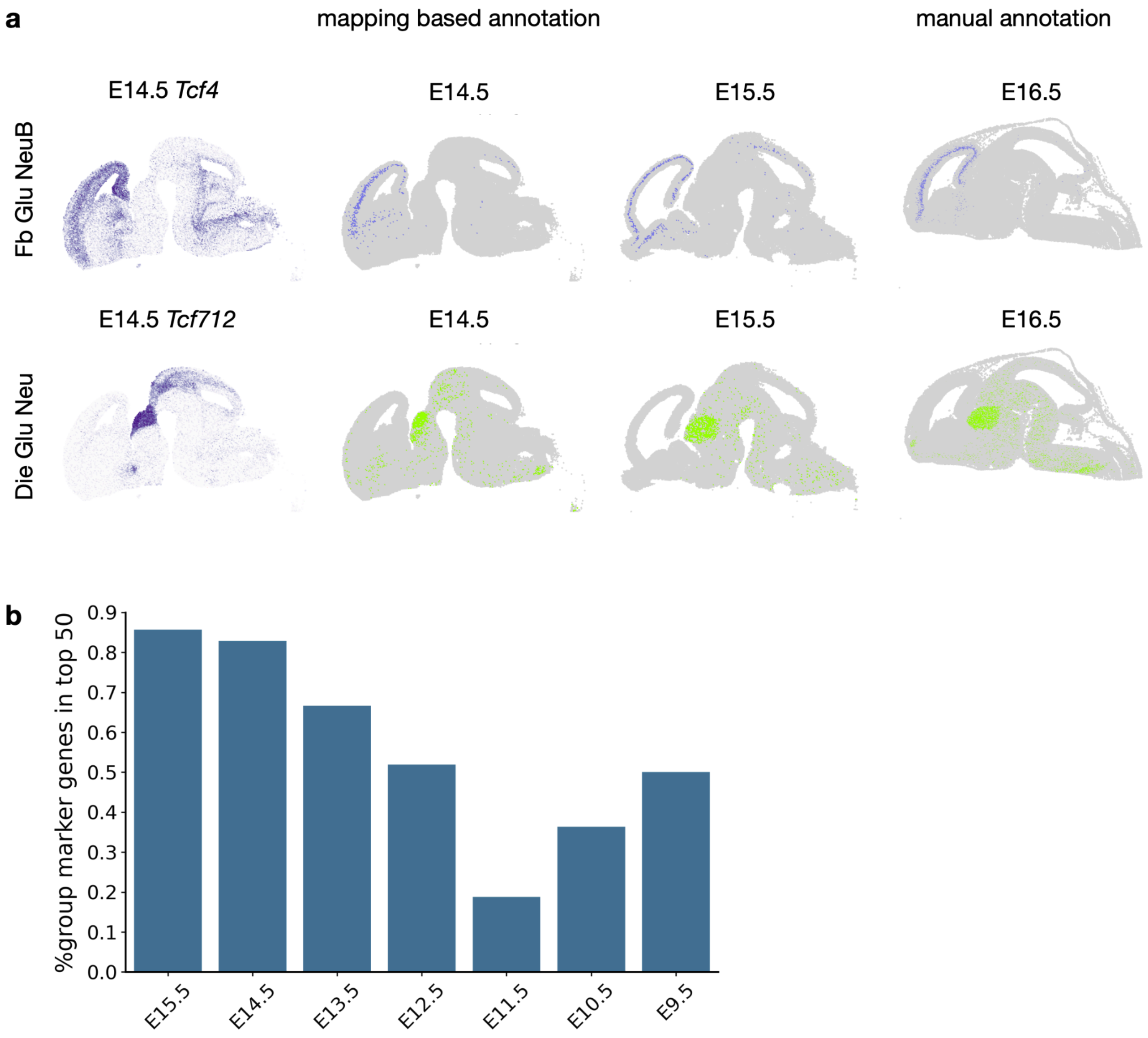

Supplementary Fig. 17 | moscot.spatiotemporal allows for accurate inference of brain cell type annotations

a. Spatial visualization of mapping based annotations of brain cells focusing on specific cell types, Fb Glu NeuB (forebrain glutamatergic neuroblast, top row) and Die Glu Neu (diencephalon glutamatergic neuron, bottom row). Columns, left to right, cell type reported marker gene, cells assigned to cell type at E14.5 and E15.5, manual reference annotation at E16.5. **b.** Bar plot visualizing the percentage of group marker genes found in the top 50 genes associated with the mapping based annotated group (Methods).

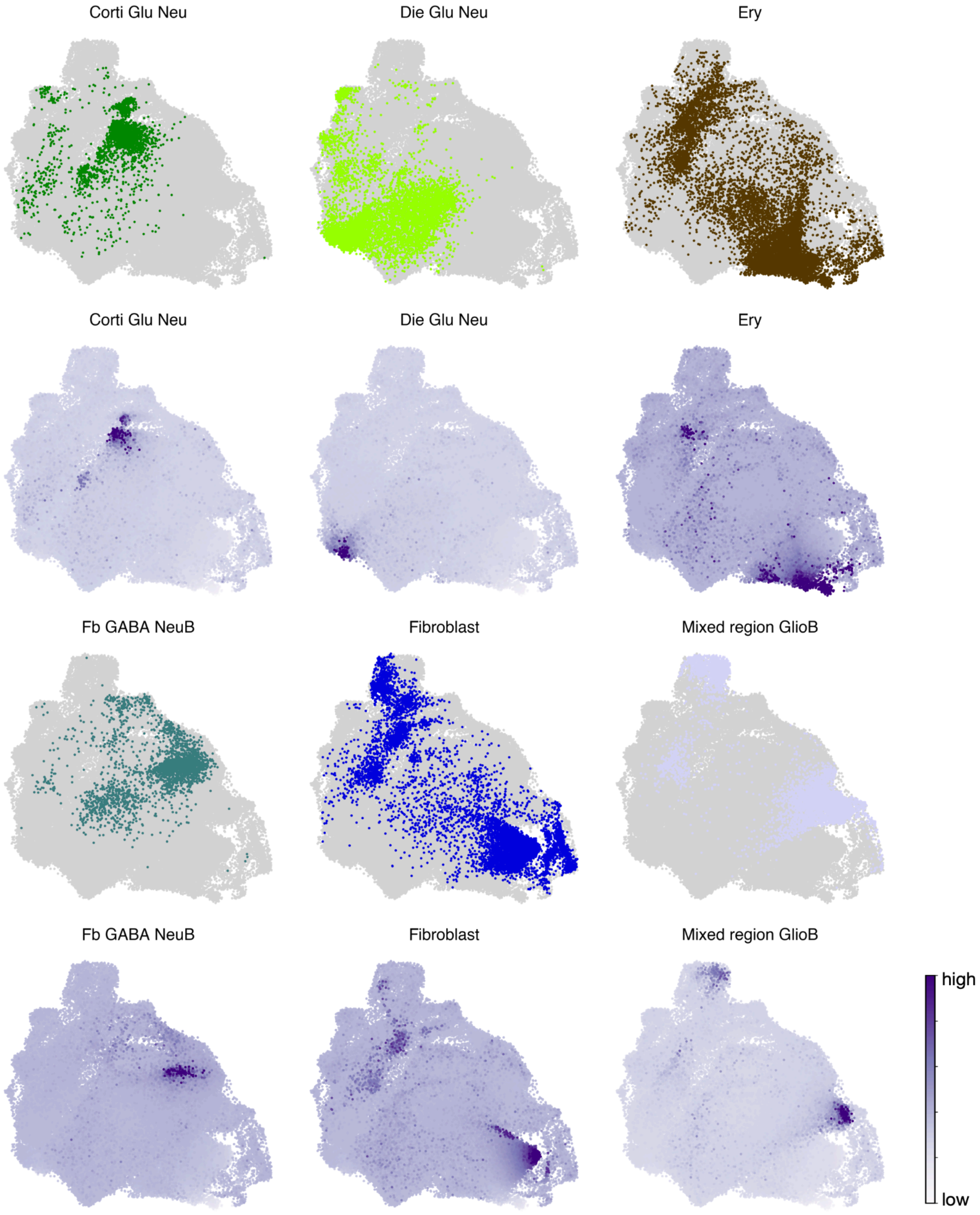

Supplementary Fig. 18 | Terminal states of brain cells as inferred by interfacing moscot.spatiotemporal with CellRank 2

Each subplot provides a visualization for a different terminal state. The UMAPs contain brain cells from E13.5-E16.5 and are colored according to mapping based annotation (first and third row) or CellRank 2 fate probabilities (second and fourth row).

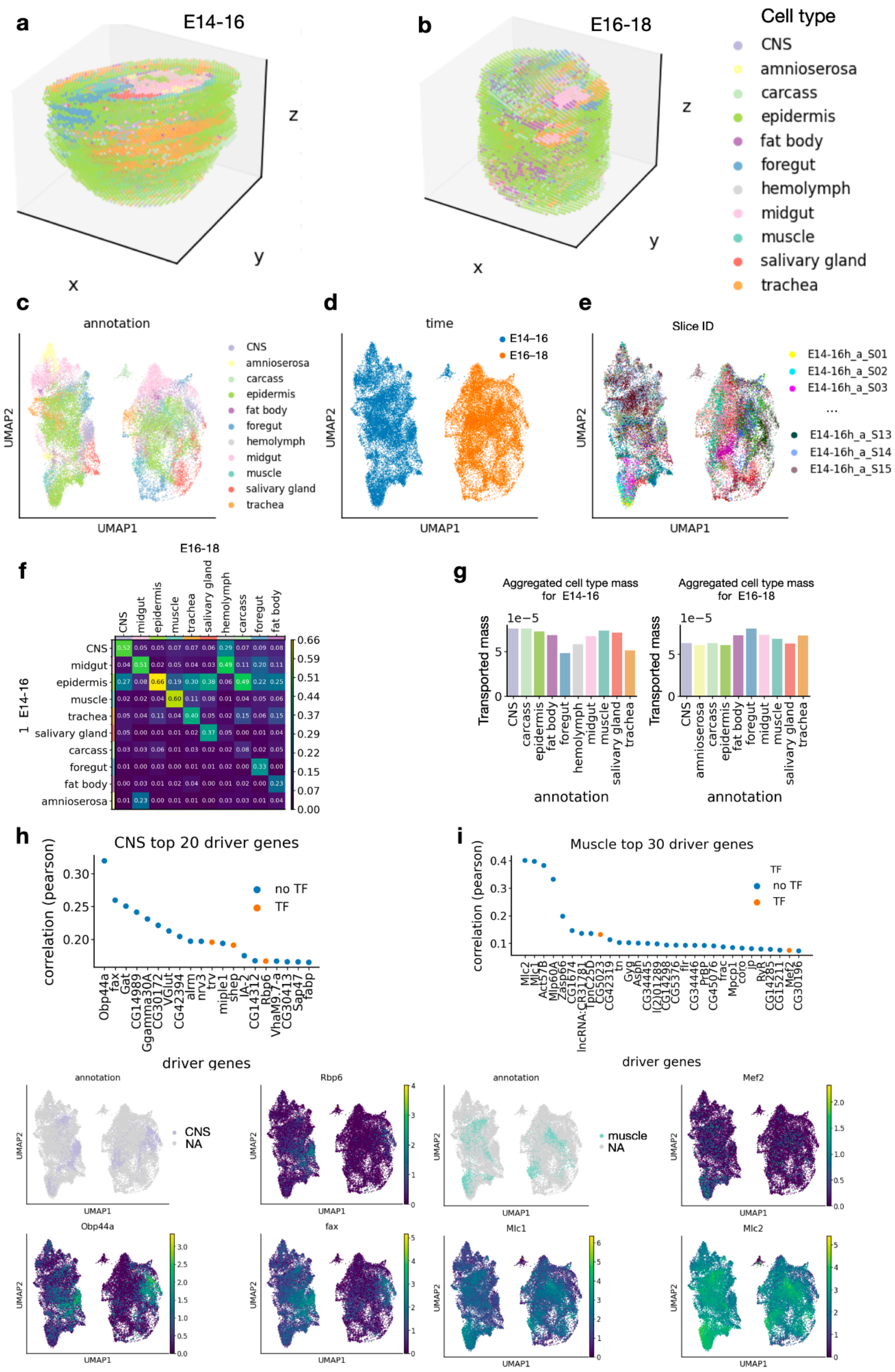
a
E14-16
b
E16-18
x
y
z
Cell type
CNS
amnioserosa
carcass
epidermis
fat body
foregut
hemolymph
midgut
muscle
salivary gland
trachea
c
annotation
d
time
E14–16
E16–18
e
Slice ID
E14-16h_a_S01
E14-16h_a_S02
E14-16h_a_S03
...
E14-16h_a_S13
E14-16h_a_S14
E14-16h_a_S15
UMAP1
UMAP2
f
E16-18
1 E14-16
CNS midgut epidermis muscle trachea salivary gland hemolymph carcass foregut fat body
CNS 0.52 0.05 0.05 0.07 0.07 0.06 0.29 0.07 0.09 0.08
midgut 0.04 0.51 0.02 0.05 0.04 0.03 0.49 0.11 0.20 0.11
epidermis 0.27 0.08 0.66 0.19 0.30 0.38 0.06 0.49 0.22 0.25
muscle 0.02 0.02 0.04 0.60 0.11 0.08 0.01 0.04 0.05 0.06
trachea 0.05 0.04 0.11 0.04 0.40 0.05 0.02 0.15 0.06 0.15
salivary gland 0.05 0.00 0.01 0.01 0.02 0.37 0.05 0.00 0.01 0.04
carcass 0.03 0.03 0.06 0.01 0.03 0.02 0.02 0.08 0.02 0.05
foregut 0.01 0.00 0.03 0.00 0.01 0.01 0.02 0.00 0.33 0.00
fat body 0.00 0.03 0.01 0.02 0.04 0.00 0.01 0.02 0.00 0.23
amnioserosa 0.01 0.23 0.00 0.01 0.01 0.00 0.03 0.03 0.01 0.04
0.66 0.59 0.51 0.44 0.37 0.29 0.22 0.15 0.07 0.00
g
Aggregated cell type mass for E14-16
Aggregated cell type mass for E16-18
Transported mass
1e−5
annotation
h
CNS top 20 driver genes
correlation (pearson)
no TF
TF
Obp44a fax Gat CG14989 Ggamma30A CG30172 VGlut CG42394 alrm nrv3 trv mipel1 shep IA-2 CG14312 Rbp6 VhaM9.7-a CG30413 Sap47 fabp
driver genes
i
Muscle top 30 driver genes
Mlc2 Mlc1 Act57B Mlp60A Zasp66 CG1674 lncRNA:CR31781 TpnC25D CG5023 CG42319 tn Gyg Asph CG34445 l(2)01289 CG14298 CG5376 flr CG34446 PrBP CG45076 frac Mpcp1 coro jp RyR CG14285 CG15211 Mef2 CG30196
annotation
CNS
NA
Rbp6
muscle
Mef2
Obp44a
fax
Mlc1

Supplementary Fig. 19 | moscot.spatiotemporal recovers regulatory mechanisms in the developing drosophila embryo leveraging 3D spatial technologies[11]

a. 3D spatial visualization of cell annotations across various tissue types in Drosophila embryos E14-16, as reported by original Wang et al.[11]. **b.** 3D spatial visualization of cell annotations across various tissue types in Drosophila embryos E16-18, as reported by Wang et al.[11]. **c.** UMAP visualization of cell annotations across various tissue types in Drosophila embryos, as reported by Wang et al.[11]. **d.** UMAP visualization of time points 14 which represents E14-16, and 16, which represents E16-18. **e.** UMAP visualization of slide ID, that is the unique ID source of each stereo-seq slide. **f.** Heatmap displaying the cell transition probabilities from source (E14-16) to target (E16-18). **g.** Bar graphs indicate the mass distribution across various tissues in the pushforward and pullback distribution, highlighting how hemolymph and amnioserosa, present only at E14-16 and E16-18 respectively, have lower mass than other tissues. **h**. Identification of top 20 driver genes for CNS tissue development, with transcription factors (TFs) indicated in orange and other genes in blue. The right side of the panel shows UMAP plots with expression levels of selected genes, including *Rbp6*, *Obp44a*, and *fax*. *Rbp5* and fax were identified also in the original study of Wang et al. leveraging a different algorithm. **i**. Identification of top 30 driver genes for muscle tissue development, with transcription factors (TFs) indicated in orange and other genes in blue. The right side of the panel illustrates UMAP plots showing expression levels for *Mef2*, *Mlc1*, and *Mlc2*. *Mef2* is also reported by Wang et al., and it was identified leveraging a different algorithm.

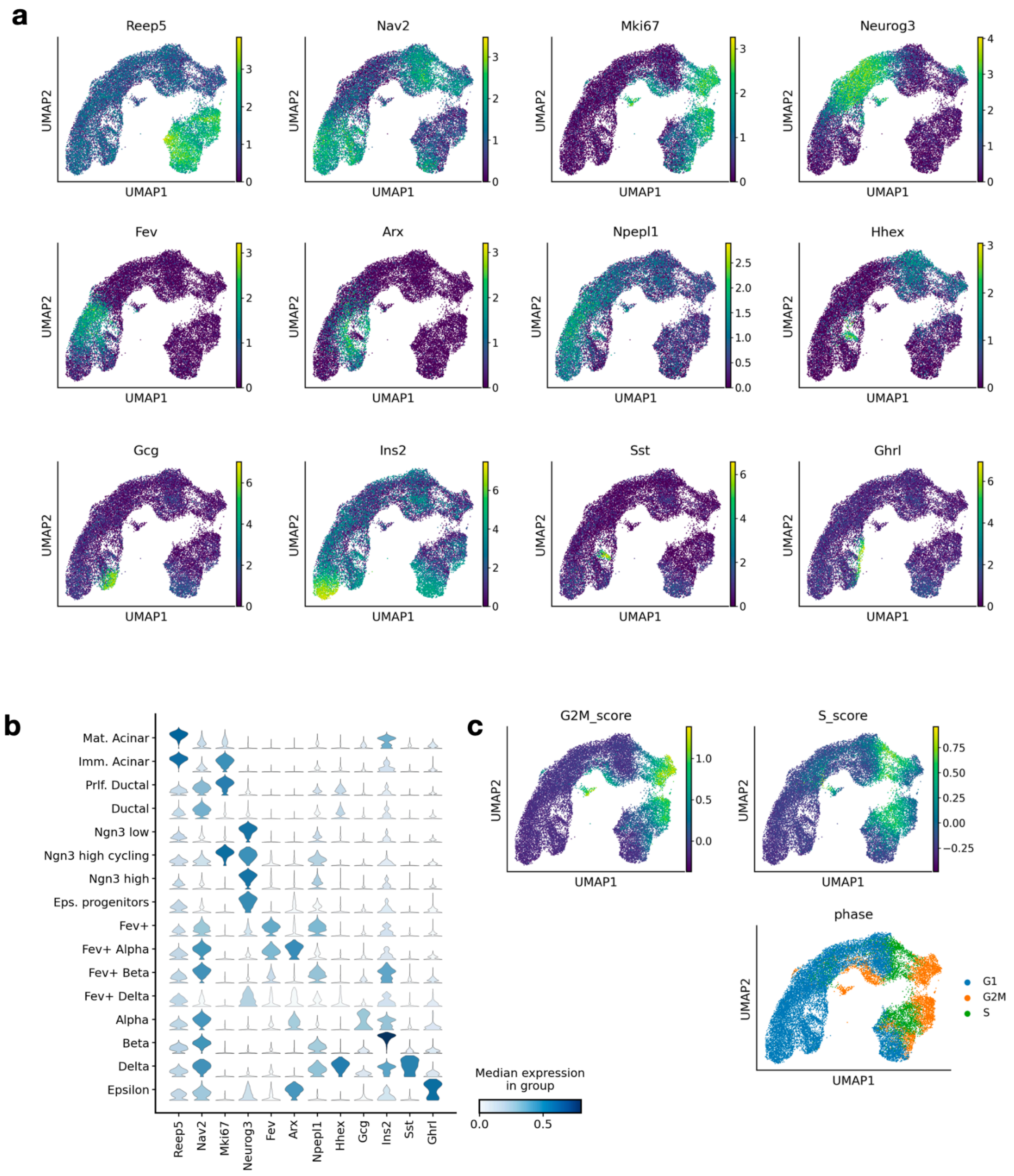

Supplementary Fig. 20 | Marker gene expression in pancreatic endocrinogenesis

a. Processed gene expression (processed with *scanpy.pp.normalize_total* and *scanpy.pp.log1p*) of selected marker genes. In particular, *Reep5* is a marker for acinar cells, *Nav2* for ductal cells, *Mki67* for proliferative ductal cells, *Neurog3* for Ngn3^{low} and Ngn3^{high} cells, *Fev* for *Fev*+ cells,

Arx for *Fev*+ alpha cells, *Npepl1* for *Fev*+ beta cells, *Hhex* for *Fev*+ delta cells (and delta cells), *Gcg* for alpha cells, *Ins2* for beta cells, *Sst* for delta cells, and *Ghrl* for epsilon cells. **b.** Min-max normalized processed gene expression per cell type. **c.** G2M score, S score and proliferation phase as computed by scanpy's *score_genes_cell_cycle* function.

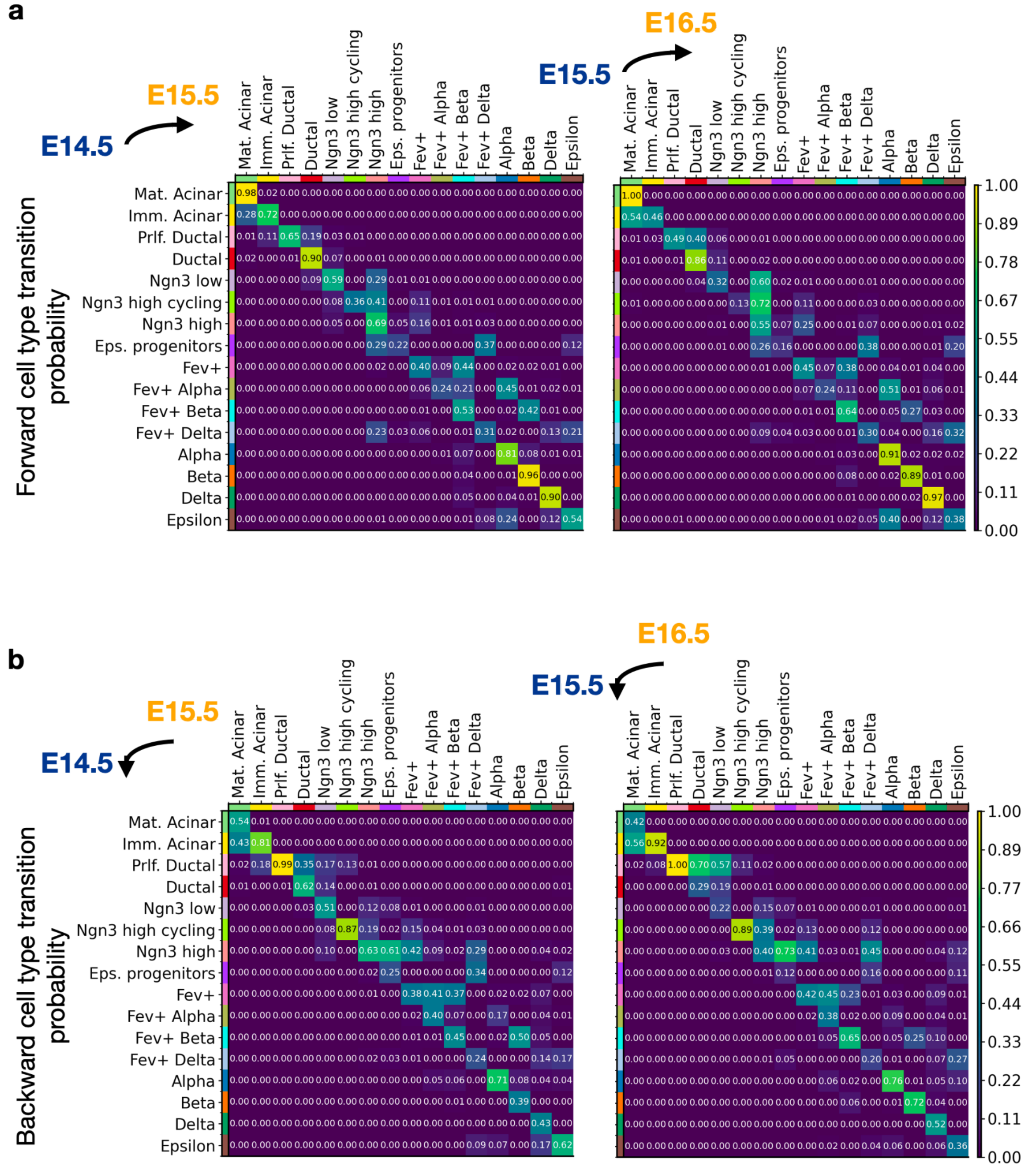

Supplementary Fig. 21 | Cell type transition probabilities of the full pancreatic endocrinogenesis dataset

a. Transition probabilities obtained by moscot.time and aggregated per cell type. Each row sums up to 1, hence each entry (i,j) denotes the probability of cell type i in E14.5 to transition to cell

type j in E15.5. **b.** Transition probabilities obtained by moscot.time, but this time each column adds up to 1, hence each entry (i,j) denotes the probability of cell type i in E14.5 to be an ancestor of cell type j in E15.5.

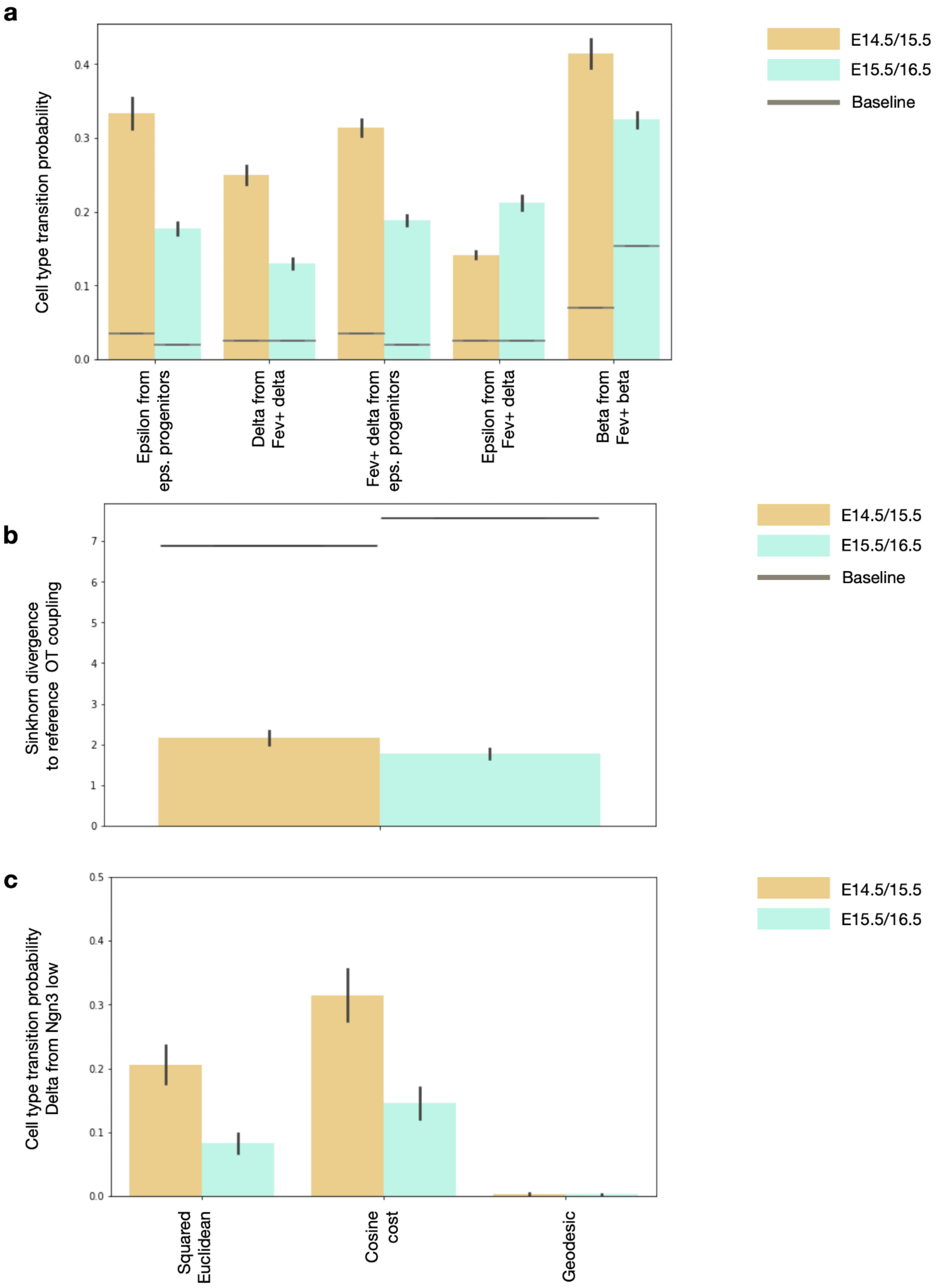
a
Cell type transition probability
0.0
0.1
0.2
0.3
0.4
Epsilon from eps. progenitors
Delta from Fev+ delta
Fev+ delta from eps. progenitors
Epsilon from Fev+ delta
Beta from Fev+ beta
E14.5/15.5
E15.5/16.5
Baseline
b
Sinkhorn divergence to reference OT coupling
0
1
2
3
4
5
6
7
E14.5/15.5
E15.5/16.5
Baseline
c
Cell type transition probability Delta from Ngn3 low
0.0
0.1
0.2
0.3
0.4
0.5
Squared Euclidean
Cosine cost
Geodesic
E14.5/15.5
E15.5/16.5

Supplementary Fig. 22 | Stability of coupling of moscot.time with respect to different embeddings, costs and parameters of the cost

Different statistics derived from the cell type-aggregated transport matrix across different configurations of the embedding, cost, and hyperparameters of the cost (Methods), mean and standard error reported.
a. Selected cell type transitions of moscot run with different configurations for the E14.5/15.5 coupling and the E15.5/16.5 coupling. The baseline corresponds to the independent coupling. The higher the cell type transition probabilities, the more signal the coupling captures (n=63 configurations for each pair of time points). **b.** Sinkhorn divergence between the aggregated transport matrix of the reference configuration (used in the analysis, Methods) and the aggregated transport matrix obtained from different configurations of the embedding, cost, and parameters of the cost. The baseline is the Sinkhorn divergence between the reference configuration and the outer coupling. The lower the Sinkhorn divergence, the more similar to the reference coupling (n=63 configurations for each pair of time points). **c.** Proportion of delta cells which are predicted to be derived directly from Ngn3 low for different embeddings, costs and specifications of the cost (Methods, Sq. Eucl. cost: n=9 data points, cosine cost: n=9, geodesic cost: n=45 for each pair of time points). As a direct transition from Ngn3 low to delta is very unlikely, the lower the score, the better. In all plots, mean and standard error are reported.

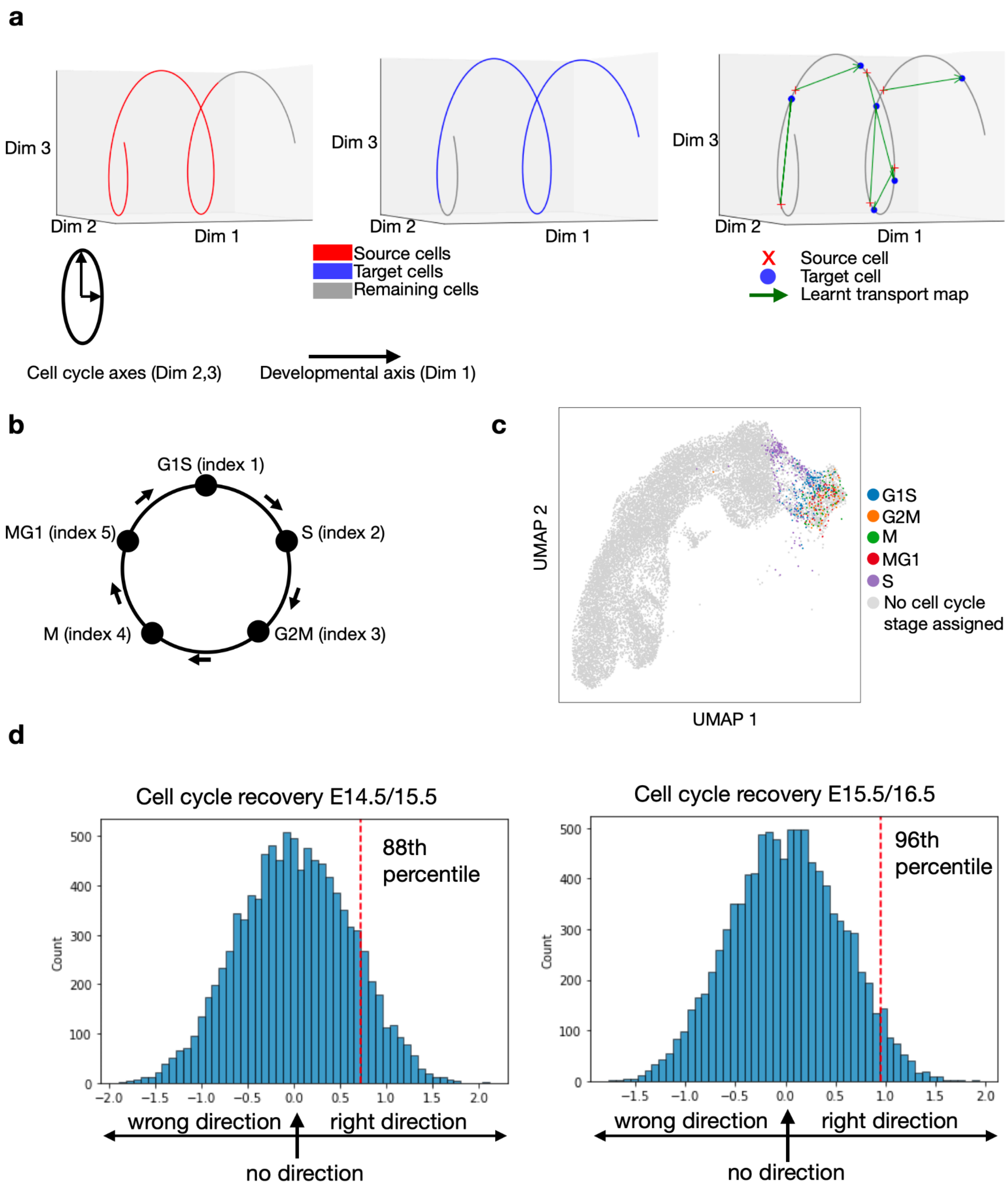

Supplementary Fig. 23 | Moscot can recover cell cycles

a. Implementation of the sketch used for explaining transcriptomic dynamics during cell cycles in Schwabe et al.[12] as toy data example for cell cycle dynamics. Left: source distribution corresponding to an earlier time point. Middle: target distribution corresponding to a later time point. Right: Samples from the learnt transport map to model the trajectory of cells. **b.** Sketch of

the ground truth biological cell cycle with indices used in the explanation. **c.** UMAP of the pancreatic endocrinogenesis dataset (E14.5, 15.5, 16.5) colored by assigned cell cycle stage. **d.** Permutation test scores for assessing the correctness of the directionality of the cell cycle computed with moscot.time for time points E14.5/15.5 (left) and time points E15.5/16.5 (right).

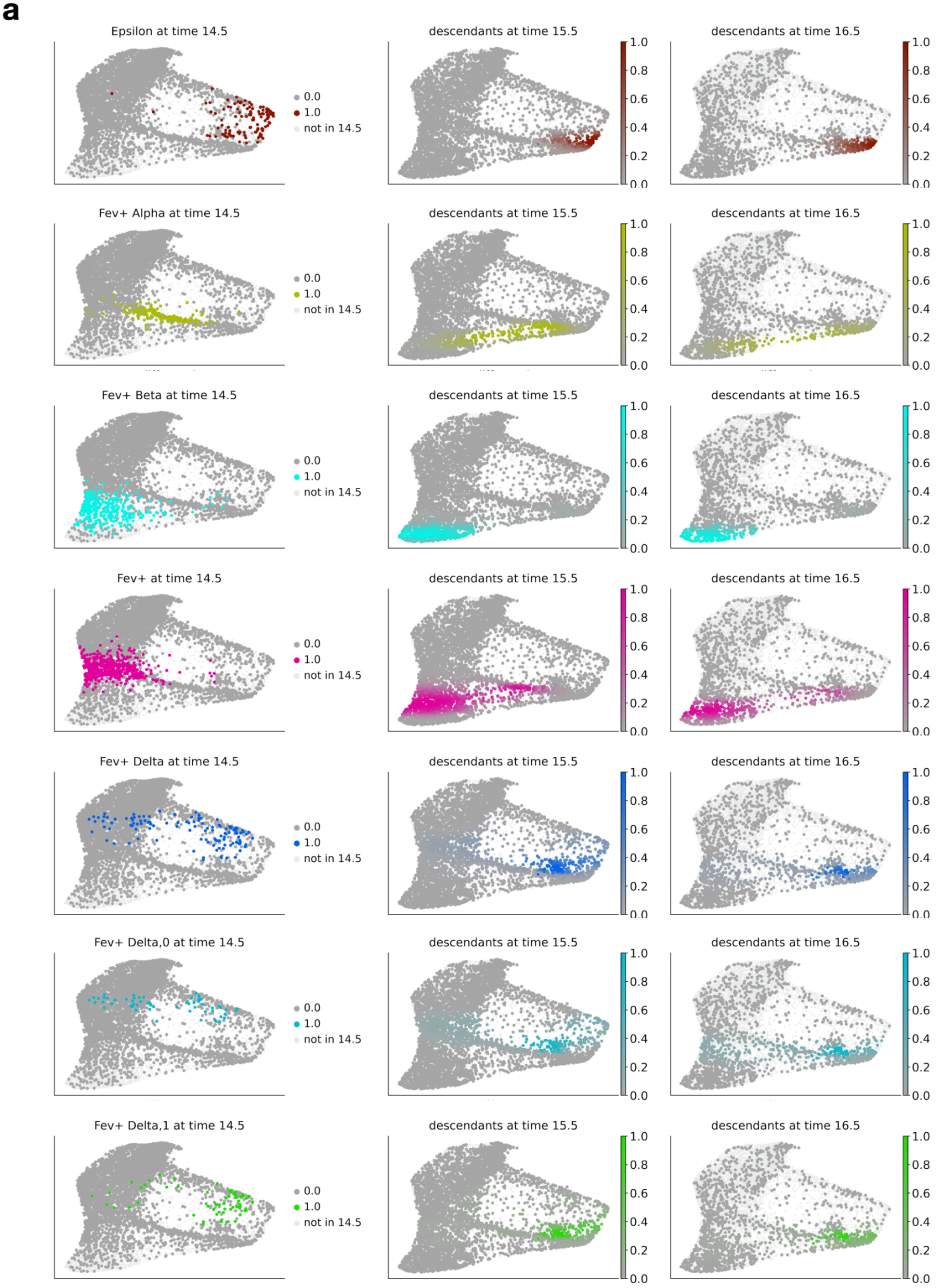
a
Epsilon at time 14.5
descendants at time 15.5
descendants at time 16.5
0.0
1.0
not in 14.5
Fev+ Alpha at time 14.5
Fev+ Beta at time 14.5
Fev+ at time 14.5
Fev+ Delta at time 14.5
Fev+ Delta,0 at time 14.5
Fev+ Delta,1 at time 14.5
0.2
0.4
0.6
0.8

Supplementary Fig. 24 | Descendants of endocrine and endocrine progenitors on a diffusion map

a. Cell types at E14.5 and their respective ancestors at E15.5 and E16.5 computed by moscot.time, and visualized on a PHATE embedding.

a

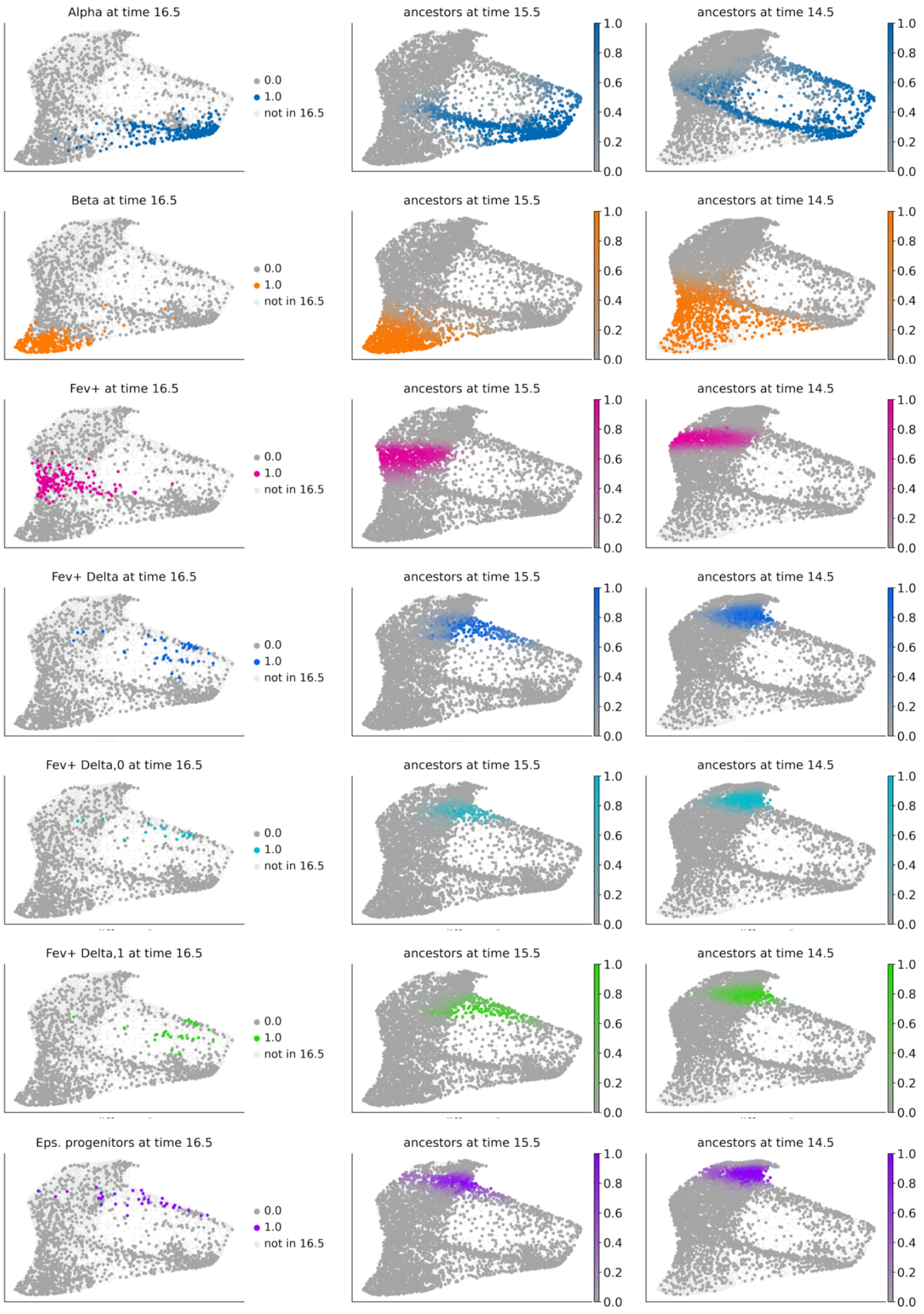

Alpha at time 16.5
ancestors at time 15.5
ancestors at time 14.5
Beta at time 16.5
Fev+ at time 16.5
Fev+ Delta at time 16.5
Fev+ Delta,0 at time 16.5
Fev+ Delta,1 at time 16.5
Eps. progenitors at time 16.5
0.0
1.0
not in 16.5

Supplementary Fig. 25 | Ancestry of endocrine and endocrine progenitors on a diffusion map.

a. Cell types at E16.5 and their respective ancestors at E15.5 and E14.5 computed by moscot.time, and visualized on a PHATE embedding.

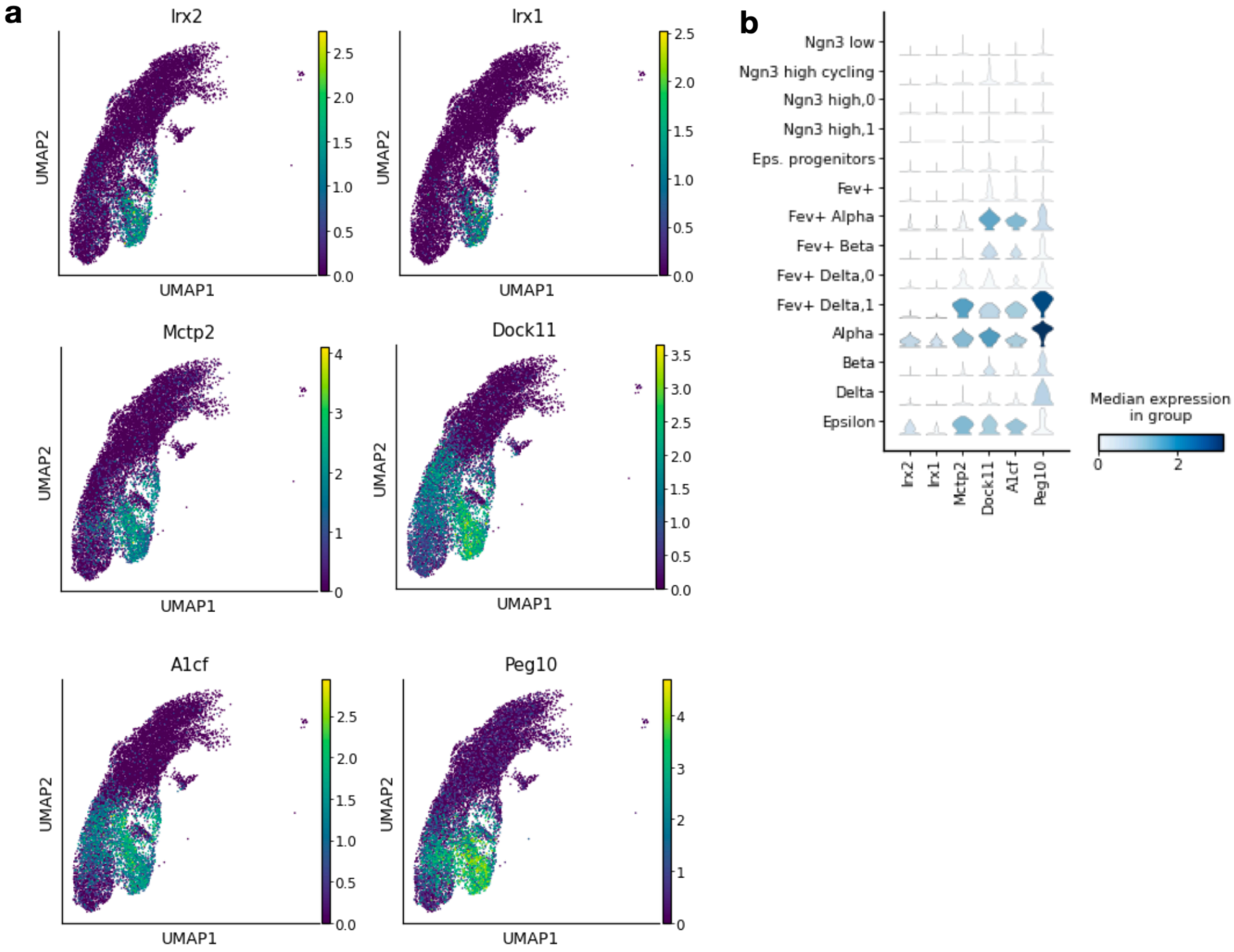

Supplementary Fig. 26 | Marker genes for the transition from epsilon cells to alpha cells

a. By leveraging moscot's capability of identifying marker features (Methods) we identify genes which are highly expressed in epsilon cells that are likely to transition towards an alpha cell state. *Irx2* has been reported as a key TF for these cell states[13], while *Irx1* was reported for the analogue cell states in human pancreatic endocrinogenesis[14]. *Peg10* has been reported as a driver gene for alpha cells[15], while *Mctp2, Dock11,* and *A1cf* have not been reported in this context before. **b.** Processed gene expression of considered genes per cell type.

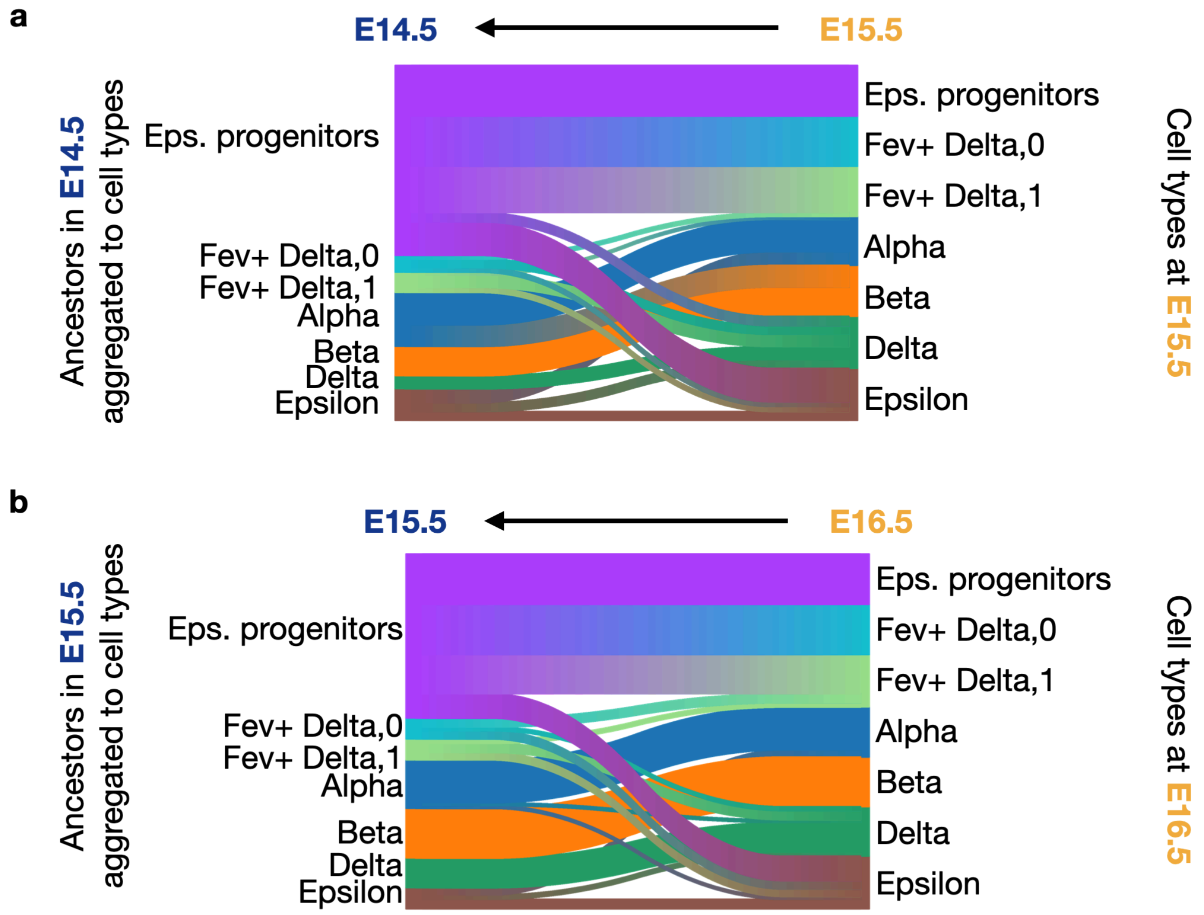

Supplementary Fig. 27 | Ancestors of refined cell types obtained by moscot.time

a. Cells at E15.5 aggregated to cell types and their ancestors in E14.5 aggregated to cell types and **b**. cells at E16.5 aggregated to cell types and their ancestors in E15.5 aggregated to cell types. Only ancestries with a probability of at least 0.05 are visualized. While slight differences in transition probabilities are likely due to noise in the cell type annotation, sequencing biases, or limitations of the moscot algorithm, certain changes in transition likelihoods might be biological. While we could not find any further evidence for the non-zero transdifferentiation probability between alpha and beta cells in E14.5/E15.5, which ceases for E15.5/E16.5, this cellular behavior has been observed in mice in the late stages of pancreas development[16].

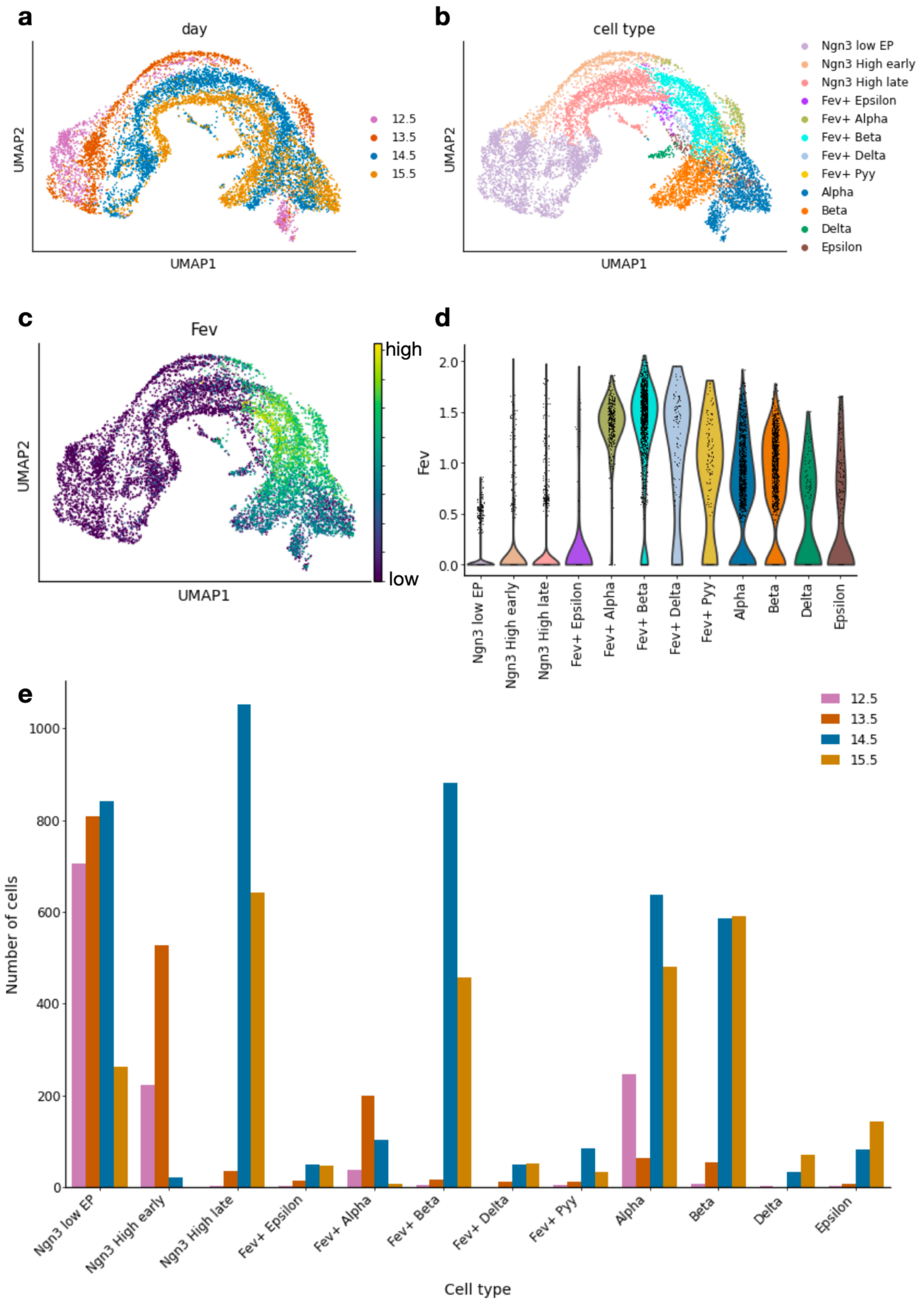
a
day
12.5
13.5
14.5
15.5
UMAP1
UMAP2
b
cell type
Ngn3 low EP
Ngn3 High early
Ngn3 High late
Fev+ Epsilon
Fev+ Alpha
Fev+ Beta
Fev+ Delta
Fev+ Pyy
Alpha
Beta
Delta
Epsilon
UMAP1
UMAP2
c
Fev
high
low
UMAP1
UMAP2
d
Fev
0.0
0.5
1.0
1.5
2.0
e
Number of cells
0
200
400
600
800
1000
Cell type

Supplementary Fig. 28 | Overview of the pancreatic endocrinogenesis dataset published by Bastidas-Ponce et al.[17]

a. UMAP embedding colored by cell type of the pancreatic endocrinogenesis dataset published by Bastidas-Ponce et al.[17]. The data was subset to endocrine cells and their progenitors. Subsequently, the data was preprocessed by normalization, log1p-transformation and PCA computation before calculation of the neighborhood graph, based on which the UMAP was computed. **b.** UMAP embedding colored by cell type. **c.** Processed gene expression of *Fev* on the UMAP, visually suggesting there is no expression of *Fev* in the population annotated as *Fev*+ epsilon. **d.** Quantitative confirmation that there is barely any expression of *Fev* in the cell type annotated as *Fev*+ epsilon in Bastidas-Ponce et al.

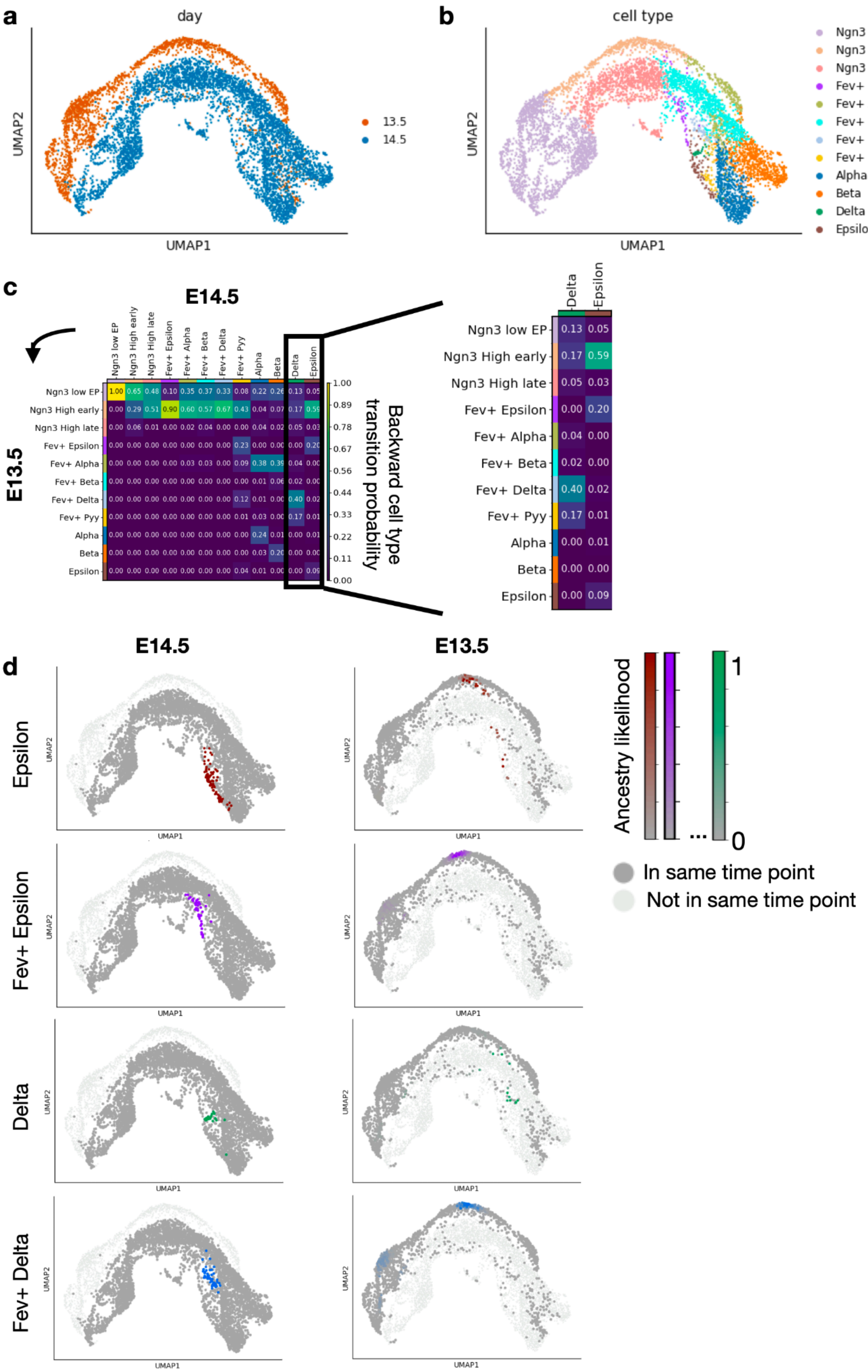
a
day
UMAP2
UMAP1
13.5
14.5
b
cell type
UMAP2
UMAP1
Ngn3 low EP
Ngn3 High early
Ngn3 High late
Fev+ Epsilon
Fev+ Alpha
Fev+ Beta
Fev+ Delta
Fev+ Pyy
Alpha
Beta
Delta
Epsilon
c
E14.5
E13.5
Backward cell type transition probability
Delta
Epsilon
Ngn3 low EP 0.13 0.05
Ngn3 High early 0.17 0.59
Ngn3 High late 0.05 0.03
Fev+ Epsilon 0.00 0.20
Fev+ Alpha 0.04 0.00
Fev+ Beta 0.02 0.00
Fev+ Delta 0.40 0.02
Fev+ Pyy 0.17 0.01
Alpha 0.00 0.01
Beta 0.00 0.00
Epsilon 0.00 0.09
d
E14.5
E13.5
Epsilon
Fev+ Epsilon
Delta
Fev+ Delta
UMAP1
UMAP2
Ancestry likelihood
1
0
In same time point
Not in same time point

Supplementary Fig. 29 | Moscot analysis of E13.5 and E14.5 of pancreatic endocrinogenesis dataset is consistent with the analysis on the novel multiome dataset.

a. UMAP embedding colored by cell type of the pancreatic endocrinogenesis dataset published by Bastidas-Ponce et al.[17] filtered to embryonic day 13.5 and 14.5. The data was subset to endocrine cells and their progenitors. **b.** UMAP embedding colored by cell type annotated as provided by the authors. The *Fev*+ epsilon population is similar to the epsilon progenitor population defined in this work, see Supplementary Fig. 28 . **c.** Probability of a cell type in time point E14.5 to originate from a cell type in E13.5 calculated by moscot. Highlighted are the predicted cell type origins of delta and epsilon cells **d.** Visualization of ancestry likelihood of single cells predicted by moscot for epsilon, *Fev*+ epsilon, delta, and *Fev*+ delta cells.

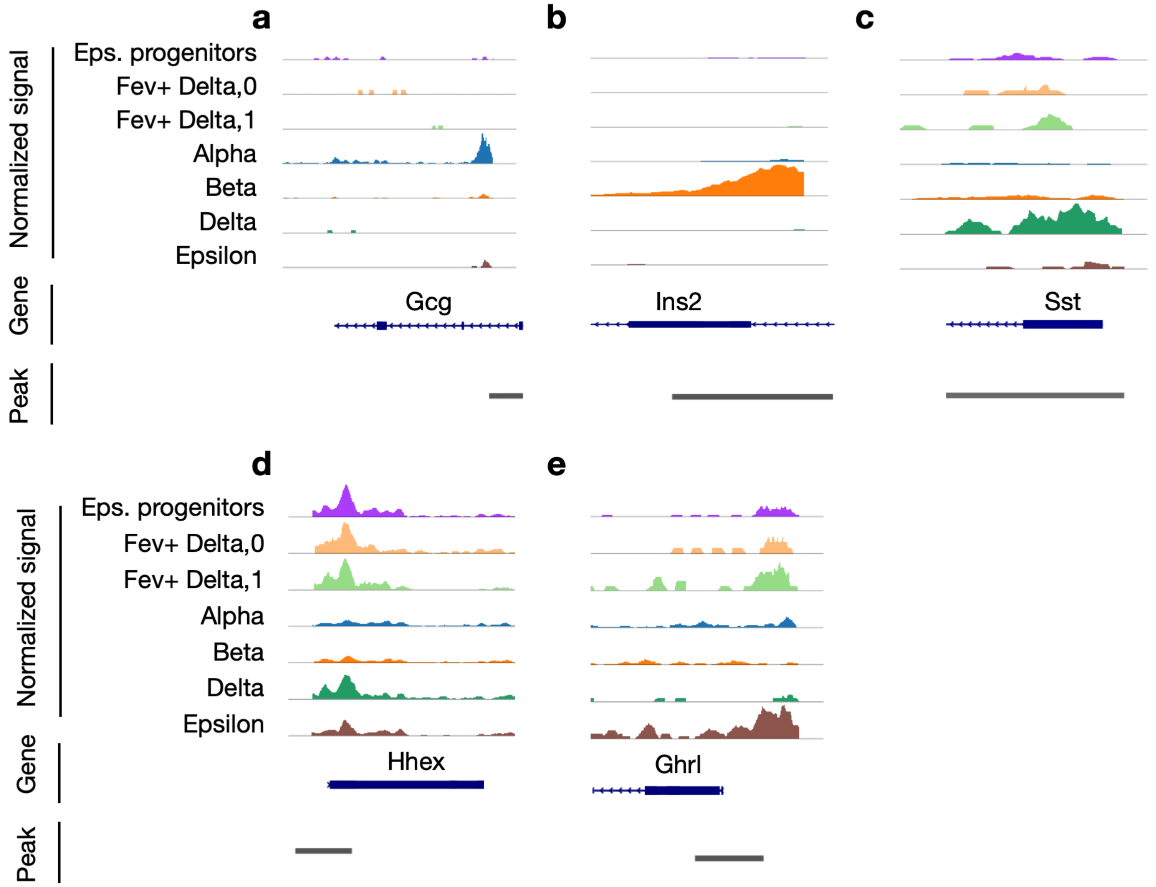

Supplementary Fig. 30 | Chromatin accessibility at the promoter regions of *Gcg* and *Ins2*

Open chromatin around the promoter region of **a.** *Gcg* (peak 2-62474530-62483650), **b.** *Ins2* (7-142678656-142679685), **c.** *Sst* (16-23889573-23890958), **d.** *Hhex* (19-37434810-37440731) **e.** *Ghrl* (6-113716119-113719880).

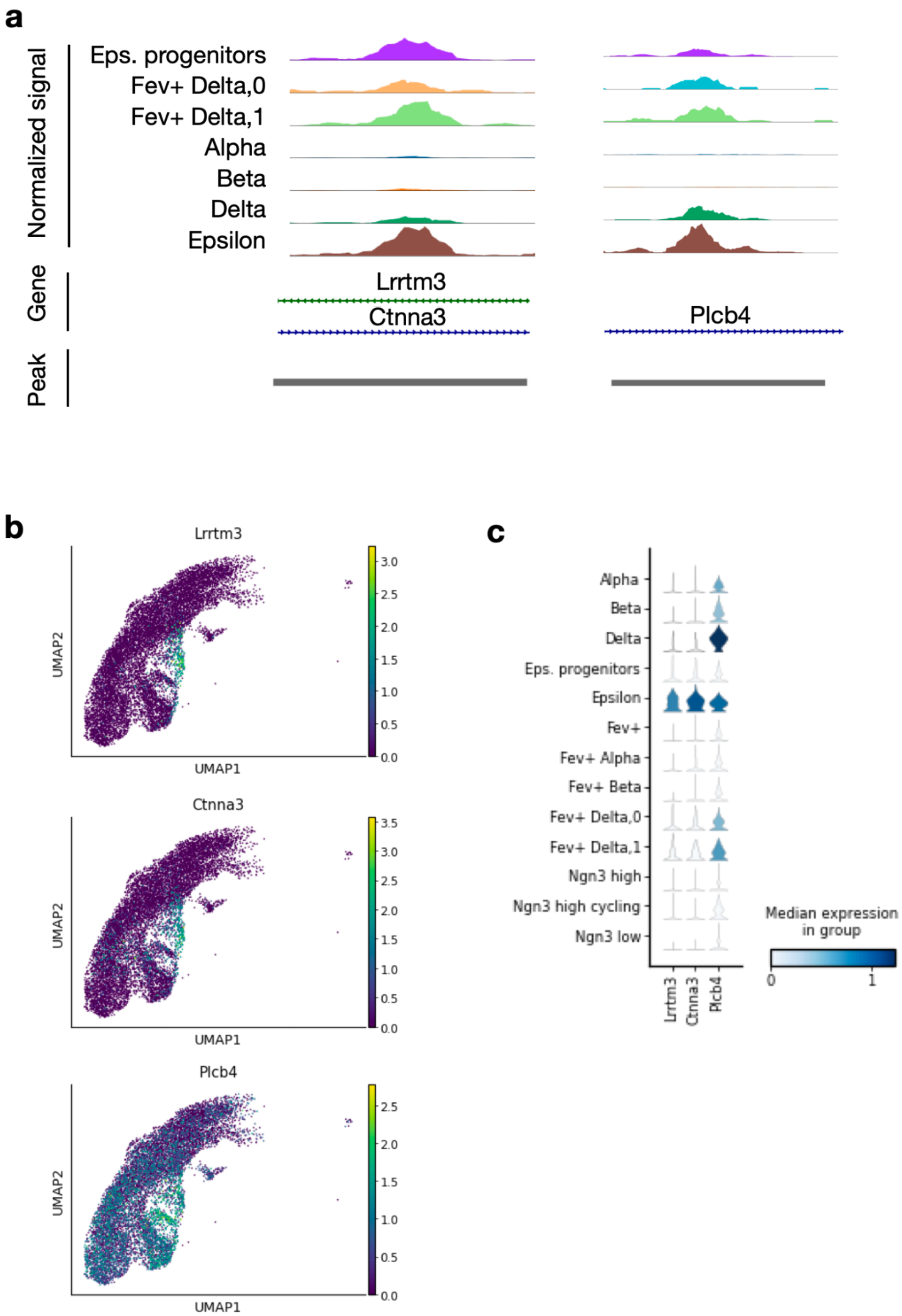

Supplementary Fig. 31 | Expression of genes most associated with the marker peaks

a. Open chromatin accessibility for peak 10-64082075-64082998, the most significant peak for the epsilon progenitor cell cluster (Supplementary Table 11, Supplementary Note 7), and for peak 2-135719090-135720017, the most significant peak for the *Fev*+ delta cell population (Supplementary Table 12). **b.** Left: Processed (normalized and log1p-transformed) gene expression of *Lrrtm3*, the gene most highly correlated with the most significantly accessible

peak for the epsilon progenitor population). Middle: Processed gene expression of *Ctnna3*, the second highly correlated gene with the marker peak of the epsilon progenitor population. Right: Processed gene expression of *Plcb4*, the gene most highly correlated with the most significant peak of the *Fev*+ delta cell population. **c.** Processed gene expression of the considered genes per cell type.

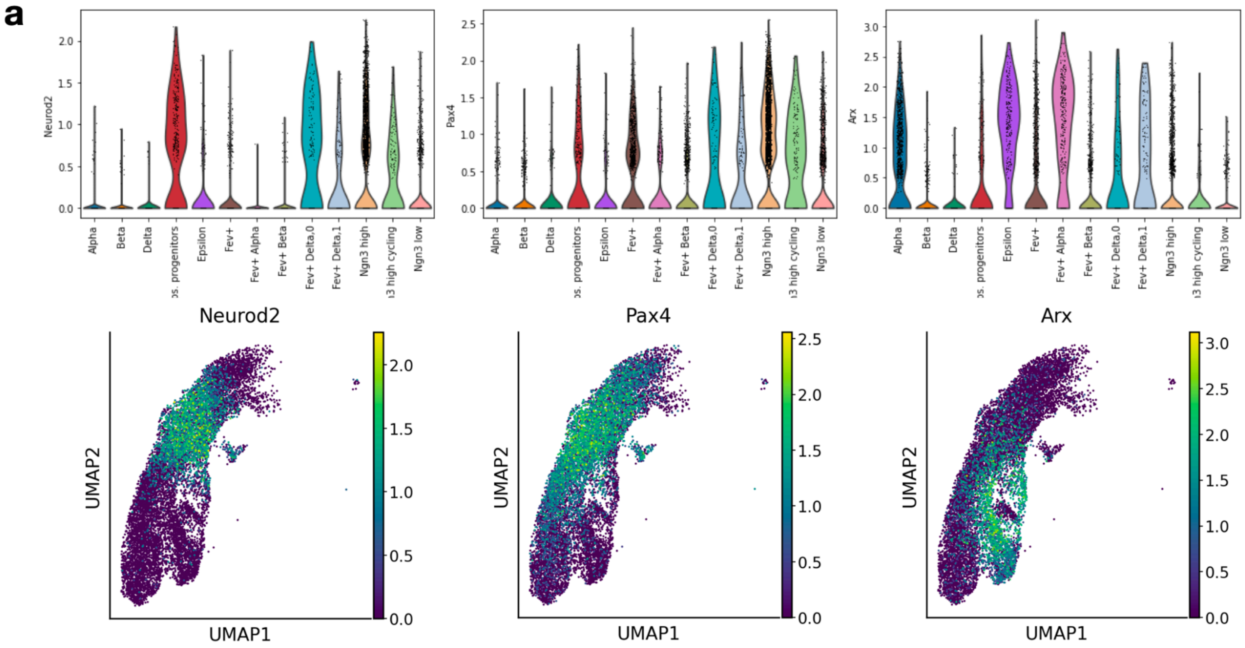

Supplementary Fig. 32 | Quantification of marker genes in the *Fev*+ delta population

a. Expression per cell type (top) and per cell (bottom) of *Neurod2*, *Pax4 (*beta cell activator*)* and *Arx* (alpha cell activator).

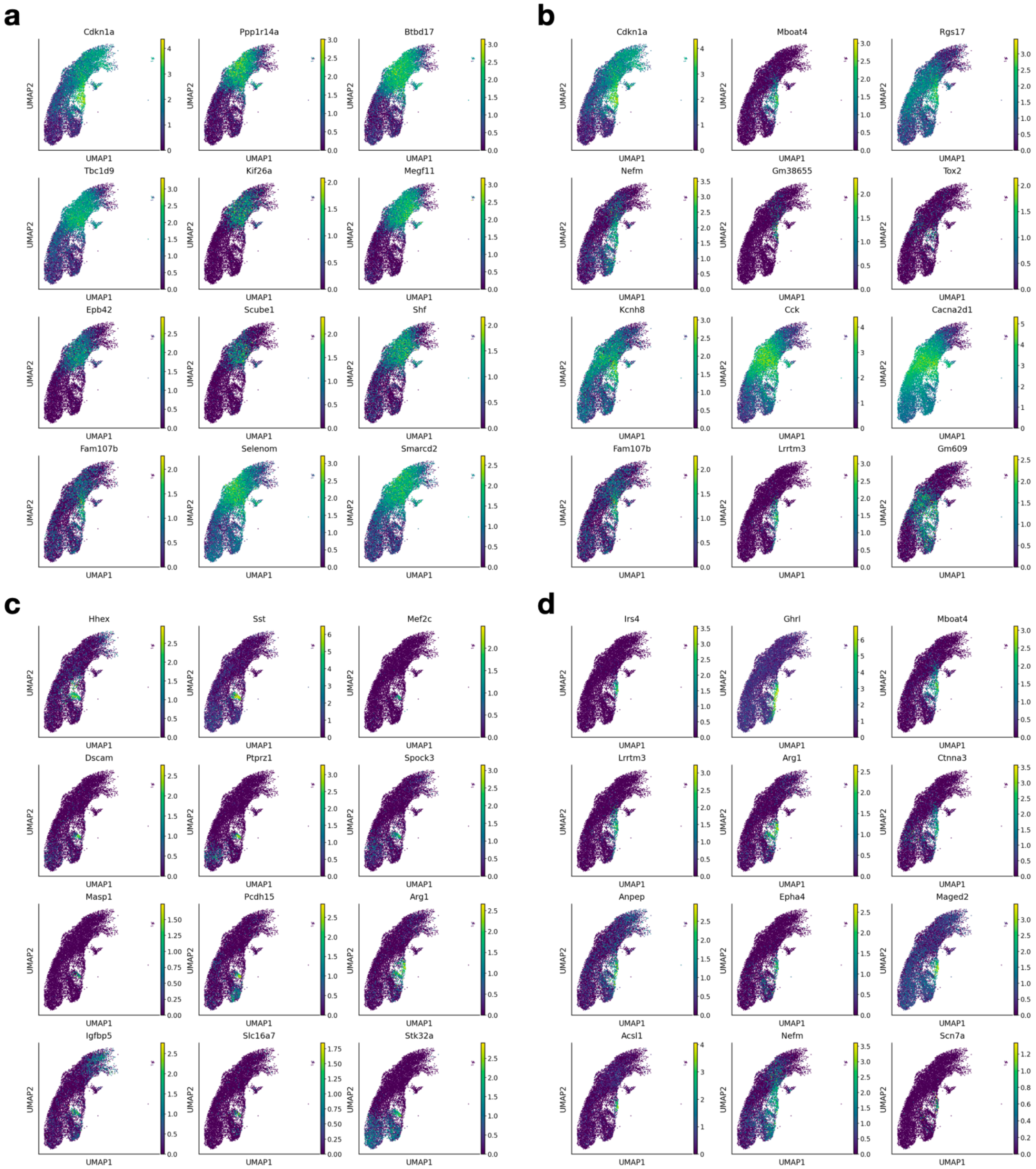

Supplementary Fig. 33 | Driver genes computed by moscot.time for delta/epsilon lineage

a. Driver genes for epsilon progenitors as computed by moscot.time (Methods). While a few markers have been reported (*Ppp1r14a*[17], *Megf11*[24], *Selenom*[25], *Smarcd2*[17]), or were considered in different contexts (e.g. the cell cycle inhibitor *Cdkn1a*[26], *Rgs17*[27], *Cacna2d1*[28]), we report genes which have been less studied in the context of pancreatic endocrinogenesis (*Btbd17*, *Tbc1d9*, *Kif26a*, *Epb42, Scube1, Shf, Fam107b, Kcnh8, Lrrtm3* (Supplementary Fig. 31),

Tmem184c, Cer1) (Supplementary Table 15) **b.** Driver genes for the *Fev*+ delta population (*Fev*+ delta-0 and *Fev*+ delta-1 combined due to their high similarity in gene expression and ATAC profile). While we recover genes which we also report for epsilon progenitors, there are genes which have not been reported in this context, such as *Mboat4* [29], *Rgs17*[27], *Kcnh8* [30], *Cck* [31], *Cacna2d1* [32], *Lrrtm3* [33] (Supplementary Fig. 31) and less studied, but significantly expressed genes (*Nefm, Gm38655, Tox2, Gm609)* (Supplementary Table 16). **c.** Driver genes for delta cells. While *Hhex* and *Sst* confirm the reliability of moscot's marker gene recovery method as the most well-known marker genes for delta cells (also *Spock3*[34], *Mef2c*[1317]), we also report new genes in this context: *Dscam, Ptprz1, Masp1, Pcdh15, Igfbp5, Slc16a7, Stk32a*. Moreover, *Arg1* is recovered due to the high plasiticy of *Fev*+ delta cells, and other genes shared with markers from *Fev*+ delta cells or epsilon progenitor cells are recovered (Supplementary Table 17). **d.** Driver genes for the epsilon population. Besides already considered genes we report the well-known markers *Irs4*[34] and *Ghrl*[35], as well as *Ctnna3* (Supplementary Fig. 31), *Anpep, Epha4*[36],*Maged2, Acsl1,* and *Scn7a* (Supplementary Table 18).

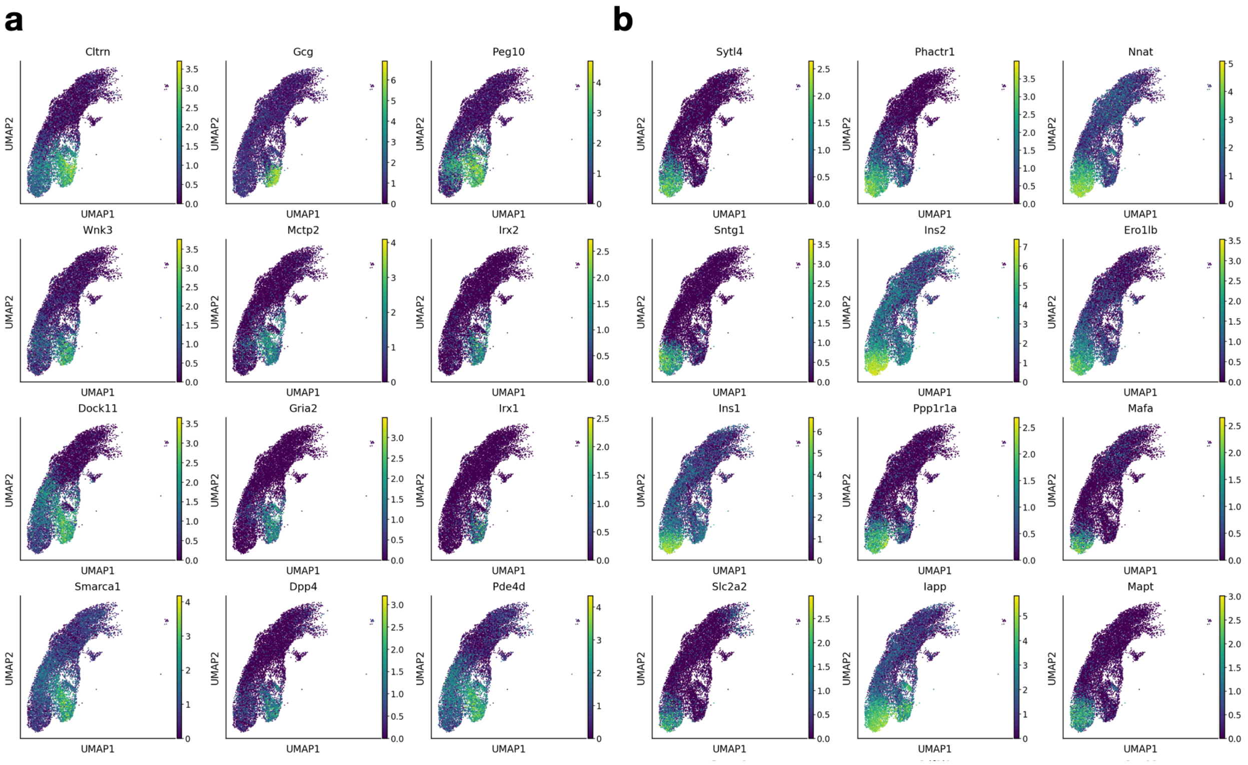

Supplementary Fig. 34 | Driver genes computed by moscot.time for alpha and beta cells

a. Driver genes of alpha cells computed with moscot.time. We recover well-known genes such as *Gcg, Peg10*[37]*, Wnk3*[38]*, Mctp2*[32]*, Irx2*[13], *Irx1*[20], *Smarca1*[38], *Pde4d*[38] but also less reported ones like *Cltrn, Dock11, Gria2,* and *Dpp4* (Supplementary Table 13). **b.** Driver genes of beta cells identified with moscot. We recover known genes such as *Ins1, Ins2, Mafa*[39], *Sytl4*[40], *Nnat*[41], *Slc2a2*[42], *Ppp1r1a*[43], *Ero1lb*[40], and *Iapp*[44]*,* but also less known ones like *Phactr1, Sntg1, Mapt* (Supplementary Table 14).

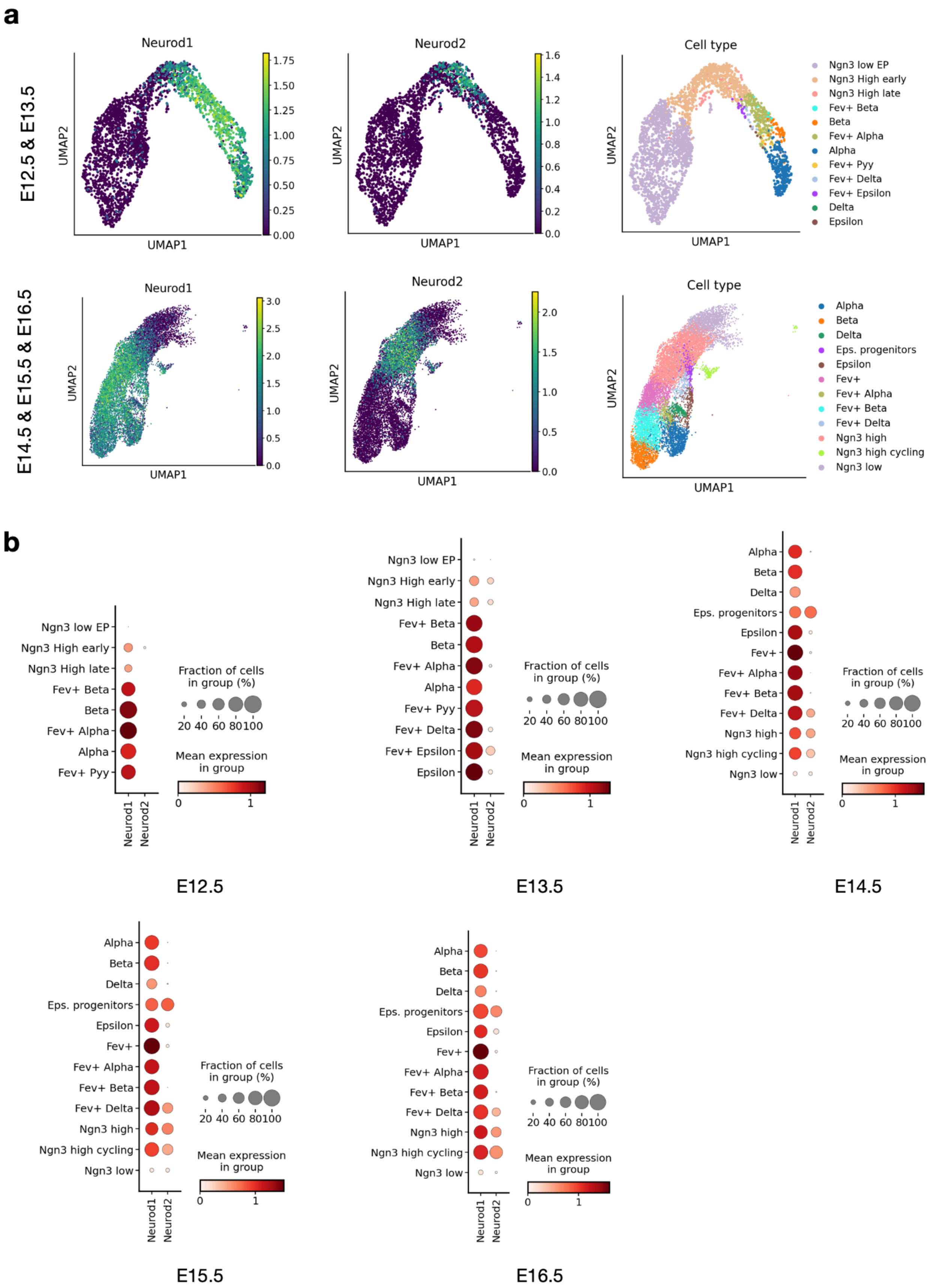
a
E12.5 & E13.5
E14.5 & E15.5 & E16.5
Neurod1
Neurod2
Cell type
UMAP1
UMAP2
Ngn3 low EP
Ngn3 High early
Ngn3 High late
Fev+ Beta
Beta
Fev+ Alpha
Alpha
Fev+ Pyy
Fev+ Delta
Fev+ Epsilon
Delta
Epsilon
Eps. progenitors
Fev+
Ngn3 high
Ngn3 high cycling
Ngn3 low
b
Fraction of cells in group (%)
20 40 60 80100
Mean expression in group
E12.5
E13.5
E14.5
E15.5
E16.5

Supplementary Fig. 35 | *Neurod1* and *Neurod2* expression in the course of pancreatic development

a. Normalized expression of *Neurod1* and *Neurod2* for time point E12.5 and E13.5 in the dataset published by Bastidas-Ponce et al.[17] (top), and normalized expression in our multiome dataset for time points E14.5, E15.5 and E16.5, as well as respective UMAPs colored by cell type. **b.** Mean expression of *Neurod1* and *Neurod2* per cell type across different developmental stages. Only cell types comprising at least 3 cells are kept.

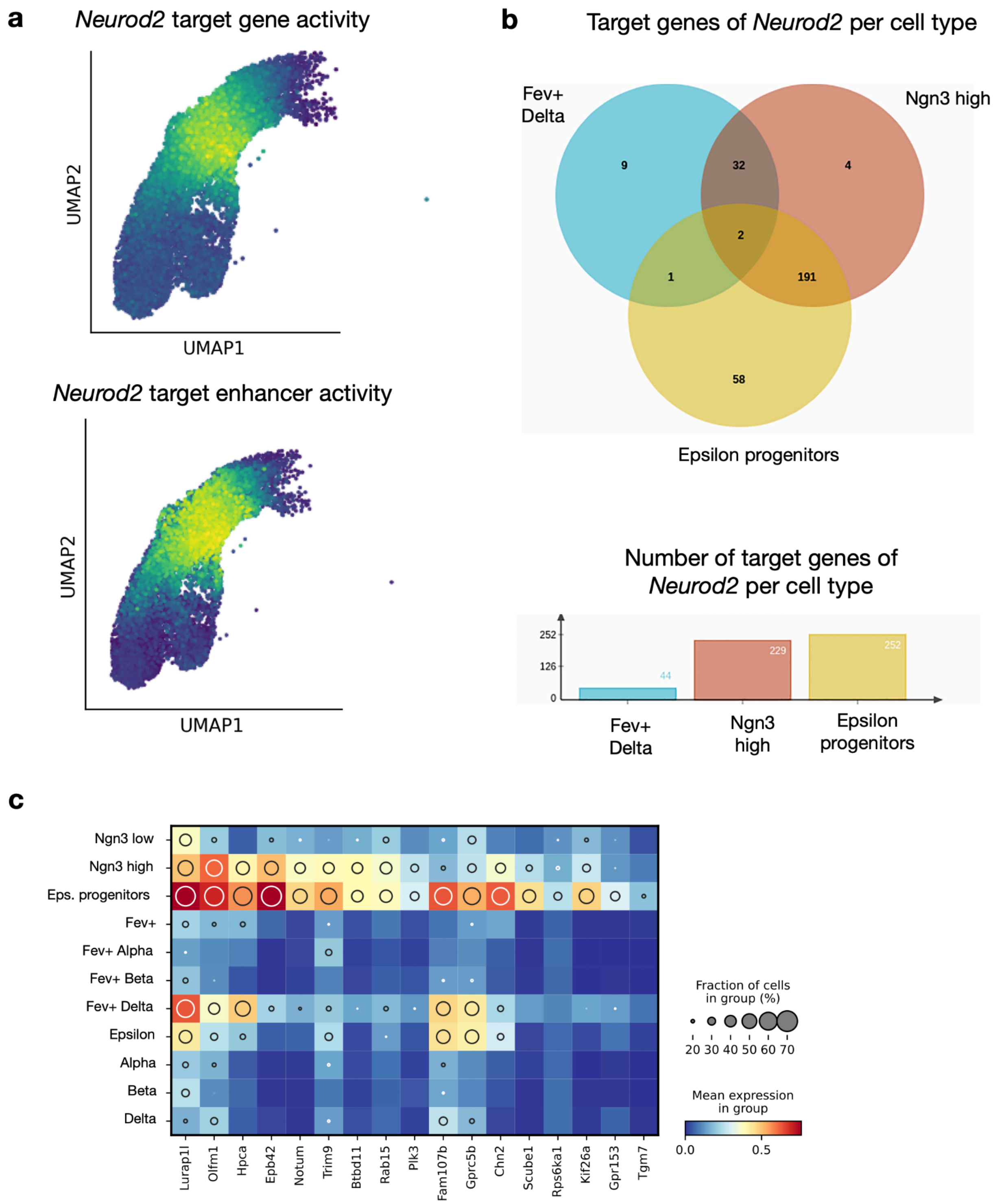

Supplementary Fig. 36 | Regulatory analysis of *Neurod2* identifies target genes

a. Expression of predicted target genes of *Neurod2* and accessibility of predicted enhancers of *Neurod2* based on the eRegulon computed with Scenic+[45]. **b.** Expression of target genes of *Neurod2* in cell types of interest, with thresholds provided by Scenic+ (Supplementary Table 29).

c. Expression levels of a subset of *Neurod2* target genes (predicted by Scenic+) in different endocrine clusters (scaled heatmap, Supplementary Tables 23-28).

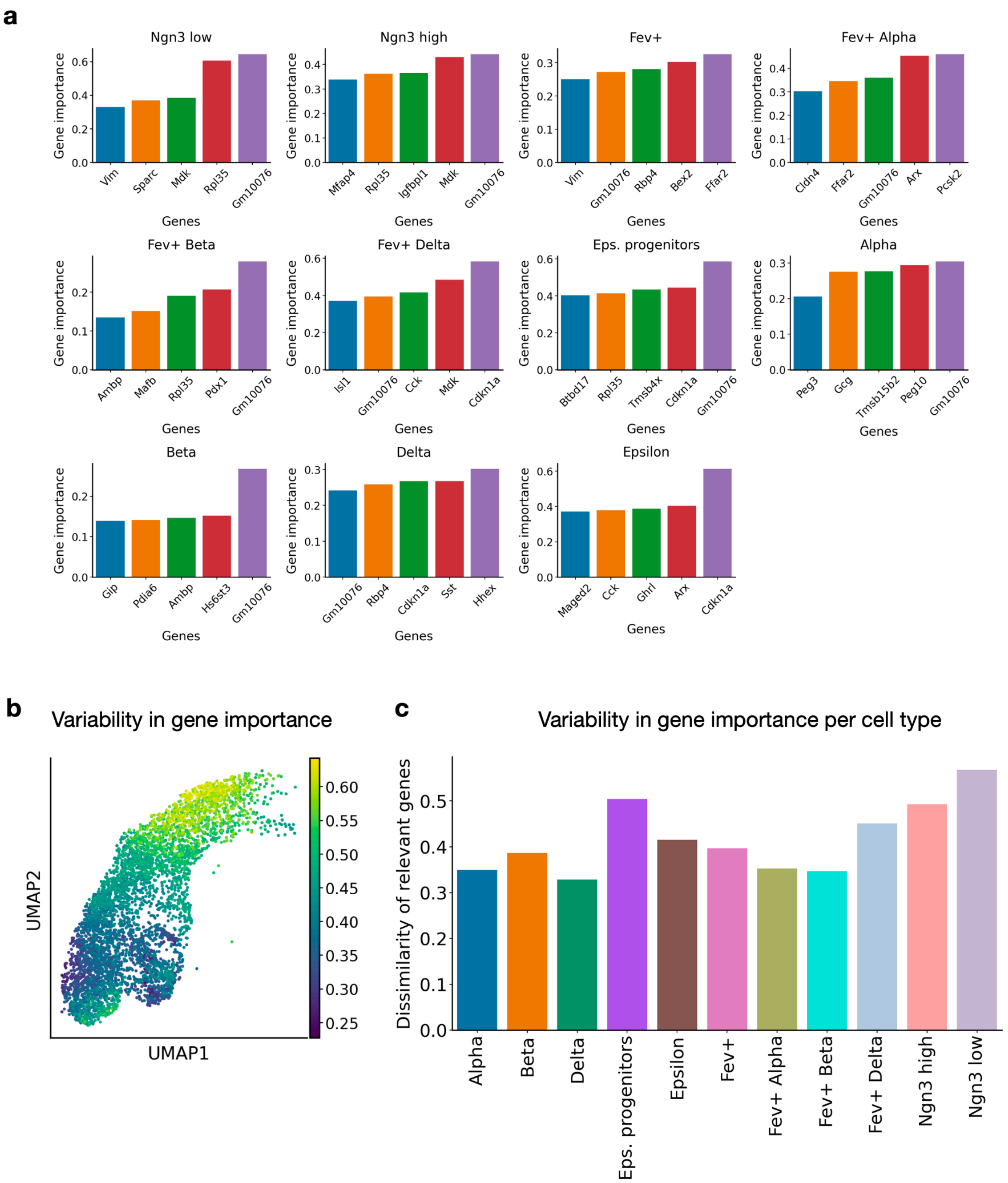

Supplementary Fig. 37 | Feature-level interpretation of optimal transport maps using Sparse Monge

a. Gene importance per cell type in the pancreatic endocrinogenesis dataset using Sparse Monge[18] (Methods). **b.** The variability in gene importance computed based on feature-sparse transport maps recovers lineage branching events. The higher the variability, the higher

plasticity a cell has (Methods). **c.** Aggregation of the variability in gene importance to cell type level.

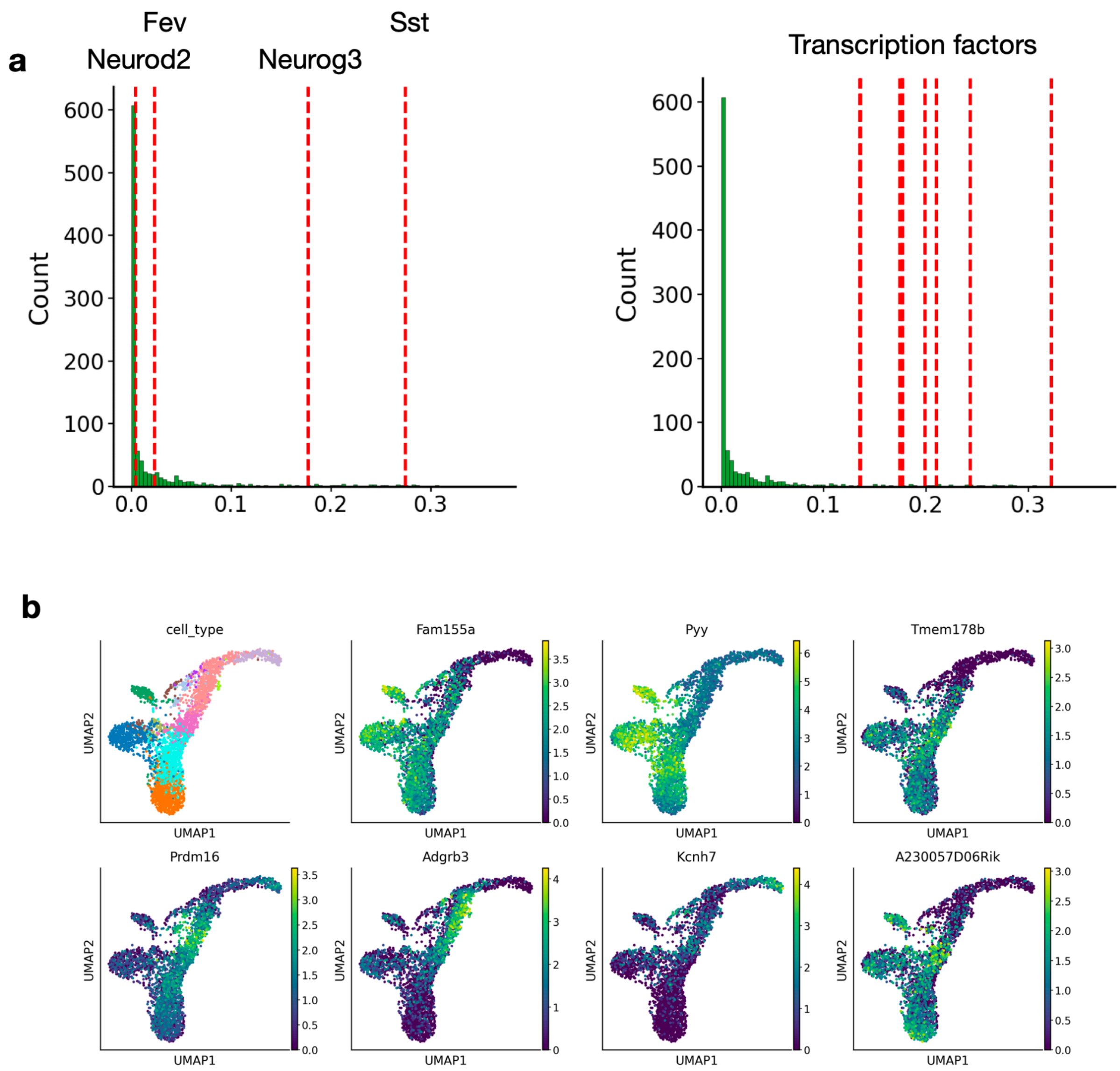

Supplementary Fig. 38 | Feature importance by considering the influence on a transport map

a. Left: Subset of marker genes in pancreatic endocrinogenesis (*Neurod2* as activator of the epsilon lineage, *Fev* as activator of the alpha and beta lineage, *Neurog3* as activator of endocrine formation and *Sst* as hormone emitted by delta cells) and their corresponding significance when leaving them out from the gene set for the computation of the optimal transport matrix. Right: The nine most significant features identified with the same procedure which are also transcription factors (from left to right: *Sox4*[19], *Mctp2, Meis1, Neurog3*[20], *Sim1*[21], *Etv1*[22], *Cbfa2t2, Cers6, Prdm16*[23]). **b.** UMAP embedding of the considered cells, colored by cell type and normalized gene expression corresponding to the 7 most significant genes.

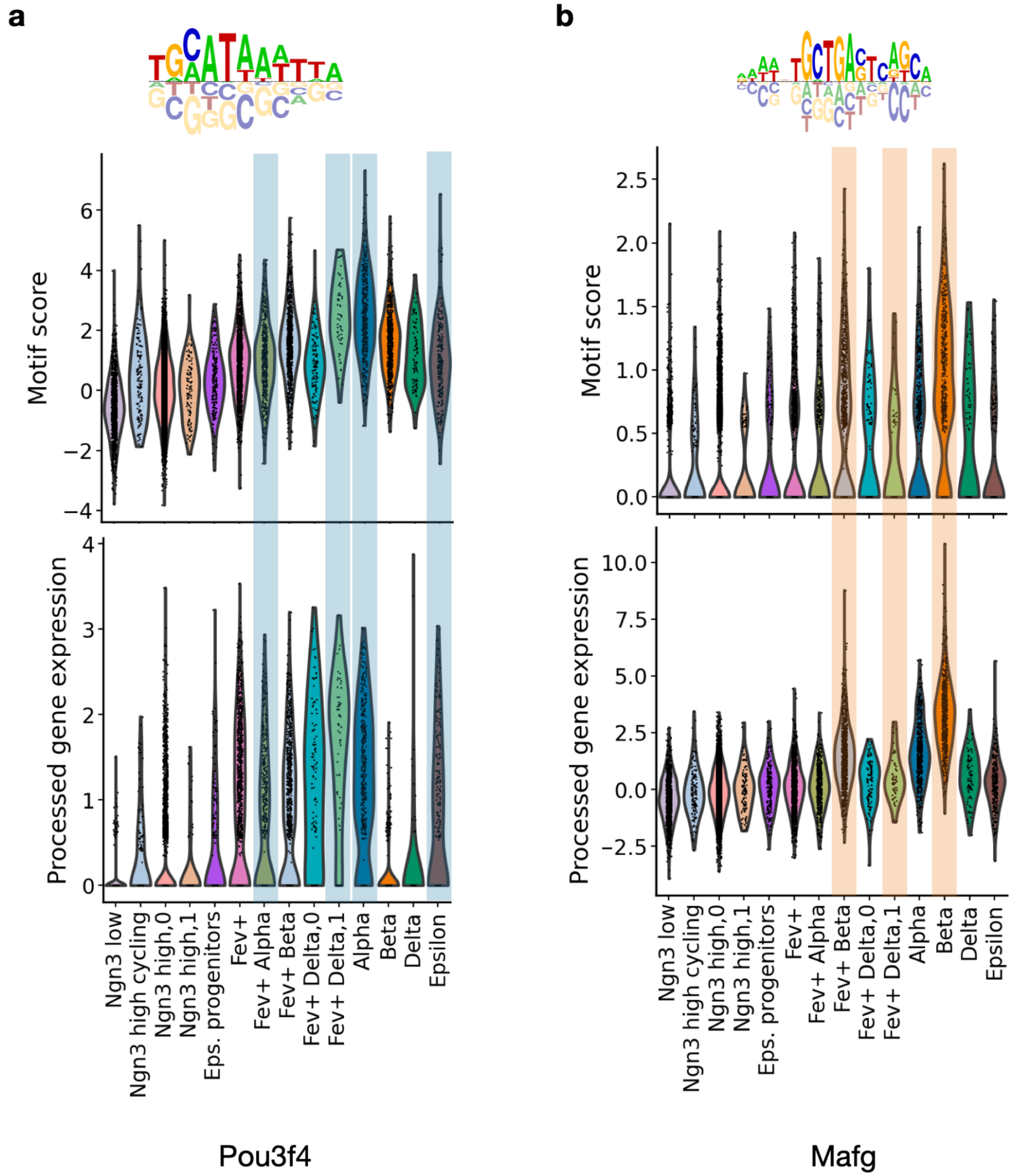

Supplementary Fig. 39 | Alpha and beta motif activity calculated with moscot.time

a. Motif with cisBP identifier M03318_2.00, which we identified as alpha cell marker motif using moscot.time and differential motif activity test. The upper plot shows the motif score computed by ChromVar[46], while the lower one shows the processed (normalized, log1p-transformed) gene expression of the associated gene *Pou3f4* (Methods). Alpha cells and their conjectured

progenitors are underlaid in blue. For a motif to be active both the motif activity score and the gene expression should be high (Supplementary Table 32). **b.** Motif with cisBP identifier M08835_2.00, which we identified as a marker motif for the beta cell population. Again, direct beta cell progenitors are underlaid (Supplementary Table 33).

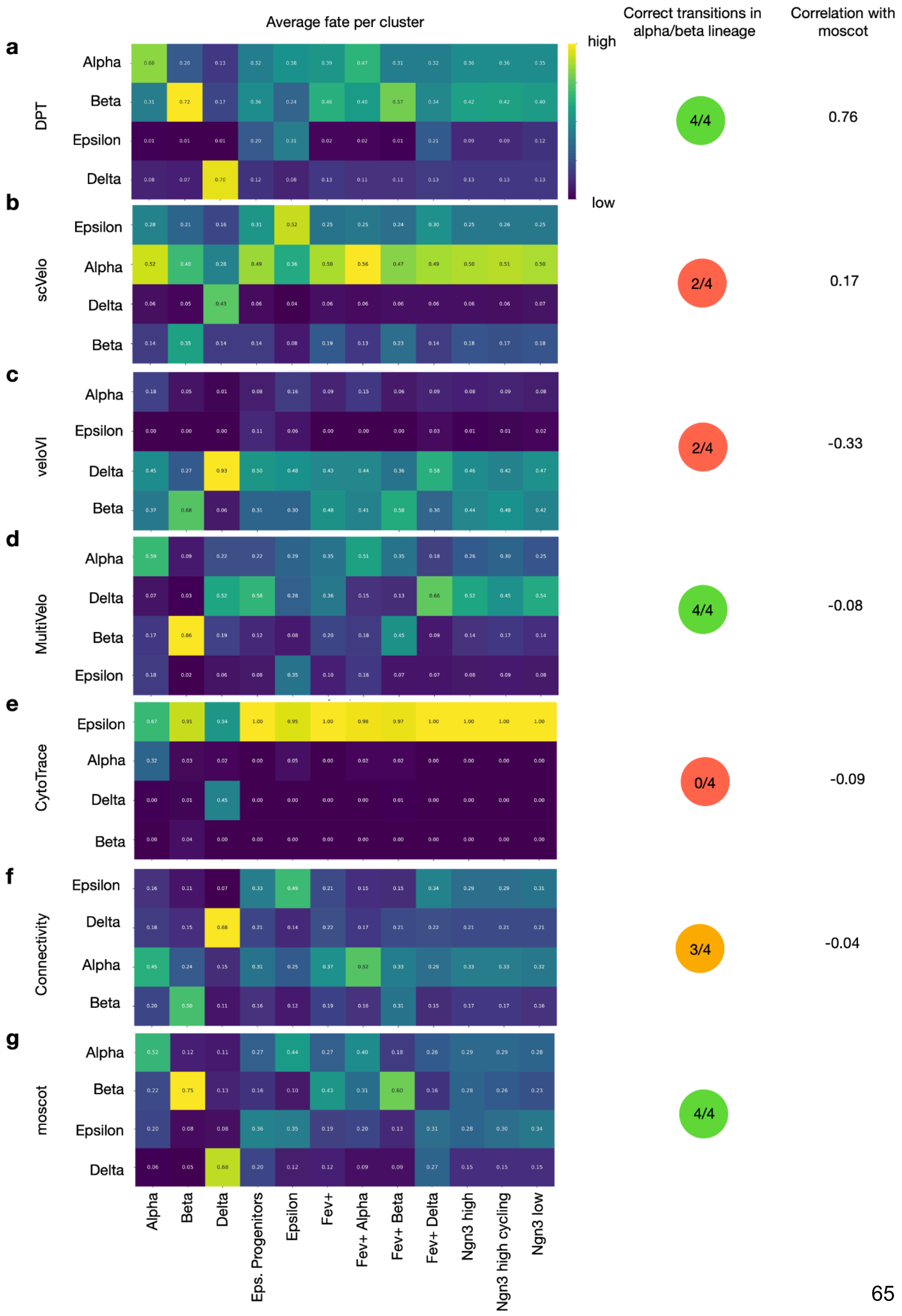
Average fate per cluster
Correct transitions in alpha/beta lineage
Correlation with moscot
high
low
a
DPT
Alpha
Beta
Epsilon
Delta
4/4
0.76
b
scVelo
Epsilon
Alpha
Delta
Beta
2/4
0.17
c
veloVI
Alpha
Epsilon
Delta
Beta
2/4
-0.33
d
MultiVelo
Alpha
Delta
Beta
Epsilon
4/4
-0.08
e
CytoTrace
Epsilon
Alpha
Delta
Beta
0/4
-0.09
f
Connectivity
Epsilon
Delta
Alpha
Beta
3/4
-0.04
g
moscot
Alpha
Beta
Epsilon
Delta
4/4
Alpha
Beta
Delta
Eps. Progenitors
Epsilon
Fev+
Fev+ Alpha
Fev+ Beta
Fev+ Delta
Ngn3 high
Ngn3 high cycling
Ngn3 low

Supplementary Fig. 40 | Analysis of the pancreatic endocrinogenesis dataset with different trajectory inference methods

Trajectory analysis of the pancreatic endocrinogenesis dataset with commonly used TI methods (Methods). We use CellRank[47,48] to compute fate probabilites to the four endocrine cell states alpha, beta, delta and epsilon for each single cell followed by aggregation to cell type level (Methods) based on **a.** diffusion pseudotime[49], **b.** scVelo[50], **c.** VeloVI[51], **d.** MultiVelo[52], **e.** CytroTrace [53], and **f.** connectivity of the graph[47]. We use moscot's RealTimeKernel to predict compute aggregated fate probabilities (**g**), highlighting these are outputs from CellRank and hence are different from cell type transitions computed directly with moscot. The quality of the inferred trajectories are assessed based on the alpha and beta lineage (Methods), while the Pearson correlation quantifies the similarity with moscot's predictions.

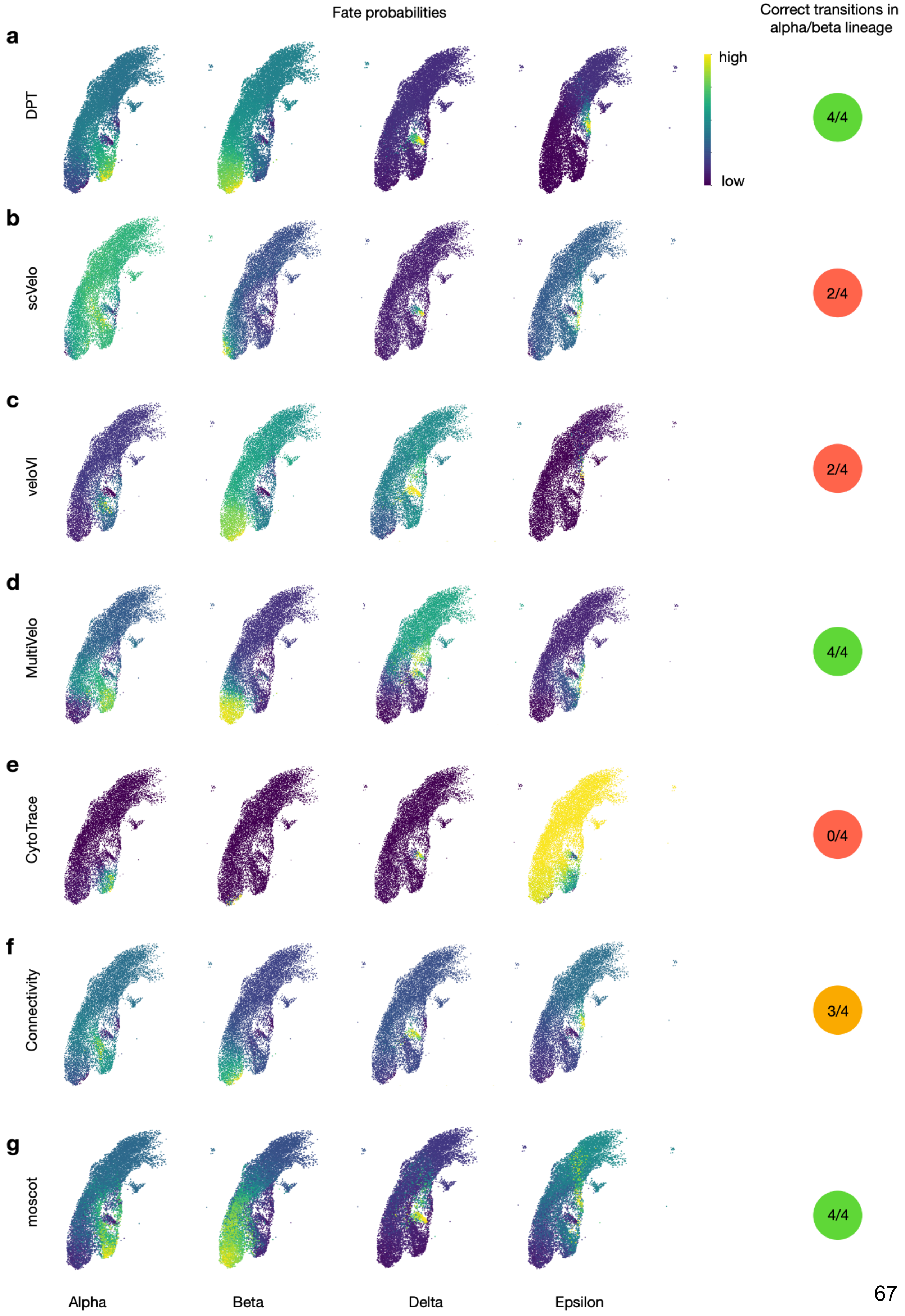

Fate probabilities
Correct transitions in alpha/beta lineage
a
DPT
high
low
4/4
b
scVelo
2/4
c
veloVI
2/4
d
MultiVelo
4/4
e
CytoTrace
0/4
f
Connectivity
3/4
g
moscot
4/4
Alpha
Beta
Delta
Epsilon

Supplementary Fig. 41 | Fate probabilities of the pancreatic endocrinogenesis dataset predicted with different trajectory inference methods

Trajectory analysis of the pancreatic endocrinogenesis dataset with commonly used TI methods (Methods). We use CellRank[47,48] to compute fate probabilities to the four endocrine cell states alpha, beta, delta and epsilon (Methods) based on **a.** diffusion pseudotime[49], **b.** scVelo[50], **c.** VeloVI[51], **d.** MultiVelo[52], **e.** CytroTrace[53], **g.** connectivity of the graph[47], and **f.** moscot. The quality label is inherited from Supplementary Fig. 40 .

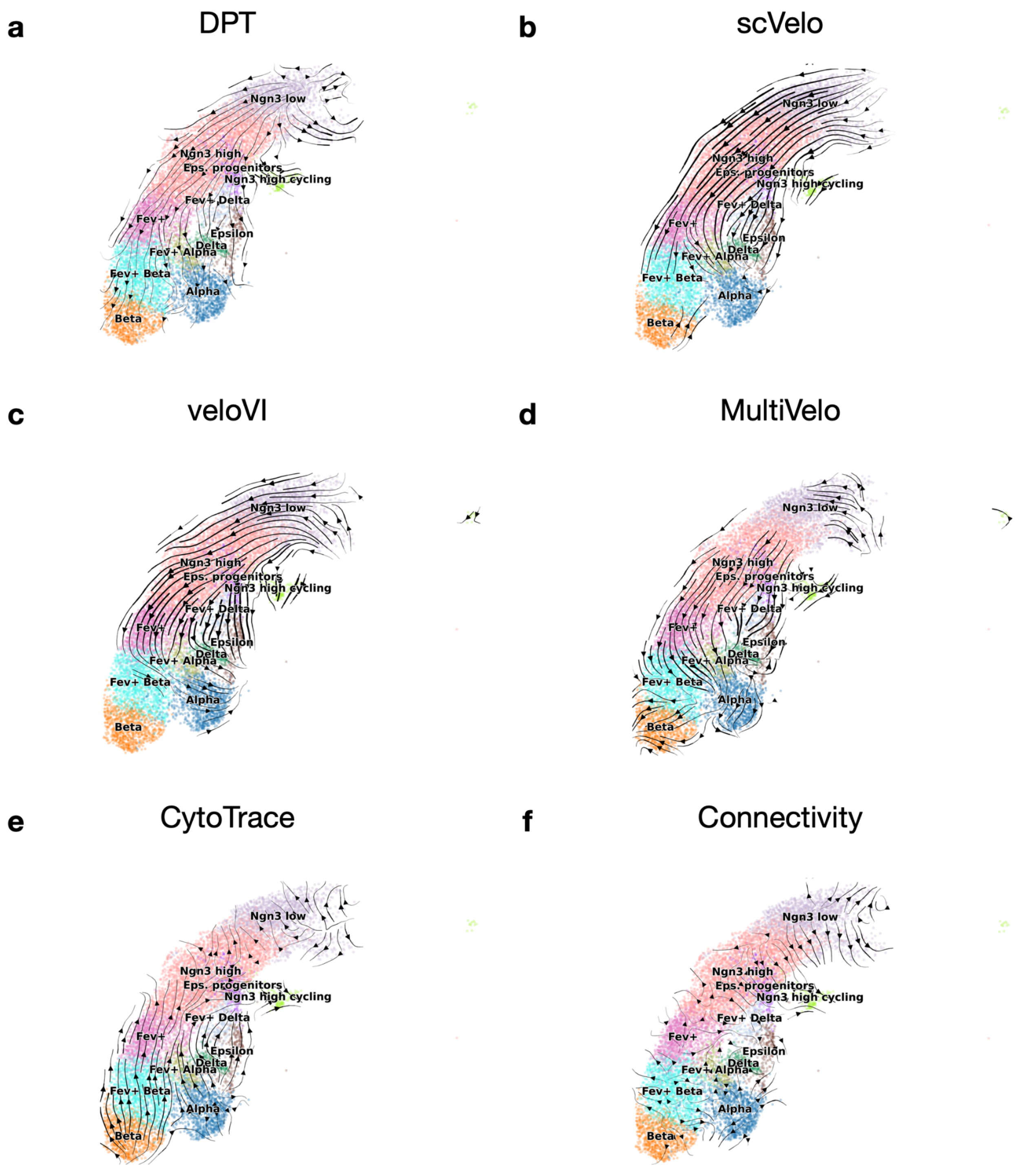

Supplementary Fig. 42 |Stream embedding plots of the dynamics obtained by different trajectory inference methods on the pancreatic endocrinogenesis

Trajectory analysis of the pancreatic endocrinogenesis dataset with commonly used TI methods (Methods). We use CellRank[47,48] to plot the dynamics learnt with **a.** diffusion pseudotime[49], **b.** scVelo[50], **c.** VeloVI [51], **d.** MultiVelo[52], **e.** CytroTrace[53], and **f.** connectivity of the graph[47].

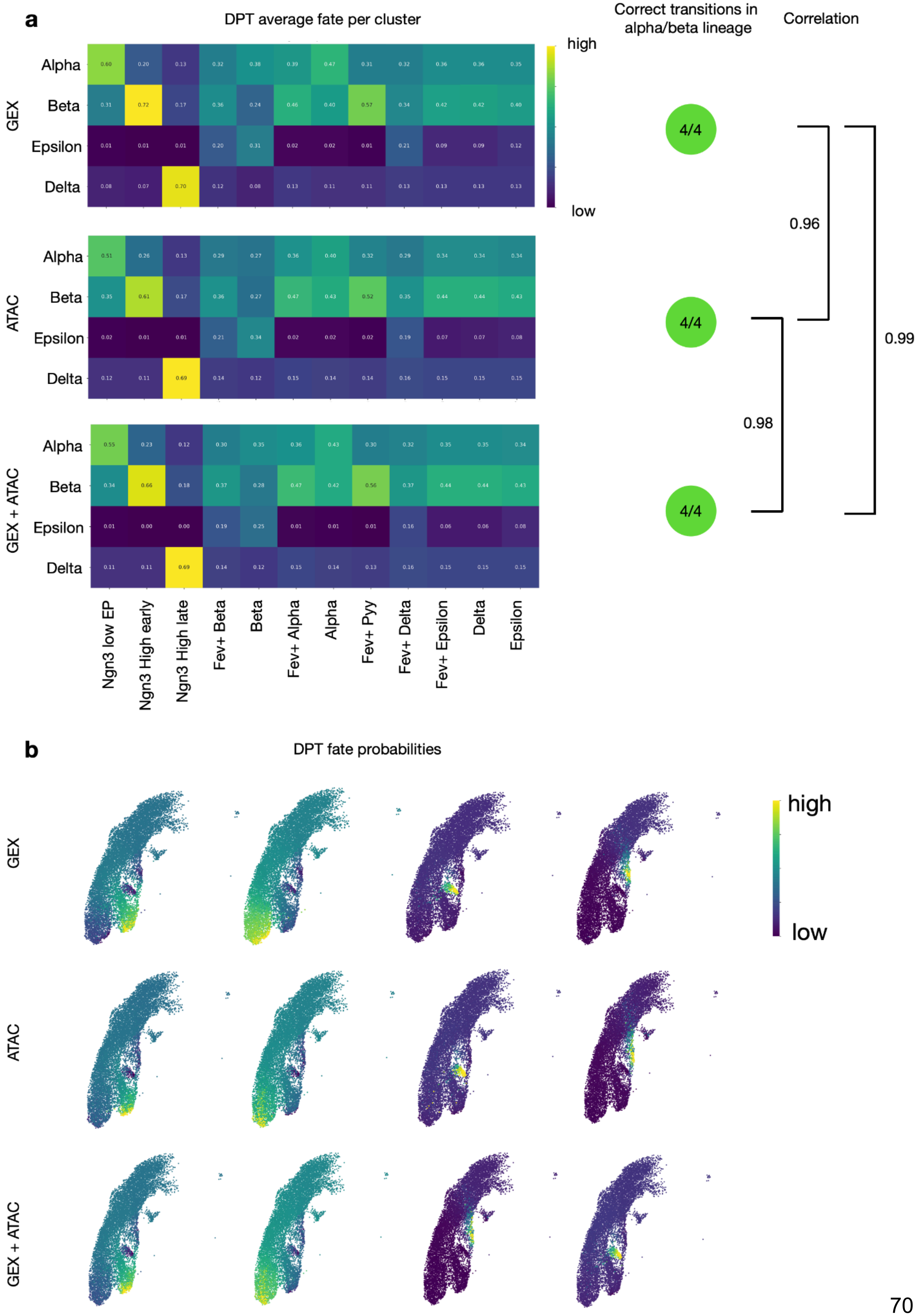
a
DPT average fate per cluster
Correct transitions in alpha/beta lineage
Correlation
GEX
ATAC
GEX + ATAC
Alpha
Beta
Epsilon
Delta
high
low
4/4
4/4
4/4
0.96
0.98
0.99
Ngn3 low EP
Ngn3 High early
Ngn3 High late
Fev+ Beta
Beta
Fev+ Alpha
Alpha
Fev+ Pyy
Fev+ Delta
Fev+ Epsilon
Delta
Epsilon
b
DPT fate probabilities
GEX
ATAC
GEX + ATAC
high
low

Supplementary Fig. 43 | Fate predictions of diffusion pseudotime are stable with respect to underlying modality

a. Aggregated fate probabilities of diffusion pseudotime[49] computed with CellRank. The quality is assessed based on the alpha and beta lineage (Methods), and correlations are computed between aggregated fate probabilities (Methods). **b.** Fate probabilities computed with CellRank based on diffusion pseudotime predictions incorporating different modalities.

References

1. Cuturi, M. *et al.* Optimal Transport Tools (OTT): A JAX Toolbox for all things Wasserstein. *arXiv [cs.LG]* (2022).
2. Demetci, P., Santorella, R., Sandstede, B., Noble, W. S. & Singh, R. SCOT: Single-Cell Multi-Omics Alignment with Optimal Transport. *J. Comput. Biol.* **29**, 3–18 (2022).
3. Qiu, C. *et al.* Systematic reconstruction of cellular trajectories across mouse embryogenesis. *Nat. Genet.* **54**, 328–341 (2022).
4. Schiebinger, G. *et al.* Optimal-Transport Analysis of Single-Cell Gene Expression Identifies Developmental Trajectories in Reprogramming. *Cell* **176**, 928–943.e22 (2019).
5. Wolf, F. A., Angerer, P. & Theis, F. J. SCANPY: large-scale single-cell gene expression data analysis. *Genome Biol.* **19**, 15 (2018).
6. Li, B. *et al.* Benchmarking spatial and single-cell transcriptomics integration methods for transcript distribution prediction and cell type deconvolution. *Nat. Methods* 1–9 (2022).
7. Zeira, R., Land, M., Strzalkowski, A. & Raphael, B. J. Alignment and integration of spatial transcriptomics data. *Nat. Methods* **19**, 567–575 (2022).
8. Jones, A., William Townes, F., Li, D. & Engelhardt, B. E. Alignment of spatial genomics and histology data using deep Gaussian processes. *bioRxiv* 2022.01.10.475692 (2022) doi:10.1101/2022.01.10.475692.
9. Myronenko, A. & Song, X. Point set registration: coherent point drift. *IEEE Trans. Pattern Anal. Mach. Intell.* **32**, 2262–2275 (2010).
10. Liu, X., Zeira, R. & Raphael, B. J. Partial alignment of multislice spatially resolved transcriptomics data. *Genome Res.* **33**, 1124–1132 (2023).
11. Wang, M. *et al.* High-resolution 3D spatiotemporal transcriptomic maps of developing Drosophila embryos and larvae. *Dev. Cell* **57**, 1271–1283.e4 (2022).

12. Schwabe, D., Formichetti, S., Junker, J. P., Falcke, M. & Rajewsky, N. The transcriptome dynamics of single cells during the cell cycle. *Mol. Syst. Biol.* **16**, e9946 (2020).
13. Yu, X.-X. *et al.* Sequential progenitor states mark the generation of pancreatic endocrine lineages in mice and humans. *Cell Res.* **31**, 886–903 (2021).
14. la O Sean, D. *et al.* Single-Cell Multi-Omic Roadmap of Human Fetal Pancreatic Development. *bioRxiv* 2022.02.17.480942 (2022) doi:10.1101/2022.02.17.480942.
15. Szlachcic, W. J., Ziojla, N., Kizewska, D. K., Kempa, M. & Borowiak, M. Endocrine Pancreas Development and Dysfunction Through the Lens of Single-Cell RNA-Sequencing. *Front Cell Dev Biol* **9**, 629212 (2021).
16. van der Meulen, T. *et al.* Virgin Beta Cells Persist throughout Life at a Neogenic Niche within Pancreatic Islets. *Cell Metab.* **25**, 911–926.e6 (2017).
17. Bastidas-Ponce, A. *et al.* Comprehensive single cell mRNA profiling reveals a detailed roadmap for pancreatic endocrinogenesis. *Development* **146**, (2019).
18. Cuturi, M., Klein, M. & Ablin, P. Monge, Bregman and Occam: Interpretable Optimal Transport in High-Dimensions with Feature-Sparse Maps. *arXiv [stat.ML]* (2023).
19. Xu, E. E. *et al.* SOX4 cooperates with neurogenin 3 to regulate endocrine pancreas formation in mouse models. *Diabetologia* **58**, 1013–1023 (2015).
20. Schreiber, V. *et al.* Extensive NEUROG3 occupancy in the human pancreatic endocrine gene regulatory network. *Mol Metab* **53**, 101313 (2021).
21. Petersen, M. B. K. *et al.* Single-Cell Gene Expression Analysis of a Human ESC Model of Pancreatic Endocrine Development Reveals Different Paths to β-Cell Differentiation. *Stem Cell Reports* **9**, 1246–1261 (2017).
22. Heeg, S. *et al.* ETS-Transcription Factor ETV1 Regulates Stromal Expansion and Metastasis in Pancreatic Cancer. *Gastroenterology* **151**, 540–553.e14 (2016).
23. Jiang, N., Yang, M., Han, Y., Zhao, H. & Sun, L. PRDM16 Regulating Adipocyte Transformation and Thermogenesis: A Promising Therapeutic Target for Obesity and

Diabetes. *Front. Pharmacol.* **13**, 870250 (2022).

24. Dichmann, D. S., Yassin, H. & Serup, P. Analysis of pancreatic endocrine development in GDF11-deficient mice. *Dev. Dyn.* **235**, 3016–3025 (2006).
25. Saito, Y. Selenoprotein P as a significant regulator of pancreatic β cell function. *J. Biochem.* **167**, 119–124 (2020).
26. Dominguez Gutierrez, G. *et al.* Gene Signature of Proliferating Human Pancreatic α Cells. *Endocrinology* **159**, 3177–3186 (2018).
27. Atla, G. *et al.* Genetic regulation of RNA splicing in human pancreatic islets. *Genome Biol.* **23**, 196 (2022).
28. Mastrolia, V. *et al.* Loss of α2δ-1 Calcium Channel Subunit Function Increases the Susceptibility for Diabetes. *Diabetes* **66**, 897–907 (2017).
29. Krentz, N. A. J. *et al.* Single-Cell Transcriptome Profiling of Mouse and hESC-Derived Pancreatic Progenitors. *Stem Cell Reports* **11**, 1551–1564 (2018).
30. Taneera, J. *et al.* Identification of novel genes for glucose metabolism based upon expression pattern in human islets and effect on insulin secretion and glycemia. *Hum. Mol. Genet.* **24**, 1945–1955 (2015).
31. Ning, S.-L. *et al.* Different downstream signalling of CCK1 receptors regulates distinct functions of CCK in pancreatic beta cells. *Br. J. Pharmacol.* **172**, 5050–5067 (2015).
32. Duvall, E. *et al.* Single-cell transcriptome and accessible chromatin dynamics during endocrine pancreas development. *Proc. Natl. Acad. Sci. U. S. A.* **119**, e2201267119 (2022).
33. Keller, M. P. *et al.* Genetic Drivers of Pancreatic Islet Function. *Genetics* **209**, 335–356 (2018).
34. Hrovatin, K. *et al.* Delineating mouse β-cell identity during lifetime and in diabetes with a single cell atlas. *bioRxiv* 2022.12.22.521557 (2022) doi:10.1101/2022.12.22.521557.
35. Arnes, L., Hill, J. T., Gross, S., Magnuson, M. A. & Sussel, L. Ghrelin expression in the mouse pancreas defines a unique multipotent progenitor population. *PLoS One* **7**, e52026

(2012).

36. Hutchens, T. & Piston, D. W. EphA4 Receptor Forward Signaling Inhibits Glucagon Secretion From α-Cells. *Diabetes* **64**, 3839–3851 (2015).

37. Isaacson, A. & Spagnoli, F. M. Pancreatic cell fate specification: insights into developmental mechanisms and their application for lineage reprogramming. *Curr. Opin. Genet. Dev.* **70**, 32–39 (2021).

38. Byrnes, L. E. *et al.* Lineage dynamics of murine pancreatic development at single-cell resolution. *Nat. Commun.* **9**, 3922 (2018).

39. Xiafukaiti, G. *et al.* MafB Is Important for Pancreatic β-Cell Maintenance under a MafA-Deficient Condition. *Mol. Cell. Biol.* **39**, (2019).

40. Salinno, C. *et al.* CD81 marks immature and dedifferentiated pancreatic β-cells. *Mol Metab* **49**, 101188 (2021).

41. Wang, X. *et al.* Point mutations in the PDX1 transactivation domain impair human β-cell development and function. *Mol Metab* **24**, 80–97 (2019).

42. Michau, A. *et al.* Mutations in SLC2A2 gene reveal hGLUT2 function in pancreatic β cell development. *J. Biol. Chem.* **288**, 31080–31092 (2013).

43. Salinno, C. *et al.* β-Cell Maturation and Identity in Health and Disease. *Int. J. Mol. Sci.* **20**, (2019).

44. Nishi, M., Sanke, T., Nagamatsu, S., Bell, G. I. & Steiner, D. F. Islet amyloid polypeptide. A new beta cell secretory product related to islet amyloid deposits. *J. Biol. Chem.* **265**, 4173–4176 (1990).

45. Bravo González-Blas, C. *et al.* SCENIC+: single-cell multiomic inference of enhancers and gene regulatory networks. *Nat. Methods* **20**, 1355–1367 (2023).

46. Schep, A. N., Wu, B., Buenrostro, J. D. & Greenleaf, W. J. chromVAR: inferring transcription-factor-associated accessibility from single-cell epigenomic data. *Nature Methods* vol. 14 975–978 Preprint at https://doi.org/10.1038/nmeth.4401 (2017).

47. Lange, M. *et al.* CellRank for directed single-cell fate mapping. *Nat. Methods* **19**, 159–170 (2022).

48. Weiler, P., Lange, M., Klein, M., Pe'er, D. & Theis, F. J. Unified fate mapping in multiview single-cell data. *bioRxiv* 2023.07.19.549685 (2023) doi:10.1101/2023.07.19.549685.

49. Haghverdi, L., Büttner, M., Wolf, F. A., Buettner, F. & Theis, F. J. Diffusion pseudotime robustly reconstructs lineage branching. *Nat. Methods* **13**, 845–848 (2016).

50. Bergen, V., Lange, M., Peidli, S., Wolf, F. A. & Theis, F. J. Generalizing RNA velocity to transient cell states through dynamical modeling. *Nat. Biotechnol.* **38**, 1408–1414 (2020).

51. Gayoso, A. *et al.* Deep generative modeling of transcriptional dynamics for RNA velocity analysis in single cells. *Nat. Methods* **21**, 50–59 (2024).

52. Li, C., Virgilio, M. C., Collins, K. L. & Welch, J. D. Multi-omic single-cell velocity models epigenome–transcriptome interactions and improves cell fate prediction. *Nat. Biotechnol.* **41**, 387–398 (2022).

53. Gulati, G. S. *et al.* Single-cell transcriptional diversity is a hallmark of developmental potential. *Science* **367**, 405–411 (2020).

Supplementary Notes

Contents

1 Fused Gromov-Wasserstein optimization 78

2 Fused Gromov-Wasserstein scalability 78

3 Low-rank Sinkhorn factorization yields linear time and memory complexity 79

4 Background information on pancreatic endocrinogenesis 81

5 Geodesic cost functions 81

6 On the limitations of optimal transport for recovering cell cycles 82

7 Differential accessibility analysis 82

8 Motif activity analysis in pancreatic endocrinogenesis 82

8.1 Motif activity in delta cells . . . 82

8.2 Motif activity in epsilon cells . . . 83

8.3 Motif activity in alpha cells . . . 83

8.4 Motif activity in beta cells . . . 83

1 Fused Gromov-Wasserstein optimization

Consider a generic FWG-type problem,

$$P^* := \underset{P \in U(\boldsymbol{a},\boldsymbol{b})}{\operatorname{argmin}} \; \alpha \sum_{ijkl} L\left(C^X_{ij}, C^Y_{kl}\right) P_{ik} P_{jl} + (1-\alpha) \sum_{ik} C_{ik} P_{ik} - \epsilon H(P)\,, \tag{1}$$

for within-space cost matrices $C^X \in \mathbb{R}^{N\times N}$ and $C^Y \in \mathbb{R}^{M\times M}$, across-space cost matrix $C \in \mathbb{R}^{N\times M}$, distance metric L, weight parameter $\alpha \in [0,1]$ and entropic regularization $H(P)$ at strength ϵ. To write this optimization problem in shorter form, we define the 4-tensor[1]

$$\mathcal{T}(C^X, C^Y)_{ijkl} := L\left(C^X_{ik}, C^Y_{jl}\right)\,, \tag{2}$$

which allows us to rewrite Equation (1) as,

$$P^* = \underset{P \in U(\boldsymbol{a},\boldsymbol{b})}{\operatorname{argmin}} \; \alpha \left\langle \mathcal{T}(C^X, C^Y) \otimes P, P \right\rangle + (1-\alpha)\left\langle C, P\right\rangle - \epsilon H(P)\,, \tag{3}$$

where tensor multiplication is defined as

$$(\mathcal{T} \otimes P)_{ij} := \sum_{kl} \mathcal{T}_{ijkl} P_{kl}\,. \tag{4}$$

Reduce to Sinkhorn iterations. We next derive an algorithm in terms of Sinkhorn iterations; the basic idea is to use projected gradient descent with update rule[1]

$$P^{(l+1)} = \operatorname{Proj}^{\mathrm{KL}}_{U(\boldsymbol{a},\boldsymbol{b})}\left(P^{(l)} \odot e^{-\tau \nabla J(P)|_{P^{(l)}}}\right), \tag{5}$$

where $\operatorname{Proj}^{\mathrm{KL}}_{U(\boldsymbol{a},\boldsymbol{b})}(\tilde{P}) = \operatorname{argmin}_{P\in U(\boldsymbol{a},\boldsymbol{b})} \sum_{ij} P_{ij} \log\left(P_{ij}/\tilde{P}_{ij}\right)$ is a KL projection operator, τ is a step size, J is the FGW objective function defined in Equation (3) and $\odot$ denotes element-wise multiplication. We may rewrite the objective function gradient as

$$\nabla J = (1-\alpha) C + \alpha \mathcal{T}\left(C^X, C^Y\right) \otimes P\,. \tag{6}$$

Further, the KL projection can be solved via an OT problem[2],

$$\operatorname{Proj}^{\mathrm{KL}}_{U(\boldsymbol{a},\boldsymbol{b})}(\tilde{P}) = \underset{P \in U(\boldsymbol{a},\boldsymbol{b})}{\operatorname{argmin}} \left\langle -\epsilon \log \tilde{P}, P\right\rangle - \epsilon H(P)\,. \tag{7}$$

Using Equation (6), Equation (7) and setting $\tau = 1/\epsilon$, we can re-write the update rule of Equation (5) as

$$P^{(l+1)} = \underset{P \in U(\boldsymbol{a},\boldsymbol{b})}{\operatorname{argmin}} \left\langle (1-\alpha) C + \alpha \mathcal{T}\left(C^X, C^Y\right) \otimes P^{(l)}, P\right\rangle - \epsilon H(P)\,, \tag{8}$$

which is the entropically regularized W-type OT problem (Methods). We update the cost matrix at each outer iteration and solve the resulting W-type OT problem using the Sinkhorn algorithm[1,3,4].

2 Fused Gromov-Wasserstein scalability

Throughout this section, we assume equal cell/sample numbers across both datasets for simplicity, i.e., $N = M$. Further, we suppose entropic regularization is applied to the FGW-type OT problem[1,5] and the mirror descent algorithm (Section 1) is used for optimization.

Cubic time complexity for separable loss function L. The major computational bottleneck in FGW[1,5] optimization is the update of the tensor product of Equation (4) required at each update of Equation (8), which is quartic in cell number for general loss function L (ref.[1] and Section 1). However, this can be improved if L is given by a separable loss function. For simplicity, suppose L is given by a squared l_2 loss function, this leads to

$$\mathcal{T}(C^X, C^Y) \otimes P^{(l)} = \underbrace{(C^X)^2 \boldsymbol{a}\, \mathbf{1}_M^\top + \mathbf{1}_N\, \boldsymbol{b}^\top (C^Y)^2}_{\text{constant}} - 2C^X P^{(l)} C^{Y^\top}, \tag{9}$$

which may be evaluated in time $\mathcal{O}(N^3)$ and memory $\mathcal{O}(N^2)$. This result holds beyond the squared l_2 norm for a class of separable loss functions, including the KL divergence[1].

Quadratic time complexity for low-rank C^X and C^Y. For the second, non-constant term, further suppose both C^X and C^Y result from the application of a squared l_2 distance metric such that they admit low-rank factorizations,

$$C^X = A^X B^{X^\top} \text{ for } A^X, B^X \in \mathbb{R}^{N \times (D_x+2)}, \tag{10}$$

$$C^Y = A^Y B^{Y^\top} \text{ for } A^Y, B^Y \in \mathbb{R}^{M \times (D_y+2)}, \tag{11}$$

for within-space dimensions D_x, D_y (ref.[6]). Such factorizations are obtained by specifying $A^X := [\boldsymbol{p}, \mathbf{1}_N, -2X]$ and $B^X := [\mathbf{1}_N, \boldsymbol{p}, X]$ for $\boldsymbol{p} := [||\boldsymbol{x}_1||_2^2, ..., ||\boldsymbol{x}_N||_2^2]^\top$, and analogously for A^Y and B^Y (Section 3 and ref.[6]). Thus, the non-constant part of Equation (9) may be written as

$$C^X P^{(l)} C^{Y^\top} = A^X B^{X^\top} P^{(l)} B^Y A^{Y^\top}. \tag{12}$$

This can be computed in time $\mathcal{O}(N^2(D_x + D_y))$, i.e., quadratic rather than cubic in the input size[7]. Note that all current `moscot` models use squared euclidean loss functions for all of L, C^X, and C^Y and thus enjoy quadratic time complexity without any approximations. This represents a remarkable speedup compared to previous FGW-based[1,5] models in single-cell genomics[4,8] without any accuracy sacrifice.

For future `moscot` models that might require non-euclidean distance metrics, approximate algorithms exist to compute low-rank factorizations of C^X and C^Y, which scale linearly in sample number[6,9,10]. Thus the overall algorithm time complexity remains quadratic.

Linear time complexity for low-rank P. The quadratic time and memory complexities become prohibitively expensive for atlas-scale datasets with hundreds of thousands of samples per dataset. Thus, we additionally assume low-rank structure in the coupling matrix P. Scetbon et al.[7] recently extended their low-rank Sinkhorn factorization (Section 3) to the FGW-setting[1,5], unlocking linear time and memory-complexity. Low-rank FGW solvers are implemented in OTT[11] and available to all FGW-based `moscot` models, including `moscot.space.mapping`, `moscot.space.alignment` and `moscot.spatiotemporal`.

3 Low-rank Sinkhorn factorization yields linear time and memory complexity

While the engineering improvements introduced in the Methods section allow the application to large datasets through GPU acceleration with linear or quadratic memory complexity for W-or GW-type problems, respectively, they still suffer from quadratic time complexity. Various authors have suggested approximations to the Sinkhorn iterations that yield linear time complexity to overcome this limitation. Altschuler et al.[12] suggest computing a low-rank approximation to the kernel matrix

K using the Nystrom method[13]; their approach remains limited to squared euclidean cost functions c, is non-differentiable and only works for large regularization strength ϵ where inner iterations remain positive.

Forrow et al.[14] suggest a different route that imposes low-rank constraints on the feasible set of couplings $U(\boldsymbol{a}, \boldsymbol{b})$ rather than on the kernel matrix K. Their approach leads to an elegant solution via a barycenter problem; however, it remains limited to squared euclidean cost functions c. Scetbon et al.[6] generalize this approach to arbitrary cost functions c; their proposed solution is differentiable and applicable for a wide range of ϵ values, including no entropic regularization ($\epsilon = 0$). This approach is implemented in OTT and available through `moscot`; we refer to it as *low-rank Sinkhorn*. It has meanwhile been extended from W-type to GW-type problems[7] which is also implemented in OTT[11] and available to FGW-based[1,5] `moscot` models including `moscot.space.mapping`, `moscot.space.alignment`or `moscot.spatiotemporal` (Section 2).

For the low-rank Sinkhorn approach, following Scetbon et al.[6], define the nonnegative rank of a coupling matrix $P \in \mathbb{R}_+^{N \times M}$ to be

$$\mathrm{rk}_+(P) := \min \left\{ q \,\middle|\, P = \sum_{i=1}^{q} R_i \,, \mathrm{rk}(R_i) = 1,\, R_i \in \mathbb{R}_+^{N \times M} \right\} , \tag{13}$$

for rank rk. For $r \geq 1$, we make use of this to define the set of rank-r couplings via

$$U(\boldsymbol{a}, \boldsymbol{b}, r) := \{P \in U(\boldsymbol{a}, \boldsymbol{b}) \,|\, \mathrm{rk}_+(P) \leq r\} \;, \tag{14}$$

where $U(\boldsymbol{a}, \boldsymbol{b})$ is the set of feasible couplings. The rank-constrained feasible set $U(\boldsymbol{a}, \boldsymbol{b}, r)$ allows us to formulate the low-rank OT problem via

$$P^* := \underset{P \in U(\boldsymbol{a}, \boldsymbol{b}, r)}{\mathrm{argmin}} \; \langle P, C \rangle - \epsilon H(P) \,. \tag{15}$$

An explicit characterisation of couplings P in $U(\boldsymbol{a}, \boldsymbol{b}, r)$ is given by

$$P = Q \,\mathrm{diag}\,(1/\boldsymbol{g})\, R^\top \text{ for } \boldsymbol{g} \in \Delta_r^*,\; Q \in U(\boldsymbol{a}, \boldsymbol{g}),\; R \in U(\boldsymbol{b}, \boldsymbol{g}) \,, \tag{16}$$

where Δ_r^* denotes the r-simplex with strictly positive elements. Using this factorization, Scetbon et al.[7] derive a mirror descent optimization scheme for the low-rank OT problem of Equation (15); the time- and memory bottleneck in this algorithm is given by matrix-matrix multiplications of the form CR and $C^\top Q$ for $Q \in \mathbb{R}^{N \times r}$ and $R \in \mathbb{R}^{M \times r}$. Thus, without any assumptions on the cost matrix C, the low-rank approach remains at memory complexity $\mathcal{O}(MN)$ and time complexity $\mathcal{O}(NMr)$.

To improve upon this complexity, assume that C itself admits a low-rank factorization (Section 2), given by

$$C = AB^\top \text{ for } A \in \mathbb{R}^{N \times D},\; B \in \mathbb{R}^{M \times D} \,, \tag{17}$$

such that matrix-matrix multiplications $CR = A(B^\top R)$ and $C^\top Q = B(A^\top Q)$ can be evaluated in memory $\mathcal{O}\left((D + r)(M + N) + Dr\right)$ and time $\mathcal{O}\left(rD(N + M)\right)$, i.e. both linear in the total cell number $N + M$. In particular, such a factorization can be obtained if the cost results from applying a squared euclidean cost function, i.e. $C = c(X, Y) = ||X - Y||_2^2$. In such a case, C may be written as

$$C = \boldsymbol{p}\boldsymbol{1}_M^\top + \boldsymbol{1}_N \boldsymbol{q}^\top - 2XY^\top \,, \tag{18}$$

for $\boldsymbol{p} := [||\boldsymbol{x}_1||_2^2, ..., ||\boldsymbol{x}_N||_2^2]^\top$ and $\boldsymbol{q} := [||\boldsymbol{y}_1||_2^2, ..., ||\boldsymbol{y}_M||_2^2]^\top$. The desired factorization is obtained by defining $A := [\boldsymbol{p}, \boldsymbol{1}_N, -2X] \in \mathbb{R}^{N \times (N_l + 2)}$, $B := [\boldsymbol{1}_M, \boldsymbol{q}, Y] \in \mathbb{R}^{M \times (N_l + 2)}$ for cells $\boldsymbol{x}_i$ and $\boldsymbol{y}_j$ embedded in some latent space of dimension N_l. In general, low-rank factorizations of cost matrices C can be computed in linear time using randomized algorithms as long as the cost function c is given by a proper distance metric[6,9,10].

4 Background information on pancreatic endocrinogenesis

The process of murine pancreatic endocrinogenesis can be divided into two stages. The primary stage is dominated by the creation of multipotent progenitor cells (MPCs), and mainly takes place between embryonic day (E) 9.5 and E12.5[15–17]. During the secondary stage the cells develop into three major lineages, namely Acinar cells, Ductal cells and the endocrine cells. During pancreatic lineage formation, first MPCs give rise to tip and trunk domains. The tip domain is differentiated into acinar cells and the trunk domain contains bipotent progenitors that give rise to ductal cells or endocrine progenitors. The later one is marked by the transient expression of the transcription factor Ngn3 and produce different hormone-producing cell including alpha cells (expressing glucagon), beta cells (insulin), epsilon cells (ghrelin), and delta cells (somatostatin) in a process so called endocrinogenesis.

5 Geodesic cost functions

The quality of a mapping obtained from an OT problem depends on the chosen cost function (Supplementary Figure 22). For example, for time-series scRNA-seq data, we need to quantify the cost associated with transporting cells from t_1 to t_2. This amounts to defining a biologically meaningful distance metric among cells in the underlying space (e.g. gene expression space). Most OT applications to single-cell genomics use euclidean distances in latent spaces, such as PCA or scVI[18] latent space. However, euclidean distances might fail to capture the subtleties of cellular state changes during complex biological processes such as development, regeneration, or cancer. In related fields studying cellular manifolds and trajectories, researchers have successfully employed graph-based distance metrics to avoid the pitfalls of euclidean distances to capture phenotypic manifolds. For example, UMAP[19], t-SNE[20] and diffusion maps[21] use graph-based distance metrics to derive low-dimensional cellular representations, and DPT[22], Wanderlust[23] and Palantir[24] use graph-based distance metrics to estimate cellular trajectories[25].

A common way to define distances on arbitrary domains is considering diffusion processes, and in particular, heat diffusions[26]. Following the discussion about the use of graphs in single-cell genomics, we focus on heat diffusions *on graphs*. For the general case, or the Euclidean case, we refer the reader to text books. The heat kernel on a graph is defined as

$$\mathbf{H}_t = \exp\left(-t\mathbf{L}\right) \tag{19}$$

where $\mathbf{L} = \mathbf{D} - \mathbf{A}$ is the graph Laplacian, with $\mathbf{D}$ denoting the degree matrix and $\mathbf{A}$ denoting the adjacency matrix. In effect, the heat kernel solves the heat equation

$$\frac{\partial}{\partial t}\boldsymbol{f}(t) + \boldsymbol{L}\boldsymbol{f}(t) = \mathbf{0}, \text{ s.t.} \quad \boldsymbol{f}(0) = \boldsymbol{f}_0 \quad t \in \mathbb{R}^+ \tag{20}$$

where $\mathbf{f}_0$ is the signal on the graph at time 0. A challenge in real-world applications is the choice of $\bar{t}$ as well as the choice of (the scale of) the distances between nodes, which affects the adjacency matrix $\mathbf{A}$ and hence the Laplacian matrix $\mathbf{L}$.

In the context of single-cell genomics, we consider N cells at t_1 and M cells at t_2, and then compute a joint single-cell k-nearest neighbor (kNN) graph $\mathcal{G}$. In particular, we use the connectivities computed from `scanpy.pp.neighbors` as adjacency matrix. This allows to compute a graph laplacian matrix $L \in \mathbb{R}^{(M+N)\times(M+N)}$.

The main challenge of solving the heat equation is the computation of the matrix exponential. Hence, Huguet et al.[27], Solomon et al.[28] suggest to approximate the matrix exponential using an implicit Euler scheme [28] or Chebyshev polynomials [28]. In particular, they choose $t = \frac{\epsilon}{4}$ with ϵ denoting the entropy regularisation parameter, which allows to use the heat kernel $\mathcal{H}_t$ as drop in replacement for

the usual Gibbs kernel (Methods), i.e.

$$K(x, y) = \mathcal{H}_t(x, y)\,, \tag{21}$$

for cells x and y, and heat diffusion parameter t. Taking advantage of the sparsity of kNN graphs, such an approach reduces the time complexity of Sinkhorn iterations to be linear in cell number $(N + M)$.

Another motivation to use heat diffusion is Varadhan's formula [29]. It connects the heat kernel with the geodesic distance. In effect

$$\lim_{t\to 0} -4t \log \mathcal{H}_t(x, y) = d^2(x, y) \tag{22}$$

where $\mathcal{H}_t$ denotes the heat kernel at time t and $d(x, y)$ the geodesic distance.

6 On the limitations of optimal transport for recovering cell cycles

As demonstrated on toy data and the Proliferative Ductal cell population of the delevoping pancreas in Supplementary Figure 23, optimal transport, and hence moscot, is able to identify the correct direction of cell cycles. Yet, given the cell cycle model in Schwabe et al. [30], there are limitations of the use of optimal transport to recover cell cycles. Indeed, the distributional shift between two time points must be sufficiently small such that cells advance less than 180 degrees (in expectation) in the cell cycle. Note that the advancement in the cell cycle is due to the progression in the non-cycling dimensions of the cell trajectory. This limitation is due to a limited number of samples and moscot relying on discrete optimal transport, and hence not being able to recover the continuous trajectory of cells, but only the (stochastic) conditional distribution.

7 Differential accessibility analysis

To study the ATAC profile of the respective progenitor cells, we performed a differentially accessible peak analysis of epsilon progenitors to arrive at marker peaks using Signac (Methods, Supplementary Tables 30-33). The most highly ranked peak (adjusted p-value of $3.53 \cdot 10^{-52}$) overlaps with the coding regions of *Lrrtm3* and *Ctnna3* (Supplementary Fig. 31), genes which the peak also shares a high peak-gene correlation with (0.20 and 0.18, respectively). The peak is accessible by the epsilon progenitor population as well as epsilon cells. Importantly, the same peak is the 4th most significant peak when differentially testing for the Fev+ Delta cell population (adjusted p-value of $7.27 \cdot 10^{-22}$), confirming the similarity of fate potentials of these cell states. Similar observations for the most notable peaks in the Fev+ delta (Fig. 5m, Supplementary Fig. 31), respectively. The most significantly accessible peak for the Fev+ Delta population (summarizing Fev+ Delta,0 and Fev+ Delta,1 subpopulations to get a larger number of cells) is a peak in the coding region of the gene *Plcb4* (adjusted p-value of $3.49 \cdot 10^{-28}$.). It is also highly accessible for cells classified as epsilon progenitors, epsilon, and delta cells (Supplementary Fig. 31). Similarly, the gene expression of *Plcb4* is particular high in these cell types and it shares the highest peak-gene correlation (0.08). These observations support our lineage hypotheses and conjecture of high plasticity of Fev+ delta cells formulated above.

8 Motif activity analysis in pancreatic endocrinogenesis

8.1 Motif activity in delta cells

For delta cells, the most highly ranked driver TF identified with moscot is *Hhex* (Pearson correlation coefficient 0.66) but no motif associated with *Hhex* was highly expressed (we hypothesize this to be

the case as there is no directly measured motif in the cisBP data base (Methods) and the inferred ones suffer from low quality). The second most remarkable is *Mef2c* (0.44), followed by *Arg1* (0.37) and *Isl1* (0.24). The most remarkable motif according to the Wilcoxon test has cisBP identifier M09209_2.00, with an adjusted p-value of $4.43 \cdot 10^{-66}$. It is directly measured, and associated with *Isl1*. Hence, we report this motif as the marker motif for delta cells.

8.2 Motif activity in epsilon cells

For epsilon cells, motifs related to the *Foxa* family were particularly differentiably accessible, while *Arg1* was identified as the most highly ranked driver TF by moscot, yet, cisBP does not provide an associated motif for this TF. Thus, we identified *Tead1* (correlation of 0.15) as key TF because the directly measured motif *M09438_2.00* is also substantially accessible with an adjusted p-value of $2.29 \cdot 10^{-28}$. *Tead1*, a Hippo signalling effector, is known for its role in early fate decisions of endocrine progenitors, hence it is highly expressed in Ductal cells and Ngn3 low cells[31]. As the differential motif activity test was performed on the whole dataset, the motif score is negative for the cell types shown in the plots. Yet, the expression of *Tead1* decreases substantially along the Ngn3 high,0 / Fev+ lineage, while it remains high in the Ngn3 high,1 / epsilon progenitor lineage.

8.3 Motif activity in alpha cells

Concerning alpha cells, motifs of the *Pax2*/*Pax6* family were particularly active, but only gene expression of *Pax6* is significantly measured. Thus, we chose the motif *M03900_2.00* (adjusted p-value of 0.0), a motif only associated with the TF *Pax6*, while the two more highly scored motifs are associated with both *Pax6* and *Pax2*. *Pax6* is also among the marker TFs identified with moscot (0.25). In accordance with our findings, *Pax6* has been reported to be crucial for the development of alpha cells[32].

8.4 Motif activity in beta cells

For beta cells, *Mafa* is the most highly correlated transcription factor identified with moscot (0.67), while the most active motif according to the Wilcoxon test is *M08835_2.00*, directly measured with *Mafg*, and moreover associated with [*Mafk*, *Mafa*, *Mafg*, *Maf*, *Mafb*]. As *Mafg* is also recovered as a marker feature with moscot (0.29), we identify *Mafg* and the aforementioned motif with cisBP identifier *M08835_2.00* as marker features for beta cells.

References

[1] Gabriel Peyré, et al. Gromov-wasserstein averaging of kernel and distance matrices. In *International Conference on Machine Learning*, pages 2664–2672. PMLR, 2016.

[2] Jean-David Benamou, et al. Iterative bregman projections for regularized transportation problems. *SIAM Journal on Scientific Computing*, 37(2):A1111–A1138, 2015.

[3] Marco Cuturi. Sinkhorn distances: Lightspeed computation of optimal transport. *Advances in neural information processing systems*, 26, 2013.

[4] Mor Nitzan, et al. Gene expression cartography. *Nature*, 576(7785):132–137, 2019.

[5] Titouan Vayer, et al. Fused gromov-wasserstein distance for structured objects. *Algorithms*, 13(9):212, 2020.

[6] Meyer Scetbon, et al. Low-rank sinkhorn factorization. In *International Conference on Machine Learning*, pages 9344–9354. PMLR, 2021.

[7] Meyer Scetbon, et al. Linear-time gromov wasserstein distances using low rank couplings and costs. *arXiv preprint arXiv:2106.01128*, 2021.

[8] Ron Zeira, et al. Alignment and integration of spatial transcriptomics data. *Nature Methods*, 19(5): 567–575, 2022.

[9] Ainesh Bakshi and David Woodruff. Sublinear time low-rank approximation of distance matrices. *Advances in Neural Information Processing Systems*, 31, 2018.

[10] Pitor Indyk, et al. Sample-optimal low-rank approximation of distance matrices. In *Conference on Learning Theory*, pages 1723–1751. PMLR, 2019.

[11] Marco Cuturi, et al. Optimal transport tools (ott): A jax toolbox for all things wasserstein. *arXiv preprint arXiv:2201.12324*, 2022.

[12] Jason Altschuler, et al. Massively scalable sinkhorn distances via the nyström method. *Advances in neural information processing systems*, 32, 2019.

[13] Christopher Williams and Matthias Seeger. Using the nyström method to speed up kernel machines. *Advances in neural information processing systems*, 13, 2000.

[14] Aden Forrow, et al. Statistical optimal transport via factored couplings. In *The 22nd International Conference on Artificial Intelligence and Statistics*, pages 2454–2465. PMLR, 2019.

[15] George K Gittes. Developmental biology of the pancreas: a comprehensive review. *Developmental biology*, 326(1):4–35, 2009.

[16] Eckhard Lammert, et al. Role of endothelial cells in early pancreas and liver development. *Mechanisms of development*, 120(1):59–64, 2003.

[17] Hjalte List Larsen and Anne Grapin-Botton. The molecular and morphogenetic basis of pancreas organogenesis. In *Seminars in cell & developmental biology*, volume 66, pages 51–68. Elsevier, 2017.

[18] Romain Lopez, et al. Deep generative modeling for single-cell transcriptomics. *Nature methods*, 15(12): 1053–1058, 2018.

[19] Leland McInnes, et al. Umap: Uniform manifold approximation and projection for dimension reduction. *arXiv preprint arXiv:1802.03426*, 2018.

[20] Laurens Van der Maaten and Geoffrey Hinton. Visualizing data using t-sne. *Journal of machine learning research*, 9(11), 2008.

[21] Laleh Haghverdi, et al. Diffusion maps for high-dimensional single-cell analysis of differentiation data. *Bioinformatics*, 31(18):2989–2998, 2015.

[22] Laleh Haghverdi, et al. Diffusion pseudotime robustly reconstructs lineage branching. *Nature methods*, 13(10):845–848, 2016.

[23] Sean C Bendall, et al. Single-cell trajectory detection uncovers progression and regulatory coordination in human b cell development. *Cell*, 157(3):714–725, 2014.

[24] Manu Setty, et al. Characterization of cell fate probabilities in single-cell data with palantir. *Nature biotechnology*, 37(4):451–460, 2019.

[25] Sophie Tritschler, et al. Concepts and limitations for learning developmental trajectories from single cell genomics. *Development*, 146(12):dev170506, 2019.

[26] Keenan Crane, et al. The heat method for distance computation. *Communications of the ACM*, 60(11): 90–99, 2017.

[27] Guillaume Huguet, et al. Geodesic sinkhorn: optimal transport for high-dimensional datasets. *arXiv preprint arXiv:2211.00805*, 2022.

[28] Justin Solomon, et al. Convolutional wasserstein distances: Efficient optimal transportation on geometric domains. *ACM Transactions on Graphics (ToG)*, 34(4):1–11, 2015.

[29] Sathamangalam R Srinivasa Varadhan. On the behavior of the fundamental solution of the heat equation with variable coefficients. *Communications on Pure and Applied Mathematics*, 20(2):431–455, 1967.

[30] Daniel Schwabe, et al. The transcriptome dynamics of single cells during the cell cycle. *Molecular systems biology*, 16(11):e9946, 2020.

[31] Inês Cebola, et al. Tead and yap regulate the enhancer network of human embryonic pancreatic progenitors. *Nature cell biology*, 17(5):615–626, 2015.

[32] Luc St-Onge, et al. Pax6 is required for differentiation of glucagon-producing α-cells in mouse pancreas. *Nature*, 387(6631):406–409, 1997.
