# Supplementary material for: Mapping cells through time and space with moscot
Source: Nature. 2025 Jan 22;638(8052):1065–75. doi: 10.1038/s41586-024-08453-2 (PMC11864987; doi:10.1038/s41586-024-08453-2)
Supplement: Supplementary file 2 — Reporting Summary [file 41586_2024_8453_MOESM2_ESM.pdf]

nature portfolio

Corresponding author(s): Fabian Theis

Last updated by author(s): Aug 18, 2024

Reporting Summary

Nature Portfolio wishes to improve the reproducibility of the work that we publish. This form provides structure for consistency and transparency in reporting. For further information on Nature Portfolio policies, see our Editorial Policies and the Editorial Policy Checklist.

Statistics

For all statistical analyses, confirm that the following items are present in the figure legend, table legend, main text, or Methods section.

n/a	Confirmed	
☐	☒	The exact sample size (*n*) for each experimental group/condition, given as a discrete number and unit of measurement
☒	☐	A statement on whether measurements were taken from distinct samples or whether the same sample was measured repeatedly
☐	☒	The statistical test(s) used AND whether they are one- or two-sided<br>*Only common tests should be described solely by name; describe more complex techniques in the Methods section.*
☐	☒	A description of all covariates tested
☐	☒	A description of any assumptions or corrections, such as tests of normality and adjustment for multiple comparisons
☐	☒	A full description of the statistical parameters including central tendency (e.g. means) or other basic estimates (e.g. regression coefficient) AND variation (e.g. standard deviation) or associated estimates of uncertainty (e.g. confidence intervals)
☐	☒	For null hypothesis testing, the test statistic (e.g. *F*, *t*, *r*) with confidence intervals, effect sizes, degrees of freedom and *P* value noted<br>*Give P values as exact values whenever suitable.*
☒	☐	For Bayesian analysis, information on the choice of priors and Markov chain Monte Carlo settings
☒	☐	For hierarchical and complex designs, identification of the appropriate level for tests and full reporting of outcomes
☐	☒	Estimates of effect sizes (e.g. Cohen's *d*, Pearson's *r*), indicating how they were calculated

Our web collection on statistics for biologists contains articles on many of the points above.

Software and code

Policy information about availability of computer code

Data collection	No software was used

Data analysis	Python 3.11<br>moscot 0.3.4<br>ott-jax=0.4.5<br>scanpy=1.9.4<br>pywavelets=1.5.0<br>squidpy=1.3.1<br>cellrank=2.0.0<br>anndata=0.9.2<br>muon=0.1.5<br>mudata=0.2.3<br>scvelo=0.2.5<br>pygpcca=1.0<br>r-shiny=1.8.<br>signac=2.1.0<br>r-seurat=4.4.0<br><br>reproducibility: https://github.com/theislab/moscot-framework_reproducibility

For manuscripts utilizing custom algorithms or software that are central to the research but not yet described in published literature, software must be made available to editors and reviewers. We strongly encourage code deposition in a community repository (e.g. GitHub). See the Nature Portfolio guidelines for submitting code & software for further information.

Data

Policy information about availability of data

All manuscripts must include a data availability statement. This statement should provide the following information, where applicable:

- Accession codes, unique identifiers, or web links for publicly available datasets
- A description of any restrictions on data availability
- For clinical datasets or third party data, please ensure that the statement adheres to our policy

The mouse embryogenesis atlas by Qiu et al is available at http://tome.gs.washington.edu. The mouse liver CITE-seq by Guilliams et al. is available at https://www.livercellatlas.org/. The Vizgen MERSCOPE Liver and Brain coronal sections dataset is available at the Vizgen public dataset release website https://vizgen.com/data-release-program/. The datasets for benchmarking the spatial mapping problems were taken from the original publication of Li et al. The spatiotemporal atlas of mouse embryogenesis (MOSTA) by Chen et al is available at https://db.cngb.org/stomics/mosta/. The spatiotemporal drosophila dataset is available at https://db.cngb.org/stomics/flysta3d/. The pancreas data by Basitdas-Ponce et al. is available under GEO accession code GSE132188 at https://www.ncbi.nlm.nih.gov/geo/query/acc.cgi?acc=GSE132188. The pancreas multiome data is available under GEO accession code GSE275562.

Research involving human participants, their data, or biological material

Policy information about studies with human participants or human data. See also policy information about sex, gender (identity/presentation), and sexual orientation and race, ethnicity and racism.

Reporting on sex and gender	N/A
Reporting on race, ethnicity, or other socially relevant groupings	N/A
Population characteristics	N/A
Recruitment	N/A
Ethics oversight	N/A

Note that full information on the approval of the study protocol must also be provided in the manuscript.

Field-specific reporting

Please select the one below that is the best fit for your research. If you are not sure, read the appropriate sections before making your selection.

☒ Life sciences ☐ Behavioural & social sciences ☐ Ecological, evolutionary & environmental sciences

For a reference copy of the document with all sections, see nature.com/documents/nr-reporting-summary-flat.pdf

Life sciences study design

All studies must disclose on these points even when the disclosure is negative.

Sample size	Sample size was determined based on the available experimental data.
Data exclusions	Data exclusion based on standard single-cell preprocessing pipelines.
Replication	Replication was not feasible due to financial reasons
Randomization	no experimental groups
Blinding	no experimental groups

Reporting for specific materials, systems and methods

We require information from authors about some types of materials, experimental systems and methods used in many studies. Here, indicate whether each material, system or method listed is relevant to your study. If you are not sure if a list item applies to your research, read the appropriate section before selecting a response.

Materials & experimental systems

n/a	Involved in the study
☐	☒ Antibodies
☐	☒ Eukaryotic cell lines
☒	☐ Palaeontology and archaeology
☐	☒ Animals and other organisms
☒	☐ Clinical data
☒	☐ Dual use research of concern
☒	☐ Plants

Methods

n/a	Involved in the study
☒	☐ ChIP-seq
☒	☐ Flow cytometry
☒	☐ MRI-based neuroimaging

Antibodies

Antibodies used	goat anti-somatostatin, 1:300, polyclonal (D-20) (Santa Cruz Biotechnology, SC-7819)<br>Mouse anti ghrelin, 1:250, monoclonal (2F4) (Santa Cruz Biotechnology, SC-293422)
Validation	These are commercially available antibodies and their specificities have been tested using primary human islets.

Eukaryotic cell lines

Policy information about cell lines and Sex and Gender in Research

Cell line source(s)	Human iPSC cell lines: C-peptide-mCherry reporter hiPSC line (HMGUi001-A-8) (Siehler et al., 2021) and NEUROD2nVenus/nVenus iPSC clones (HMGUi001-A-42) (Cota et al., 2023) were used.
Authentication	No cell line was authenticated
Mycoplasma contamination	All used cell lines were routinely tested to ensure that they were negative for mycoplasma.
Commonly misidentified lines<br>(See ICLAC register)	one of the used cell lines are reported in ICLAC register

Animals and other research organisms

Policy information about studies involving animals; ARRIVE guidelines recommended for reporting animal research, and Sex and Gender in Research

Laboratory animals	The embryonic stages of the used embryos are embryonic days (E) E14.5, E15.5 and E16.5. The strain of the mouse line Ngn3-Venus fusion was on mixed background (C57BL/6J × 129/SvJ). The mice were kept at the central facilities at Helmholtz Center Munich (HMGU) under Specific-pathogen-free (SPF) conditions. Animal rooms had a light cycle of 12/12 h, temperature of 20–24 C and humidity of 45–65%. Mice received sterile filtered water and standard diet for rodents ad libitum.
Wild animals	No wild animals were used in this study

Reporting on sex	Since we used only embryonic samples there is no information on the sex of the animals.
Field-collected samples	The study did not include field collected samples.
Ethics oversight	Animal studies were conducted with adherence to relevant ethical guidelines for the use of animals in research in agreement with German animal welfare legislation with the approved guidelines of the Society of Laboratory Animals (GV-SOLAS) and the Federation of Laboratory Animal Science Associations (FELASA). The study was approved by the Helmholtz Munich Animal Welfare Body and by the Government of Upper Bavaria.

Note that full information on the approval of the study protocol must also be provided in the manuscript.

Plants

Seed stocks	N/A
Novel plant genotypes	N/A
Authentication	N/A
